# Network methods for describing sample relationships in genomic datasets: application to Huntington’s disease

**DOI:** 10.1186/1752-0509-6-63

**Published:** 2012-06-12

**Authors:** Michael C Oldham, Peter Langfelder, Steve Horvath

**Affiliations:** 1 Department of Neurology, The Eli and Edythe Broad Center of Regeneration Medicine and Stem Cell Research, University of California, San Francisco, USA; 2 Department of Human Genetics, University of California, Los Angeles, USA; 3 Department of Biostatistics, University of California, Los Angeles, CA, USA

**Keywords:** Sample networks, Sample network analysis, Huntington’s disease, Clustering coefficient, cor(K,C), Standardized C(k) curve, Data pre-processing, Microarrays, Gene expression

## Abstract

**Background:**

Genomic datasets generated by new technologies are increasingly prevalent in disparate areas of biological research. While many studies have sought to characterize relationships among genomic features, commensurate efforts to characterize relationships among biological samples have been less common. Consequently, the full extent of sample variation in genomic studies is often under-appreciated, complicating downstream analytical tasks such as gene co-expression network analysis.

**Results:**

Here we demonstrate the use of network methods for characterizing sample relationships in microarray data generated from human brain tissue. We describe an approach for identifying outlying samples that does not depend on the choice or use of clustering algorithms. We introduce a battery of measures for quantifying the consistency and integrity of sample relationships, which can be compared across disparate studies, technology platforms, and biological systems. Among these measures, we provide evidence that the correlation between the connectivity and the clustering coefficient (two important network concepts) is a sensitive indicator of homogeneity among biological samples. We also show that this measure, which we refer to as *cor*(*K*,*C*), can distinguish biologically meaningful relationships among subgroups of samples. Specifically, we find that *cor*(*K*,*C*) reveals the profound effect of Huntington’s disease on samples from the caudate nucleus relative to other brain regions. Furthermore, we find that this effect is concentrated in specific modules of genes that are naturally co-expressed in human caudate nucleus, highlighting a new strategy for exploring the effects of disease on sets of genes.

**Conclusions:**

These results underscore the importance of systematically exploring sample relationships in large genomic datasets before seeking to analyze genomic feature activity. We introduce a standardized platform for this purpose using freely available R software that has been designed to enable iterative and interactive exploration of sample networks.

## Background

Genomic studies capture an enormous amount of information about the molecular organization of biological systems. Understanding this organization poses a challenge for biologists. In most genomic studies, the number of features (gene expression levels, methylation status, protein abundance, etc.) far exceeds the number of biological samples under investigation. Consequently, while network methods are often used to illuminate patterns among pairwise relationships of genomic features, the rich information contained in the connectivity patterns among samples remains comparatively untapped. However, patterns of co-variation in genomic feature activity ultimately reflect heterogeneity among biological samples. It is therefore critical to understand the extent of sample heterogeneity before analyzing genomic feature activity, and whenever possible to relate sample heterogeneity to known sample traits, which may include both biological and technical sources of variation. In practice, biologists’ efforts to explore sample relationships in genomic studies are an integral component of data pre-processing, yet they are often performed in a perfunctory fashion using platform-specific and qualitative criteria.

A popular approach for exploring sample relationships is cluster analysis. Cluster analysis is appealing for its intuitive nature, and is typically used for sample outlier detection, identification of globally distinct subgroups of samples, and identification of distinct subgroups of samples using pre-selected lists of features (e.g. genes, voxels, etc.) [1-4]. Although widely used, cluster analysis suffers from several shortcomings that are often under-appreciated by biologists. Besides depending on the measure used to quantify similarities among samples, the results of cluster analysis can depend heavily on the specific clustering algorithm that is employed. For example, dendrograms produced by hierarchical clustering algorithms acting on the same data may look quite different depending on whether single, average, or complete linkage is used to calculate distances between clusters [2,5,6]. Other clustering procedures may involve additional parameter choices that can have a substantial effect on cluster assignments (e.g. the choice of *k* in *k*-means clustering) [1,5]. Finally, cluster analysis can be impractical for very large datasets, in which the sheer number of samples obscures the organization and characteristics of a dendrogram and produces ambiguous cluster boundaries.

In this study we explore alternative means of describing sample relationships in topological terms by transforming a (dis-)similarity matrix into a network adjacency matrix. Our correlation-based sample network can be interpreted as a polynomial kernel, which implies that the symmetric adjacency matrix is positive semi-definite. Many methods exist to address the challenge of mapping biological and genomic information to kernel matrices [7,8]. Kernel methods involving genomic similarity measures are the basis of many statistical analytic methods such as nonparametric regression, mixed models, hierarchical regression models, score statistics, and support vector machines [9]. Our primary approach in this study uses a signed weighted correlation network, since the resulting kernel i) works well in practice, as shown in our applications, and ii) allows for a geometric interpretation of network concepts [10].

The approach we describe here is a useful complement to cluster analysis, but does not actually require that cluster analysis be performed. A novel feature of our approach is that we show how distinctions among subgroups of samples can be identified using topological measures (both globally and for subsets of genes), which are based on network concepts. Network concepts include the connectivity (which quantifies the strength of each node’s connections with its neighbors) and the clustering coefficient (which quantifies the strength of each node’s neighbors’ connections with each other) [11]. The definitions of these and many other important network concepts are reviewed below and elsewhere [10,12,13].

We illustrate our approach using microarray data generated from multiple human brain regions of control (CTRL) subjects and patients with Huntington’s disease (HD) [14]. HD is a progressive and incurable neurodegenerative disorder characterized by preferential destruction of medium spiny neurons in the striatum [15] and caused by a CAG-repeat expansion in the coding region of the huntingtin gene, which is thought to confer a toxic gain-of-function to the mutant huntingtin protein [16]. Alterations in gene expression are considered a central feature of HD pathology, and the extent to which specific gene expression changes precede disease pathology is an area of active investigation [14,17-20]. Our results indicate that HD exerts a profound effect on sample network topology in the caudate nucleus relative to other (less affected) brain regions. Specifically, we find that the relationship between the standardized sample connectivity and the standardized sample clustering coefficient follows a simple scaling law in unaffected brain regions, but undergoes a sharp transition for HD caudate nucleus samples that reflects the degradation of sample correlation network structure in this brain region. By restricting sample network construction to modules (subsets) of genes that are naturally co-expressed in human caudate nucleus [21], we find that this degradation is most significant in a neuronal signal transduction module. Our findings demonstrate that sample networks can enhance the results of cluster analysis not only with respect to relatively simple tasks such as outlier identification, but also with respect to more complex challenges such as group comparisons.

## Results

The approach we describe in this study formalizes and expands upon a strategy that has previously been used to identify outlying samples in microarray data generated from human brain tissue [21]. Our approach is applicable whenever a dissimilarity or similarity measure can be defined between samples (see Additional file 1). A major advantage of defining a network adjacency measure between samples (as opposed to a general similarity measure) is that it permits specification of network concepts. In our implementation, we define adjacencies among samples as signed weighted correlations with values that approximate the underlying correlations when these correlations are large, as is usually the case in sample networks (Methods). A signed weighted correlation network is attractive since it preserves sign information, is robust with respect to the soft threshold (power) parameter (β), and preserves the continuous nature of correlations (i.e. the result is a fully connected network in which all nodes are neighbors with one another) [22]. In addition, a signed correlation network is equivalent to a network based on the Euclidean distance between scaled vectors (as described in Additional file 1).

### Dataset

The proposed framework for sample network exploration (Methods) was used to analyze microarray data from “the HD study” [14]. These data were generated from brain samples of patients with HD (n = 44 individuals) and unaffected controls (n = 36 individuals, matched for age and sex) [14]. The authors of this study used Affymetrix U133A microarrays to survey gene expression in caudate nucleus (CN), cerebellum (CB), primary motor cortex (Brodmann’s area 4; BA4), and prefrontal cortex (Brodmann’s area 9; BA9) in the CTRL group and across five grades of HD severity, which were scored between 0 (least severe) and 4 (most severe) using Vonsattel’s neuropathological criteria [23]. HD causes extensive neurodegeneration in the CN, where medium spiny neurons are preferentially destroyed in early stages of the disease [15,23]; comparatively, the other analyzed brain regions are relatively spared. In addition to disease status and severity, sample information included age, sex, the country where the experiment was performed (samples were processed in the United States and New Zealand), and the microarray hybridization batch (Additional file 2) [14]. In light of these myriad biological and technical sources of variation, this dataset presents a challenging analytical task.

### A motivational example

**Figure 1:**
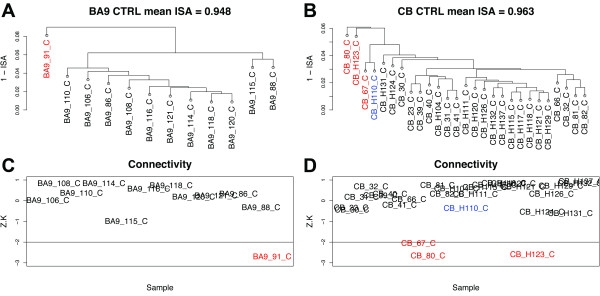
**Network concepts provide a natural framework for describing relationships among samples in high-dimensional biological datasets.** A motivational example. **(A)** Dendrogram produced by average linkage hierarchical clustering using 1 – ISA (intersample adjacency) for a subset of samples (prefrontal cortex [BA9] of CTRL subjects) from ref. [14]. **(B)** Dendrogram produced by average linkage hierarchical clustering using 1 – ISA for another subset of samples (cerebellum [CB] of CTRL subjects) from ref. [14]. **(C)** Standardized sample connectivities (*Z.K*) provide a different view of the BA9 CTRL samples. BA9_91_C (red) exhibited significantly lower connectivity than the other samples in this group, consistent with the dendrogram (**A**). **(D)** Standardized sample connectivities for the CB CTRL samples. Three samples (CB_80_C, CB_H123_C, and CB_67_C, in red) had *Z.K* values that were significantly lower than the others. Note that CB_67_C had much lower connectivity than CB_H110_C (blue), yet these two samples were indistinguishable in the dendrogram above (**B**). Black horizontal lines in (**C**) and (**D**) correspond to an optional *Z.K* threshold (here −2) for outlier removal; CTRL = control.

Below we provide an example that illustrates how network concepts can be used to distinguish samples when hierarchical clustering cannot. Figure 1A depicts a subset of samples from BA9 of CTRL subjects from the HD study. As seen in this example, visual inspection of the dendrogram is sufficient to discern the outlying sample (BA9_91_C). However, it is illustrative to consider an alternative depiction of sample relationships using the network concept of standardized connectivity. Standardized connectivity (*Z.K*; Methods) is a quantity that describes the overall strength of connections between a given node and all of the other nodes in a network. As seen in Figure 1C, the standardized connectivity of sample BA9_91_C is significantly lower than all of the other samples, confirming its status as an outlier in the group. It is important to note, however, that the distribution of standardized connectivities is independent of the choice or use of clustering procedures.

Figure 1B shows the dendrogram produced by hierarchical clustering of another subset of samples from the HD study (CB of CTRL subjects). Here the dendrogram is more complex, with at least two samples (CB_80_C and CB_H123_C) that appear to be outliers, and others that are questionable. If the same samples are depicted in terms of *Z.K* (Figure 1D), it is evident that three samples (CB_80_C, CB_H123_C, and CB_67_C) have *Z.K* values that are significantly lower than the other samples in the group. However, note that CB_H110_C, which is indistinguishable from CB_67_C in the dendrogram above (Figure 1B), has much higher *Z.K* than CB_67_C, indicating that CB_67_C is an outlier whereas CB_H110_C is not. By establishing a threshold (e.g. *Z.K* = −2), standardized connectivity distributions can be used in a quantitative and unbiased fashion to identify and remove outlying samples, which may reflect hidden factors that can influence the results of genomic experiments [24] (this approach is particularly useful when the number of samples is large, making it difficult to distinguish outlying samples in a dendrogram). Analogously, one can also make use of other network concepts as described below.

### Degradation of sample network topology in caudate nucleus by Huntington’s disease

We used the SampleNetwork R function to process all 201 samples from the HD study simultaneously. As seen in Figure S1 (Additional file 1) and our R tutorial (Additional file 3 and http://www.genetics.ucla.edu/labs/horvath/CoexpressionNetwork/SampleNetwork), we observed a dominant effect of brain region on gene expression that was driven largely by the fact that gene expression in each non-cortical (CN and CB) brain region was quite distinct from gene expression in cortical (BA4 and BA9) brain regions, as has been described previously [25-28]. In light of the strong effect of brain region on gene expression, as well as the fact that HD preferentially targets CN relative to the other analyzed brain regions, we next used SampleNetwork to examine samples from each brain region separately. Within each brain region, we analyzed CTRL and HD samples as a single cohort, but note that alternative strategies (e.g., analyzing CTRL and HD samples as separate cohorts) may be desirable, depending on the downstream application.

After constructing sample networks for each brain region (as described in Additional file 3), we examined the relationship between the standardized sample connectivity (*Z*.*K*) and the standardized sample clustering coefficient (*Z*.*C*) for all samples in each brain region. We refer to this relationship as the standardized *C*(*k*) curve. As discussed below, (unstandardized) *C*(*k*) curves have been used to study the topological properties of scale-free networks and other large complex networks [29-32]. We propose using the Spearman correlation to measure the standardized *C*(*k*) curve since it is invariant with regard to monotonically increasing transformations. In particular, the Spearman correlation between *Z.K* and *Z.C* equals that of the unstandardized measures, which is why we denote it simply by *cor*(*KC*) (Methods). In the following, we will demonstrate that the standardized *C*(*k*) curve is a valuable tool for i) assessing the overall consistency of sample behavior within a dataset, ii) identifying distinct groups of samples, and iii) identifying important subsets of features (e.g. genes).

**Figure 2:**
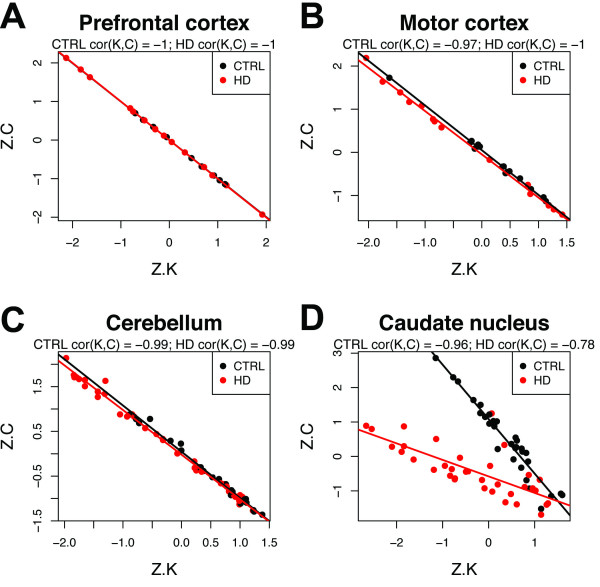
**Sample network concepts reveal the profound effect of Huntington’s disease in caudate nucleus.** Comparison of standardized sample connectivities (*Z.K*) and standardized clustering coefficients (*Z.C*) between control subjects (CTRL) and subjects with Huntington’s disease (HD) in prefrontal cortex **(A**; n = 9 CTRL and 16 HD**)**, motor cortex **(B**; n = 16 CTRL and 14 HD**)**, cerebellum **(C**; n = 23 CTRL and 34 HD**)**, and caudate nucleus **(D**; n = 31 CTRL and 35 HD**)**. Networks were constructed over all probe sets (n = 18,631) using all samples (CTRL and HD) from each brain region.

For samples from prefrontal cortex (Figure 2A), motor cortex (Figure 2B), and cerebellum (Figure 2C), we observed that *Z.K* and *Z.C* formed nearly perfect inverse relationships, with no obvious distinctions between CTRL and HD subjects. In contrast, samples from the caudate nucleus exhibited clear segregation according to diagnosis, with CTRL and HD subjects forming two distinct groups (Figure 2D). This segregation indicates that *cor*(*KC*) is a useful network concept that measures an important aspect of the global architecture in weighted sample networks. Interestingly, *cor*(*KC*) for HD CN samples differed when brain regions were analyzed together (*cor*(*KC*) = 0.77, *P* = 1.7e–08; Figure S1D; Additional file 1) and when they were analyzed apart (*cor*(*KC*) = −0.78, *P* = 4.0e–08; Figure 2D), suggesting that the relationship between the node-based measures *Z.K* and *Z.C* depends upon properties of the network as a whole, a topic that has been the subject of recent investigations [33].

### Understanding the properties of the standardized *C*(*k*) curve

As discussed below, the *C*(*k*) curve has been studied primarily in biological networks in which nodes correspond to gene products [30,32]. In contrast to the *negative* relationship observed in sample networks (Figure 2), we observed that *Z.K* and *Z.C* tended to exhibit a *positive* relationship in gene-based networks (e.g. Figure S2A,B; Additional file 1). A positive relationship was observed for genes that are naturally co-expressed in human caudate nucleus [21] (*cor*(*KC*) = 0.7, *P* < 2.2e-16; Figure S2A,C; Additional file 1), as well as for genes that were selected at random (*cor*(*KC*) = 0.83, *P* < 2.2e-16; Figure S2B,D; Additional file 1). To understand why *cor*(*KC*) is often positive in gene-based networks but negative in sample networks, consider that in most microarray studies, and in particular when analyzing similar biological specimens, samples are highly correlated with one another (e.g. *r* > 0.95 when measured across all genes). In contrast, most genes exhibit moderate to weak correlations with other genes, such that the mean correlation in a typical gene co-expression network is close to 0 and follows an approximately normal distribution (e.g. Figure S2D; Additional file 1). Even for a module of co-expressed genes, when compared with sample networks, the distribution of pairwise correlations is shifted towards smaller values (e.g. Figure S2C; Additional file 1). Therefore, we hypothesized that the contrasting relationships between *Z.K* and *Z.C* in sample networks and gene networks might relate to differences in the global topological organization of each network.

**Figure 3:**
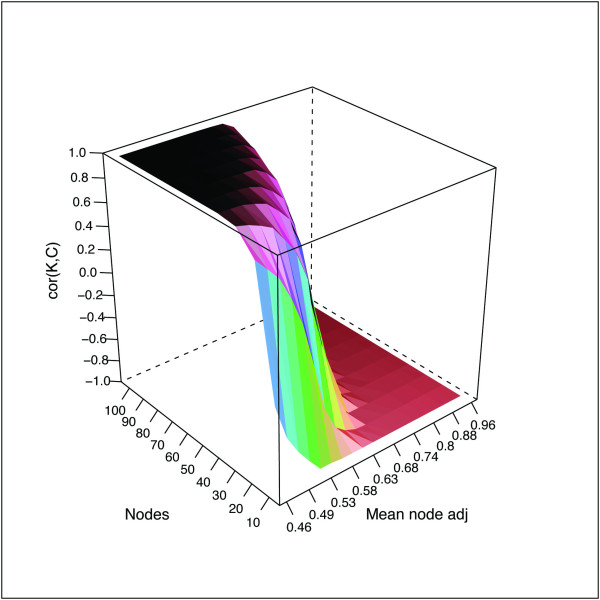
***cor*(*K*,*C*) depends upon network topology and network size.** The Spearman correlation (*cor*(*KC*); z-axis) between the connectivity and the clustering coefficient as a function of network density (mean node adj. [adjacency]; x-axis) and network size (nodes; y-axis). Signed networks (β = 2) were simulated using the simulateModule function from the WGCNA R package [34]. The seed module eigengene (ME) consisted of 5,000 random, normally distributed features (mean = 0, sd = 1). The function parameters “corPower” and “propNegativeCor” were set to 0.75 and 0, respectively. The function parameter “minCor” was iteratively reduced from .95 to .05 by increments of .05, progressively degrading the strength of node connections; for each iteration, *cor*(*KC*) was calculated for module networks of various sizes (n = 10 to 100, by = 10).

To test this hypothesis, we conducted a simulation study to explore the properties of *cor*(*KC*) by systematically varying the network topology (mean node adjacency) and network size (number of nodes). For simulated networks with low mean node adjacency (i.e. mostly weak connections among nodes, like most gene co-expression networks), we observed values of *cor*(*KC*) approaching 1 (Figure 3), indicating a nearly perfect positive linear relationship between Z*.K* and *Z.C*. As the strength of connections among nodes (i.e. mean node adjacency) began to increase, *cor*(*KC*) began to shift, while also revealing a dependence on network size (i.e. number of nodes; Figure 3). This shift accelerated dramatically as simulated networks began to consist of mostly strong connections among nodes, producing a “waterfall” effect reminiscent of a percolation transition [33] (Figure 3). When simulated networks possessed very high mean node adjacency (like most sample networks), *cor*(*KC*) approached −1 (Figure 3), indicating a nearly perfect negative linear relationship between *Z.K* and *Z.C*.

Collectively, these observations suggest that the divergence of *cor*(*K*,*C*) for HD CN samples relative to CTRL samples and other brain regions (Figure S1D [Additional file 1], Figure 2D) reflects a degradation of global sample network topology in CN by HD. To visualize this degradation more directly, we compared the distributions of pairwise sample adjacencies between CTRL and HD subjects for each brain region. The distributions of sample adjacencies exhibited the greatest difference between CTRL and HD subjects in CN, where HD sample adjacencies were markedly degraded (Figure S3; Additional file 1). Thus, degradation of global sample network topology by HD in CN has shifted *cor*(*K*,*C*) for HD CN samples. This relationship has begun to invert (i.e. it is “in the waterfall” [Figure 3]), indicating that HD has initiated a percolation-like transition in the global network topology of CN samples.

### Sample network topology reveals strong effects of Huntington’s disease on specific gene co-expression modules in human caudate nucleus

The degradation of global sample network topology by HD in CN (Figures S1D, 2D, S3) was observed across all analyzed probe sets (n = 18,631). We hypothesized that this effect might vary for specific subsets of genes involved in disparate biological processes, which in turn might implicate specific biological processes in connection with HD pathology. By focusing on pre-selected gene sets (informally referred to as modules), we illustrate below how the standardized *C*(*k*) curve can be used to identify clinically important subsets of features (i.e. genes). Toward this end, we make use of a second R function called ModuleSampleNetwork (and refer to the resulting sample networks as “module sample networks”).

We have previously shown that the transcriptome of normal human CN is organized into modules of co-expressed genes, many of which relate to specific cell types and functional processes [21]. For example, gene co-expression modules corresponding to oligodendrocytes, astrocytes, neurons, mitochondrial function, synaptic function, immune response, gender differences, and the subventricular neurogenic niche have been described in human CN [21]. Subsequent work in rodents has confirmed that striatal gene co-expression network architecture is robust across disparate strains of mice [35]. The inherent organization of the CN transcriptome provides a natural framework in which to study the effects of HD on sample network topology. Therefore, we sought to determine the extent to which variation in sample network topology was associated with particular gene co-expression modules in CN. Specifically, we constructed sample networks in CN for each of the 23 gene co-expression modules that were previously identified in this brain region in humans [21]. The 23 gene co-expression modules are labeled by colors (e.g. the “palegreen” module), with pertinent functional characterizations taken from ref. [21].

**Figure 4:**
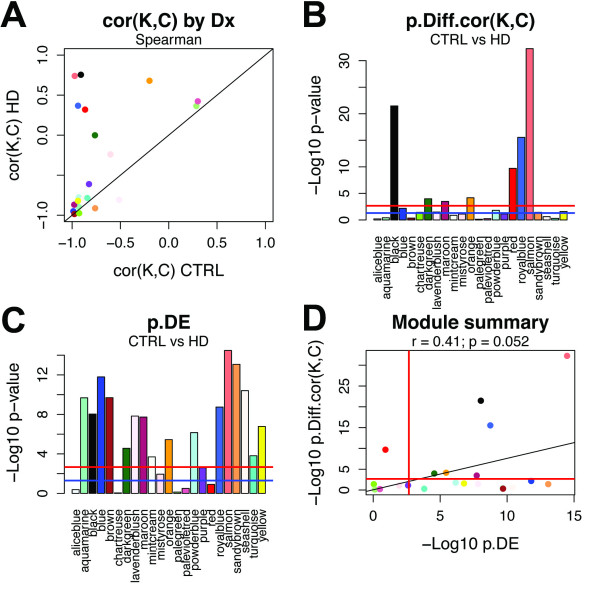
**Huntington’s disease exerts strong effects on specific gene co-expression modules in human caudate nucleus.** Analysis of human caudate nucleus (CN) sample network properties for each of 23 gene co-expression modules previously identified in CN; colors correspond to the original gene co-expression module labels from [21]. **(A)** For each module sample network, the Spearman correlations *cor*(*KC*) are plotted for control (CTRL) and Huntington’s disease (HD) subjects. Each point corresponds to a module. Black line: y = x. **(B)** The log-transformed *P*–value of the difference between *cor*(*KC*) for CTRL and HD subjects is reported for each module (Methods). **(C)** The extent of differential expression (DE) between CTRL and HD was assessed for each module by using Student’s *t*-test of DE for the module eigengene (ME; i.e. the first principal component obtained by singular value decomposition of the module expression matrix) between CTRL and HD. **(D)** Comparison of the module significance levels reported in (**B**) and (**C**); linear least squares regression line in black. p.Diff.cor(K,C) denotes the *P*-value for testing the differences of *cor*(*KC*) between the CTRL and HD module sample networks. (**B–D**) Blue lines: *P* = .05; red lines: Bonferroni correction for multiple comparisons.

To assess the effects of HD on module sample network topology, we calculated *cor*(*K*,*C*) for CTRL and HD subjects in every module (Figure 4A). Based upon the relationship observed between *Z.K* and *Z.C* for CTRL and HD subjects in BA9, BA4, and CB (Figure 2A–C), we hypothesized that in the absence of an effect of HD on module sample network topology, *cor*(*K*,*C*) CTRL should approximately equal *cor*(*K*,*C*) HD. In addition, for module sample networks characterized by strong connections among nodes, we expected *cor*(*K*,*C*) to approach −1 (Figure 3). The majority of modules clustered along the diagonal, indicating relative preservation of *cor*(*K*,*C*) between CTRL and HD subjects; however, a handful of modules were clearly distinguished as outliers (Figure 4A). Among the outliers, the difference in *cor*(*K*,*C*) between CTRL and HD subjects was most significant for the salmon module (M8C), followed by the black (M11C; Figure S4; Additional file 1), royalblue (M36; Figure S5; Additional file 1), and red (M19C; Figure S6; Additional file 1) modules (Figure 4B). These results indicate that *cor*(*K*,*C*) is a useful measure for highlighting differences in sample network topology among subsets of genes.

In the original HD study [14], the authors determined that a large fraction (~20%) of transcripts showed differential expression (DE) in post-mortem CN between CTRL and HD subjects. DE in HD is thought to reflect both cell-intrinsic changes in gene expression (i.e. changes in gene expression induced by the mutant huntingtin protein), as well as changes at the cellular population level due to neuronal cell death and subsequent astrogliosis [14,17,20]. In light of such widespread changes, we asked whether particular gene co-expression modules were associated with DE. As shown in Figure 4C, many modules were significantly associated with DE. This result is perhaps not surprising, inasmuch as cellular stoichiometry is altered by HD and many modules have been shown to be enriched with cell type-specific genes [21]. We next sought to relate the extent of modular DE with the extent of modular degradation in sample network topology. As shown in Figure 4D, the salmon module was the most significant in both of these dimensions, followed by the black and royalblue modules. Overall, however, the relationship between these two measures was weak (r = 0.41, *P* = 5.2e–02). Indeed, one module (red) exhibited a very significant difference in *cor*(*KC*) between CTRL and HD subjects, with no significant evidence of differential expression (Figure 4D).

### *cor*(*K*,*C*) can distinguish sample groups in the absence of differential expression

**Figure 5:**
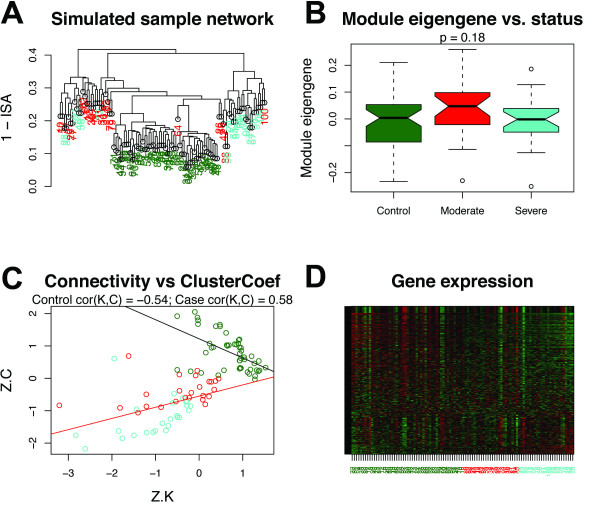
***cor*(*K*,*C*) distinguishes sample subgroups in the absence of differential expression.** Analysis of a simulated gene expression module consisting of 500 genes and 100 samples. Samples were assigned to one of three subgroups based on simulated disease status: “control” (n = 50; darkgreen), “moderate” (n = 25; red), or “severe” (n = 25; turquoise) (Methods). **(A)** Average linkage hierarchical clustering of samples using 1 – ISA (intersample adjacency) as a dissimilarity measure. **(B)** Distributions of module eigengene (ME) values by sample subgroup. Note that these distributions are not significantly different (*P* = 0.18, Kruksal-Wallis test), indicating that there is no differential expression associated with disease status at the modular level. **(C)** When depicted in terms of *Z*.*K* and *Z*.*C*, control and affected subjects segregated into two distinct groups (linear least squares regression lines in black [control] and red [affected]). **(D)** Heat map of simulated gene expression levels. Rows correspond to genes and columns correspond to samples. Green = low expression; red = high expression

To explore the basis for this observation, we conducted a simulation study to determine whether *cor*(*K*,*C*) could distinguish subsets of samples in the absence of differential expression. Specifically, we simulated a set of 500 genes and 100 samples (referred to as a “module”), using the real structure of the red module as an approximate guide (Methods). Samples were assigned to one of three groups using a simulated sample trait (referred to as “disease status”), with 50 samples corresponding to control status, 25 samples corresponding to moderate disease status, and 25 samples corresponding to severe disease status (Methods). The simulation model assumed i) that 60% of the module genes were not related to the disease and ii) that these noise genes had lower mean values than the 40% of (signal) genes that were down-regulated by the disease. Figure 5A depicts the dendrogram produced by hierarchical clustering of sample adjacencies for the simulated module. As seen in Figure 5B, the observed module eigengene was not related to disease status (*P* = 0.18, Kruskal-Wallis test). In contrast, *cor*(*K*,*C*) clearly delineated the control samples from the affected samples (Figure 5C), despite inconsistent gene expression differences among the three sample groups (Figure 5D). These results provide further evidence that *cor*(*K*,*C*) can distinguish meaningful groups of samples in certain situations where differential expression analysis cannot.

### A neuronal signal transduction module is profoundly degraded by Huntington’s disease

**Figure 6:**
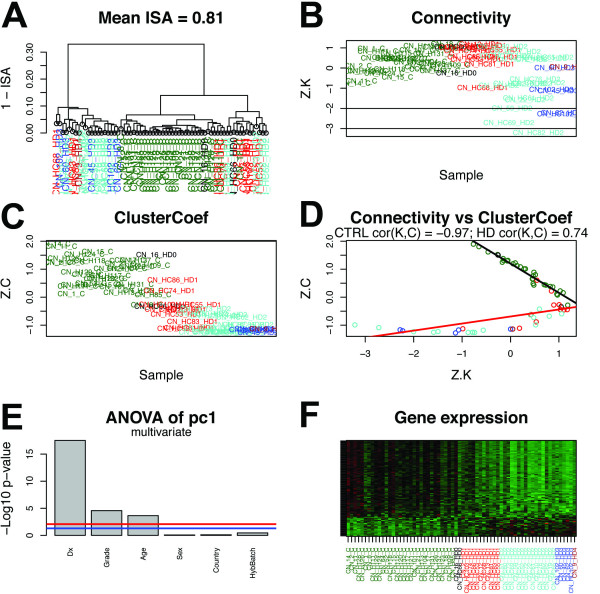
**Caudate nucleus samples exhibit significant segregation by diagnosis in gene co-expression module M8C (salmon).** Analysis of caudate nucleus (CN) sample network properties for genes comprising the CN salmon co-expression module M8C [21]. **(A)** Average linkage hierarchical clustering of samples using 1 – ISA (intersample adjacency) as a dissimilarity measure. Colors denote control (CTRL) subjects (darkgreen; n = 31) and Huntington’s disease (HD) subjects with varying grades of disease severity: HD grade 0 (black; n = 2), HD grade 1 (red; n = 11), HD grade 2 (turquoise; n = 16), HD grade 3 (blue; n = 5), and HD grade 4 (brown; n = 1). Standardized sample connectivities **(***Z.K*; **B)** and standardized sample clustering coefficients **(***Z.C*; **C**). **(D)** HD and CTRL samples segregated into two distinct groups when depicted in terms of *Z.K* and *Z.C* (linear least squares regression line in black [CTRL] and red [HD]). **(E)** Multivariate linear regression revealed a highly significant effect of diagnosis (Dx) on the salmon module eigengene. Blue line: *P* = .05; red line: Bonferroni correction for multiple comparisons. **(F)** Heat map of expression levels for genes comprising the salmon co-expression module M8C. Rows correspond to probe sets (genes) and columns correspond to samples. Green = low expression; red = high expression. Samples in (**B–D, F**) are colored as in (A).

Figure 6 depicts the results of sample network construction for the CN salmon module (similar depictions for the black, royalblue, and red modules can be found in Figures S4, S5, and S6, respectively). Hierarchical clustering of sample adjacencies produced a dendrogram with two large branches (Figure 6A). The first branch formed a cluster comprised exclusively of HD samples (cluster 1), 85% of which were Vonsattel grade 2 or higher (i.e. later stages of disease progression). The second branch subdivided to produce two sample clusters. 91% of the samples in cluster 2 corresponded to unaffected individuals, with the remainder consisting of grade 1 (n = 2) or grade 0 (n = 1) HD samples. Cluster 3 was comprised almost exclusively of HD samples, all of which were grade 2 or below (i.e. earlier stages of disease progression).

Examination of the distribution of *Z.K* among samples in the salmon module (Figure 6B) also revealed a distinction among grades of HD severity. Grade 1 and a subset of grade 2 HD samples possessed *Z.K* values that were comparable to those of unaffected individuals; however, a majority of grade 2 samples and grade 3 samples possessed *Z.K* values that were substantially lower than all other samples (Figure 6B). In contrast, examination of *Z.C* revealed a monotonic arrangement of samples, with CTRL > grade 1 > grade 2 > grade 3 (Figure 6C). When plotted in both of these dimensions, samples formed two distinct lines that clearly delineated CTRL and HD subjects (Figure 6D). Interestingly, three HD samples (two grade 1 and one grade 0) fell upon the same regression line as the CTRL samples (Figure 6D, black line); these were the same samples that belonged to cluster 2 in Figure 6A. It is possible that the intermingling of some early stage HD samples with CTRL subjects could reflect the continuum of neurodegeneration that spans from normal aging to neurodegenerative disease. We also observed that the distribution of HD samples along their regression line tended to reflect their grade of severity (Figure 6D, red line). These results provide visual confirmation of the significant distinction between CTRL and HD subjects in the salmon module reported above (Figure 4A,B). In addition, multivariate linear regression using the salmon module eigengene (i.e. the first principal component of gene expression in the salmon module) as outcome confirmed an extremely significant effect of diagnosis (Dx) on gene expression in this module, as well as significant independent effects for grade and age (Figure 6E). The effect of diagnosis on gene expression was evident when gene expression in the salmon module was visualized directly (Figure 6F).

As can be seen in Figure 6F, the vast majority of genes in the salmon module showed decreased expression levels with increasing severity of HD, which would be expected as a consequence of neuronal cell death (notwithstanding cell-intrinsic changes in gene expression induced by the mutant huntingtin protein). When it was originally described, the salmon gene co-expression module in human CN was found to be enriched with genes that are preferentially expressed in neurons, genes that encode synaptic proteins, and genes involved in signal transduction [21]. Analyses of differential expression, functional enrichment, and membership strength for all genes in the salmon module are summarized in Additional file 4. To dissociate changes in gene expression caused by altered cellular stoichiometry in HD from changes in gene expression caused by cell-intrinsic effects of the mutant huntingtin protein, we cross-referenced CN module composition with a set of genes that has been found to be dysregulated in primary neuron models of HD [20]. In the study by Runne et al., the effects of mutant huntingtin on gene expression were measured *before* cell death in primary striatal neurons cultured from rat brains [20]. We observed that the salmon module was significantly enriched with this set of dysregulated genes, and more so than any other module (Figure S7; Additional file 1). We also note that a number of genes in the salmon module were previously found to be differentially expressed in laser-microdissected striatal neurons of CTRL and HD human subjects [14] (Additional file 4).

Lastly, we used Ingenuity Pathways Analysis (IPA) to determine whether the salmon module was enriched with annotated functional categories of genes. Out of more than 500 annotated functional categories of genes in the IPA database, the two categories that showed the most significant enrichment with genes from the salmon module were “dyskinesia” (FDR *P* = 1.4e–24) and “Huntington”s disease” (FDR *P* = 1.6e–24) (Additional file 5).

## Discussion

To the best of our knowledge, this work provides the first formal demonstration that network methods can distinguish biologically meaningful relationships among samples in genomic datasets. We have shown that sample networks can identify outlying samples when hierarchical clustering procedures cannot, and even when hierarchical clustering procedures are not used at all. We have described a novel network statistic, *cor*(*K*,*C*), and shown that it can be used to i) evaluate sample homogeneity, ii) identify sample characteristics (e.g. diagnosis) with global effects, and iii) enable comparisons among groups of samples using pre-selected lists of features (e.g. gene co-expression modules). By applying the latter approach to microarray data generated from human brain tissue, we have identified a neuronal signal transduction module that is an epicenter of transcriptional dysregulation in striatal samples from individuals with HD. The advantages of using network methods for describing sample relationships in genomic datasets are summarized below.

A major advantage of constructing sample networks is that individual samples can subsequently be described using established node-based network concepts such as the connectivity and the clustering coefficient. These concepts are independent of the choice or use of clustering algorithms and depend only on the adjacency measure used to construct the network. The distributions of standardized node-based network concepts provide an unbiased and quantitative framework for identifying samples that “behave” differently, even if the underlying causes of this behavior are unknown. Intuitively, if the connectivity for a given sample (when measured over all genes) is significantly lower than all other sample connectivities from the same biological system, it suggests that there is something different about that sample compared to the others. The investigator must ask him/herself whether the observed difference is likely to reflect biological or technical variation. In light of the multiple steps that comprise a typical genomic experiment, each of which may introduce technical variation, a conservative approach is to exclude aberrant samples if there are no obvious biological factors that might explain their discordant behavior.

Compared with other methods for identifying outlying samples in genomic data, our approach offers several additional advantages. First, because sample relationships are defined with respect to a correlation matrix, it is platform-agnostic and does not require access to raw data (although in practice it is preferable to process raw data in a consistent fashion). Second, it is easily applied to very large datasets, in contrast to clustering procedures that rely upon visual inspection of dendrograms to identify outlying samples. Third, it produces a battery of measures for summarizing the consistency and integrity of genomic datasets (e.g. mean intersample adjacency [ISA, or density], decentralization, homogeneity, etc.), which can be compared across disparate studies, technology platforms, and biological systems. Such measures are especially useful for meta-analyses, where objective assessment of data quality is highly desirable before seeking to pool or compare results across studies. Finally, as implemented in SampleNetwork and described in Additional file 3, our approach is both flexible and efficient, enabling users to move quickly through large datasets in an iterative fashion, specifying groups of samples for processing, identifying and removing outliers, testing the significance of sample covariates, and performing data normalization. To enhance user-friendliness, we have also incorporated the R function ComBat [36], which is an effective tool for removing batch effects (Additional file 1). At each stage, relevant output files are produced and exported automatically.

At the same time, there are several important caveats associated with our proposed approach for using network concepts to identify outlying samples in genomic data. It should be noted that our approach works best for datasets with large numbers of samples (e.g. more than 10). It is also important to note that standardized network concepts such as *Z.K* are relative measures whose interpretation depends on context. For example, in a relatively homogeneous sample network (e.g. mean ISA > 0.97), a *Z.K* value of −2.5 implies higher adjacencies for the sample in question than it would in a more heterogeneous sample network (e.g. mean ISA < 0.9). In light of these considerations, it can be helpful to have “targets” in mind, such as an expectation of what the mean ISA should approach for a given biological system, technology platform, and adjacency measure. These targets can be guided by prior experience (for example, cancer datasets often exhibit substantial sample heterogeneity) or by the use of technical and biological replicates. Lastly, although we have focused primarily on *Z.K* and to a lesser extent *Z.C* as intuitive indicators of outlying status, it is possible that other node-based network concepts (or indeed, other measures of adjacency) could produce different results.

Beyond facilitating relatively simple tasks such as outlier identification, sample networks provide a novel perspective on more complex challenges such as group comparisons. Our results indicate that the standardized *C*(*k*) curve in weighted sample networks is a powerful tool for identifying sample characteristics with global effects on genomic activity. The stark divergence of *cor*(*K*,*C*) for HD CN samples motivated us to explore how *cor*(*K*,*C*) would be affected by other network topologies, leading to the observation that *cor*(*K*,*C*) undergoes a percolation-like transition that is related to network density and size. Although *cor*(*K*,*C*) was inversely related to network density in our simulations, we note that *cor*(*K*,*C*) is invariant if one scales all off-diagonal adjacencies by a constant. Therefore, it is more accurate to consider *cor*(*K*,*C*) as an indicator of network heterogeneity (or homogeneity; Additional file 1). In the special situation of an exactly factorizable network, we find that *cor*(*K*,*C*) is determined by the network heterogeneity (Methods). One practical implication of these findings is that *cor*(*K*,*C*) can serve as a useful indicator of data “cleanliness”: with each iteration of sample outlier removal or data normalization performed using SampleNetwork, *cor*(*K*,*C*) should approach −1.

We note that our findings with respect to the percolation-like transition for *cor*(*K*,*C*) are also applicable to unweighted (binary) networks. We have observed a similar transition for *cor*(*K*,*C*) in unweighted gene networks as the threshold for dichotomizing the adjacency matrix is progressively increased (Figure S8; Additional file 1). At permissive (low) thresholds, which produce networks in which most nodes are connected, *cor*(*K*,*C*) is negative; as the threshold is raised, producing networks in which most nodes are not connected, the relationship begins to invert, becoming positive at more stringent (high) thresholds (Figure S8; Additional file 1).

In unweighted networks, the relationship between the (unstandardized) connectivity and (unstandardized) clustering coefficient of network nodes, i.e. the *C*(*k*) curve, has previously been reported to follow a scaling law: *C* ≅ *k*^α^[29,31]. It has been shown that the value of the scaling exponent α is not universal, but negative values approaching −1 have been observed in biological systems [30,32]. The inverse relationship for the *C*(*k*) curve has been interpreted as evidence of hierarchical modularity in network structure [30,31]. Specifically, it has been suggested that in hierarchically modular networks, nodes with low connectivity form small, densely connected clusters, while nodes with high connectivity serve to bridge these many small clusters into one large, integrated network [31]. However, the *C*(*k*) curve has primarily been studied in the context of metabolic, protein interaction, and gene regulatory networks, as well as other non-biological networks [30,32,37].

To the best of our knowledge, a percolation-like transition in the *C*(*k*) curve has not previously been reported. However, prior work has revealed that global topological properties of unweighted networks, such as those embodied in the *C*(*k*) curve, can be predicted by knowledge of local motif structure, and vice versa [33]. Motifs, or subgraphs, describe basic interaction patterns among small groups of nodes [38,39]. In unweighted networks, it has been shown that subgraphs naturally segregate into two classes: highly abundant type I subgraphs, which are sparsely interconnected, and less abundant type II subgraphs, which are densely interconnected [33]. It has also been shown that a phase boundary separating type I and type II subgraphs can be accurately predicted using global network topological properties, including the *C*(*k*) curve [33]. Therefore, we propose that the transition in the standardized *C*(*k*) curve observed in our analysis reflects a concomitant transition in local motif structure, which in turn reflects the degradation of sample network topology in CN by HD. Although motifs have been studied almost universally in the context of unweighted networks, we are aware of at least one study that has presented an approach for generalizing motif scoring to weighted networks [40]. Our results suggest that future research investigating the relative strengths of distinct motifs in weighted networks and their relationship to global network topological properties is warranted.

The effect of HD on the standardized *C*(*k*) curve for CN samples was initially observed over all genes, which is consistent with the large impact that HD exerts on the CN transcriptome [14,17,19,20,41]. Because the transcriptomes of human brain regions, including CN, are organized into biologically meaningful gene co-expression modules [21], we reasoned that constructing sample networks for previously identified CN modules might expose variation in the standardized *C*(*k*) curve, which in turn might implicate specific biological processes in connection with HD pathology [42]. This approach constitutes a novel strategy for exploring the effects of disease on sets of genes. We identified several modules that exhibited highly significant differences in *cor*(*KC*) between CTRL and HD subjects in CN. One potential drawback of our approach is that relatively small differences in *cor*(*KC*) can appear significant as |*cor*(*KC*)| approaches 1; for example, M34 was significant despite a relatively small difference between CTRL (*cor*(*KC*) = −0.98) and HD (*cor*(*KC*) = −0.91) subjects. For the four most significant modules, however, the differences in *cor*(*KC*) were > 1, indicating that the standardized *C*(*k*) curve had flipped from negative (CTRL) to positive (HD).

As illustrated above, differences between standardized *C*(*k*) curves are not simply a proxy for differences in network density, but also relate to network size and heterogeneity. We have also observed that small numbers of samples that are highly discordant (i.e. severe outliers) can have a large impact on the standardized *C*(*k*) curve (M.C.O. and S.H., unpublished observations). Thus, the standardized *C*(*k*) curve is an aggregate measure, and one that may be used to complement existing strategies for conducting both unsupervised and supervised analyses. We also note that in the present study, the overall relationship between differential expression (DE) and differences between the standardized *C*(*k*) curves of CTRL and HD subjects was weak. For example, although the salmon module (which exhibited the most significant difference in *cor*(*K*,*C*) between CTRL and HD) was strongly associated with DE, the red module (which also exhibited a significant difference in *cor*(*K*,*C*) between CTRL and HD) was not. Furthermore, our simulation study confirms that situations may exist in which *cor*(*K*,*C*) can distinguish meaningful sample subgroups in the absence of DE. These findings deserve additional study.

## Conclusions

As genomic technologies proliferate and genomic studies grow ever larger, it is critical that methods to assess sample heterogeneity evolve in parallel. We have presented a standardized approach for sample network analysis that can detect outlying samples in the absence of hierarchical clustering. We have also described a novel network statistic, *cor*(*K*,*C*), and demonstrated that it can be used to assess sample homogeneity, identify sample traits with global effects, and facilitate supervised comparisons among groups of samples using pre-selected subsets of features. Application of the latter approach to microarray data generated from human brain tissue identified a neuronal signal transduction module as an epicenter of transcriptional dysregulation in striatal samples from individuals with HD. To the best of our knowledge, these findings provide the first formal demonstration that network methods can distinguish biologically meaningful relationships among samples in genomic datasets. The dataset analyzed in this study, along with the SampleNetwork and ModuleSampleNetwork R functions and a comprehensive tutorial illustrating their usage, are available on our web site (http://www.genetics.ucla.edu/labs/horvath/CoexpressionNetwork/SampleNetwork).

## Methods

### R software implementation

We have implemented the sample network approach in a freely available, custom R software function called SampleNetwork. SampleNetwork has been designed to facilitate detailed exploration of sample relationships and expedite genomic data pre-processing decisions via sample network analysis. SampleNetwork enables semi-automatic, interactive sample network construction and network concept calculations. Network concepts include node-based measures such as the standardized sample connectivity (*Z*.*K*) and the standardized sample clustering coefficient (*Z*.*C*), as well as network-based measures such as *cor*(*KC*) and the mean inter-sample adjacency (*ISA*, or *density*). These concepts and many others are defined below and in Supplementary Methods (Additional file 1). By calculating the distributions of node-based sample network concepts, SampleNetwork enables the user to identify and remove outlying samples in an iterative and interactive fashion; by calculating network-based sample network concepts, SampleNetwork enables the user to gauge overall progress towards data cleanliness and sample homogeneity. These features are described in detail in our online tutorial (see below and Additional file 3). SampleNetwork also enables significance testing of sample covariates with respect to sample metrics, and data normalization. Data normalization may be performed pursuant to outlier removal using the quantile normalization method proposed in ref. [43].

Because sample networks often reveal groupings of samples that reflect batch effects (technical variation), which are typically not removed by standard normalization procedures, we have also incorporated existing methods that allow the user to automatically correct for batch effects. Specifically, we have found that the R function ComBat created by Johnson and colleagues [36] is quite adept at removing batch effects. Consequently, if batch effects are present, the user has the option of correcting for them by calling ComBat from within SampleNetwork, which automates its execution. SampleNetwork also requires installation of the following R (http://www.r-project.org/) and Bioconductor (http://www.bioconductor.org/) packages: affy [44], cluster, impute [45], preprocessCore, and WGCNA [34]. With each successive round of data processing, SampleNetwork produces and exports the results of sample network analysis automatically (e.g. Figure S1; Additional file 1).

We have also created a companion R software function called ModuleSampleNetwork to explore the properties of sample networks when formed over subsets of features. In our application, subsets of features correspond to modules of co-expressed genes [21], but we note that subsets can be defined by the user according to any criteria. ModuleSampleNetwork does not enable outlier testing and removal or data normalization, but instead seeks to compare module sample network properties between subgroups of samples (e.g. Figure 6) and across modules (e.g. Figure 4). An example workflow would involve using SampleNetwork to pre-process a microarray dataset, then using WGCNA [34] to identify modules of co-expressed genes, and finally using ModuleSampleNetwork to explore sample network properties at the modular level.

While both SampleNetwork and ModuleSampleNetwork are user-friendly, they are interactive and require judicious feedback from the user (for example, regarding thresholds for outlier removal). To illustrate how the software can be used in practice, we provide a detailed, annotated tutorial with R code (Additional file 3) highlighting the required input files, parameter choices, user interactions, and resulting output files. The beneficial effects of outlier detection and removal, data normalization, and correction for batch effects, as implemented using SampleNetwork, are clearly delineated by significance testing of sample covariates with respect to sample metrics, analysis of differential expression, and analysis of network concepts with each successive round of data processing, as described in the online tutorial. This tutorial, (Additional file 3) along with the required input files and the SampleNetwork and ModuleSampleNetwork R functions, is available on our web site (http://www.genetics.ucla.edu/labs/horvath/CoexpressionNetwork/SampleNetwork).

### Microarray data pre-processing

Raw microarray data (.CEL files) [14] were downloaded from Gene Expression Omnibus (http://www.ncbi.nlm.nih.gov/geo/query/acc.cgi?acc=GSE3790). Detailed information on sample characteristics and sample processing can be found in [14]. A summary of sample characteristics can also be found in Additional file 2. To eliminate non-specific and mis-targeted probes prior to generating expression values, a mask file (“HG-U133A”) was obtained from http://masker.nci.nih.gov/ev/[46] and applied to the raw microarray data using the R (http://www.r-project.org/) package “ProbeFilter” [47] (http://arrayanalysis.mbni.med.umich.edu/MBNIUM.html#ProbeFilter). After applying the mask file, only probe sets with at least seven remaining probes were retained for further analysis (n = 18,631). Expression values were generated in R using the “expresso” function of the “affy” package (http://www.bioconductor.org/) [48] with “mas” settings and no normalization, followed by scaling of arrays to the same average intensity (200).

### Sample networks based on general similarity or dissimilarity measures

The input of most clustering procedures is a similarity or dissimilarity measure. In Additional file 1, we define these measures and describe general approaches for turning a similarity or dissimilarity matrix into a sample network.

### Defining sample adjacency

To construct sample networks, a measure of connection strength, or *adjacency*, is defined for each pair of samples *i* and *j* and denoted by *a*_*ij*_. A mathematical constraint on *a*_*ij*_ is that its values must lie between 0 and 1. In our implementation, we defined the adjacency between (microarray) samples *S*_*i*_ and *S*_*j*_ as follows:

(1) $$a_{\text{ij}}={\left(\frac{cor\left(S_{i},S_{j}\right)+1}{2}\right)}^{\beta }$$

where β = 2. Technically, *a*_*ij*_ is a signed weighted adjacency matrix [22,49]. A major advantage of defining a network adjacency measure (as opposed to a general similarity measure) between samples is that it allows specification of network concepts (see below). Our proposed sample adjacency measure (based on β = 2) also has several other advantages. First, it preserves the sign of the correlation (although in most applications negative correlations among samples are unlikely to occur). Second, it preserves the continuous nature of the correlation information; alternative approaches based on thresholding the correlation coefficient may lead to information loss. Third, while any other power β could be used, the choice of β = 2 results in an adjacency measure that is close to the correlation when the correlation is large (e.g. larger than 0.6, which is often the case among samples in microarray data).

We note that SampleNetwork also allows the user to define sample adjacencies using Euclidean distance, which may be desirable in some applications. Future efforts may seek to compare the properties of sample networks using these and other adjacency measures.

### Network concepts

After constructing an adjacency matrix, nodes (samples) can be characterized in terms of a number of existing network concepts (see refs. [10,12] for comprehensive overviews of network concepts). Several of these concepts are reviewed briefly below, including the connectivity (also known as *degree* in unweighted networks) and the clustering coefficient, which we find to be particularly useful in the context of sample networks.

#### Connectivity

The *connectivity* (*k*) of the *i*-th network node is defined by:

(2) $$k_{i}=\sum_{j\neq i}a_{\mathrm{ij}}\text{.}$$

The *maximum connectivity* is defined as:

(3) $$k_{\max}=\max_{j}\left(k_{j}\right)\text{.}$$

The *scaled connectivity K*_i_ of the *i*-th network node is defined as:

(4) $$K_{i}=\frac{k_{i}}{k_{\max}}\text{.}$$

The *standardized connectivity Z.K*_i_ of the *i*-th network node is defined as:

(5) $$Z.K_{i}=\frac{K_{i}-\text{mean}\left(K\right)}{\sqrt{\mathrm{var}\left(K\right)}}$$

*Sample network interpretation of the connectivity*: Using our proposed measure of sample adjacency (signed weighted network with β = 2), we find that

(6) $$k_{i}\approx \sum_{i\neq j}cor(S_{i},S_{j})$$

if all sample correlations are > 0.6. In other words, samples with high connectivity tend to be highly positively correlated with other samples. The connectivity is the most widely used concept for distinguishing the nodes of a network. As illustrated in the motivational example above and as detailed in our R tutorial (Additional file 3), samples with low connectivity may represent outliers.

#### Clustering coefficient

The *clustering coefficient* (*C*) of node *i* measures the density of local connections, or “cliquishness” [11]. For weighted networks, 0 ≤ *a*_*ij*_ ≤ 1 implies that 0 ≤ *C*_*i*_ ≤ 1 [22]:

(7) $$C_{i}=\frac{\sum_{l\neq i}\sum_{m\neq i,l}a_{\mathrm{il}}a_{\mathrm{lm}}a_{\mathrm{mi}}}{\left\{{\left(\sum_{l\neq i}a_{\mathrm{il}}\right)}^{2}-{\sum_{l\neq i}\left(a_{\mathrm{il}}\right)}^{2}\right\}}\text{.}$$

The *standardized clustering coefficient Z.C*_i_ of the *i*-th network node is defined as:

(8) $$Z.C_{i}=\frac{C_{i}-\text{mean}\left(C\right)}{\sqrt{\mathrm{var}\left(C\right)}}$$

*Sample network interpretation of the clustering coefficient*: The higher the clustering coefficient of a sample, the higher is the average pairwise correlation among its closest neighbors. If all of a sample’s closest neighbors have pairwise correlations of −1, the clustering coefficient will be zero.

#### Density and mean intersample adjacency (ISA)

We find it useful to characterize sample networks using the mean (off-diagonal) adjacency measure, i.e.

(9) $$mean\left(A\right)=\frac{\sum_{i}\sum_{j\neq i}a_{\mathrm{ij}}}{n\left(n-1\right)}$$

where *A* = [*a*_*ij*_]. The mean adjacency is also known as the *density* of the network. In sample networks, we often refer to the density as the mean *intersample adjacency* (ISA).

*Sample network interpretation of the density*: Using our proposed measure of sample adjacency (signed weighted network with β = 2), we find that

(10) $$mean\left(A\right)\approx \frac{\sum_{i}\sum_{i\neq j}cor\left(S_{i},S_{j}\right)}{n\left(n-1\right)}$$

if all sample correlations are > 0.6. Thus, the mean adjacency is roughly equal to the mean correlation in sample networks.

### The standardized *C*(*k*) curve and *cor*(*K*,*C*) network concept

Empirical results obtained through application of the SampleNetwork R function to many datasets indicated that as outlying samples are removed, data are normalized, and technical artifacts (e.g. batch effects) are corrected, *Z*.*K* and *Z*.*C* exhibit a progressively linear, inverse relationship. A similar relationship has been observed in unweighted (binary) networks, where the relationship between the (unstandardized) connectivity and (unstandardized) clustering coefficient of network nodes, i.e. the *C*(*k*) curve, has previously been reported to follow a scaling law (*C* ≅ *k*^α^[29,31]), with values approaching −1 often observed for the scaling exponent α in biological systems [30,32]. It has been suggested that this relationship may emerge as a consequence of hierarchically modular networks, where nodes with low connectivity form small, densely connected clusters, and nodes with high connectivity serve to bridge these many small clusters into one large, integrated network [31].

We define the standardized *C*(*k*) curve as a scatter plot between *Z.K* and *Z.C* where *Z.K* and *Z.C* denote the standardized sample connectivity and the standardized sample clustering coefficient, respectively. We also introduce a new network concept, *cor*(*K*,*C*), which we define as the Spearman correlation between *Z*.*K* and *Z*.*C*. We note that other measures of correlation could also be used (e.g. Pearson correlation). Since the Spearman correlation is invariant with respect to a monotonically increasing transformation (e.g. standardization), we find that

(11) $$cor\left(K,C\right)=cor\left(Z.K,Z.C\right)=cor\left(k,C\right)\text{,}$$

where *k* denotes the unscaled connectivity. As described in Results, we find that *cor*(*K*,*C*) is inversely related to the density (i.e. mean adjacency) in simulated networks. However, because *cor*(*K*,*C*) is invariant if one scales all off-diagonal adjacencies by a constant, it is more accurate to consider *cor*(*K*,*C*) as an indicator of network heterogeneity. The network concept *Heterogeneity* is defined as:

(12) $$Heterogeneity=\frac{\sqrt{var\left(k\right)}}{mean\left(k\right)}$$

Let us briefly consider the special case of an exactly factorizable network in which the network adjacency factors into node-specific contributions (*a*_*ij*_ = CF(*i*) CF(*j*)) [10,50]. In this case, we have shown that the Spearman correlation *cor*(*KC*) is actually determined by the network heterogeneity:

(13) $$cor\left(K,C\right)\approx 0.96-2.19\frac{{\left(\sum_{i}k_{i}\right)}^{2}}{n\sum_{i}k_{i}^{2}}=0.96-2.19\frac{1}{1+Heterogeneity^{2}}\text{.}$$

Thus, *cor*(*KC*) close to 1 indicates that network heterogeneity is high. Divergence of *cor*(*KC*) from 1 (in a negative direction) implies increasing homogeneity; once a critical level of homogeneity in the network is breached (analogous to a percolation transition [33]), *cor*(*KC*) becomes negative. In practice, however, the relationship described above does not generalize to non-factorizable networks. In our real data applications that involve non-factorizable networks, *cor*(*KC*) also exhibits a dependence on the network size *n*.

#### Identification of significant differences between *cor*(*K*,*C*)

Differences in standardized *C*(*k*) curves may distinguish biologically interesting groups of samples. For example, assume two sample networks (corresponding to two groups of samples) and two corresponding measures of *cor*(*K*,*C*). To identify significant differences in *cor*(*K*,*C*) between two sample networks, we use a test for assessing the significance of differences in correlations from samples of different sizes. First, *cor*(*K*,*C*) for each sample group is transformed using the Fisher transformation:

(14) z=k0.5*log1+corK,Ck1−corK,Ck

where *k* indexes the sample networks being compared. For the comparison between groups (sample networks) 1 and 2, the difference between the resulting *z*-scores is divided by the joint standard error:

(15) $$z_{\mathrm{diff}}=\frac{z_{1}-z_{2}}{\sqrt{\frac{1}{\left(n_{1}-3\right)}+\frac{1}{\left(n_{2}-3\right)}}}$$

where *n*_1_ and *n*_2_ represent the number of samples in groups 1 and 2, respectively. Under the null hypothesis of equal *cor*(*K*,*C*), *z*_*diff*_ follows asymptotically a normal distribution (under weak assumptions). Therefore we calculate significance levels (*P*-values) for *z*_*diff*_ based upon the standard normal distribution.

### Simulation model for illustrating the ability of *cor*(*K*,*C*) to distinguish sample groups in the absence of differential expression

To further illustrate the utility of *cor*(*KC*), we simulated a set of 500 genes (referred to as a “module”) with the following properties: i) the first principal component (the observed module eigengene [ME]) exhibited no relationship to a simulated sample trait (referred to as “disease status”), and ii) *cor*(*KC*) distinguished “control” subjects from those with “moderate” or “severe” disease status. The module was simulated to contain two unrelated sub-modules of 200 and 300 genes, respectively. The first sub-module contained a signal for the simulated sample trait, while the second sub-module contained noise genes with no relation to disease status. The first sub-module was simulated in two steps. First, we used a seed ME as input for the simulateModule function from the R package WGCNA [34]. This function simulates genes with varying correlations around the seed ME and exports standardized gene expression values (i.e. each gene has mean = 0 and variance = 1). Second, we added a mean value to each module gene. Importantly, the mean gene expression values depended on the value of the seed ME. For subjects whose seed ME values were above the median, mean expression values were drawn from a normal distribution with mean = 2 and standard deviation = 2. For subjects whose seed ME values were below the median, mean expression values were 2/3 those of the control subjects (i.e. it was assumed that the disease lowered the mean gene expression values in sub-module 1). Analogously, we simulated the expression values for the second sub-module. However, we assumed that the mean gene expression values were derived from a normal distribution with mean = 2/3 and standard deviation = 2/3 (i.e. the mean values of these genes tended to have lower expression values than those of the first sub-module). The sample trait was simulated by thresholding the seed ME of the first sub-module. We assumed that healthy control subjects have a high value of the seed ME. Specifically, we simulated 100 individuals, with 50 designated as “control” subjects (darkgreen), 25 designated as “moderate” disease status (red), and 25 designated as “severe” disease status (turquoise), as indicated in Figure 5. In practice, the seed ME was not known. Instead, the observed ME for the entire module was obtained as the first principal component of the set of 500 genes.

### Additional network concepts for sample networks

In addition to characterizing sample networks via the connectivity and the clustering coefficient, it is also possible to characterize sample networks using additional network concepts. Such concepts include *decentralization* and *homogeneity*, as well as summaries of node-based measures such as the mean correlation, mean connectivity, mean clustering coefficient, mean intersample adjacency (or density), and mean *maximum adjacency ratio* (*MAR*). When applied to sample networks, these concepts provide a battery of measures for comparing the consistency of sample behavior within and across datasets. These network concepts are calculated automatically by SampleNetwork and are discussed further in Additional file 1 and our R tutorial (Additional file 3).

### Differential expression analysis

To determine whether specific CN gene co-expression modules were associated with DE in HD, for each CN module we calculated the ME (i.e. the first principal component obtained by singular value decomposition), which is a vector that summarizes the characteristic expression pattern of a module [10]. We then used Student’s *t*-test to determine whether the mean expression levels of the ME differed between groups (distinguished by HD diagnosis). An advantage of this approach is that the extent of modular DE can be summarized by a single *P*-value. Future efforts may seek to incorporate higher-order representative features (beyond the first principal component) to explore additional relationships between gene co-expression modules and disease status [51]. Differential gene expression in CN between CTRL and HD subjects (Additional file 4) was assessed using Student’s *t*-test on log_2_-transformed expression values. The resulting *P*-values were corrected for multiple comparisons by controlling for the false-discovery rate [52]. The resulting local false-discovery rates (referred to as *Q*-values), along with mean expression levels for CTRL and HD, are reported for all genes in the salmon module in Additional file 4.

### Ingenuity pathways analysis

Ingenuity Pathways Analysis (IPA; http://www.ingenuity.com/) was used to determine whether gene co-expression modules identified in [21] were enriched with functional interactions among their constituent genes. For each module, probe sets that were positively correlated with the module eigengene (*P* < 0.001) were used to search for enrichment. Network construction was restricted to experimentally verified, direct physical interactions. IPA reported false-discovery rate (FDR)-corrected *P*-values for the 500 most enriched functionally annotated categories of genes in each module. Results for the salmon module are reported in Additional file 5.

## Abbreviations

Adj, adjacency; BA4, Brodmann’s area 4 (primary motor cortex); BA9, Brodmann’s area 9 (prefrontal cortex); CB, cerebellum; CN, caudate nucleus; cor(K,C), the Spearman correlation between the standardized connectivity and the standardized clustering coefficient; CTRL, control; DE, differential expression; Dx, diagnosis; HD, Huntington’s disease; IPA, Ingenuity Pathways Analysis; ISA, intersample adjacency; p.DE, P-value for the significance of differential expression; p.Diff.cor(K,C), P-value for the significance of differences between cor(K,C); WGCNA, weighted gene co-expression network analysis; Z.K, the standardized connectivity; Z.C, the standardized clustering coefficient.

## Competing interests

The authors declare that they have no competing interests.

## Authors’ contributions

MCO and SH designed the SampleNetwork and ModuleSampleNetwork R functions and drafted the manuscript. MCO created the SampleNetwork and ModuleSampleNetwork R functions and the R tutorial; MCO performed the data analysis. PL performed a simulation study (Figure 5) and created the web site. All authors read and approved the final manuscript.

## Supplementary Material

Additional file 1 **Supplementary information.** PDF file containing Supplementary Methods, Supplementary References, and Supplementary Figures (1–8). Click here for file

Additional file 2 **Sample information.** XLS table that summarizes sample information, including Gene Expression Omnibus (GEO: http://www.ncbi.nlm.nih.gov/geo/) sample ID, sample labels, diagnosis, severity grade, age, sex, individual ID, hybridization date, hybridization batch assignment, and country of processing, as described in ref. [14]. Click here for file

Additional file 3 **SampleNetwork R tutorial.** DOC file containing annotated R code and detailed instructions for executing the SampleNetwork and ModuleSampleNetwork R functions. The datasets that are referenced in the tutorial and analyzed in this study can be downloaded from: http://www.genetics.ucla.edu/labs/horvath/CoexpressionNetwork/SampleNetwork. Click here for file

Additional file 4 **Summary of differential expression, functional enrichment, and module membership for genes in the salmon module.** XLS table that summarizes the extent of differential expression, functional enrichment, and membership strength for the salmon module. Differential expression analyses include CTRL vs. HD human caudate nucleus samples [14], CTRL vs. HD human laser-microdissected striatal neurons [14], and wild-type mice vs. a mutant mouse model of HD cultured primary striatal neurons [20]. Functional enrichment categories included G-protein coupled receptors, phosphatidylinositol signaling, calmodulin binding, ion transport, and calcium ion binding; all of these categories were significantly enriched in the salmon module [21]. Module membership values and corresponding *P*-values are taken from ref. [21]. Click here for file

Additional file 5 **Ingenuity Pathways Analysis of salmon module genes.** XLS table that reports false-discovery rate (FDR)-corrected *P*-values for the 500 most enriched functionally annotated categories of genes in the salmon module from Ingenuity Pathways Analysis (IPA; http://www.ingenuity.com/). Click here for file

## References

[B1] Nugent R Meila M An overview of clustering applied to molecular biology Methods Mol Biol 2010 620 369 404 10.1007/978-1-60761-580-4_12 20652512

[B2] Carugo O Clustering criteria and algorithms Methods Mol Biol 2010 609 175 196 10.1007/978-1-60327-241-4_11 20221920

[B3] Carugo O Proximity measures for cluster analysis Methods Mol Biol 2010 609 163 174 10.1007/978-1-60327-241-4_10 20221919

[B4] Frades I Matthiesen R Overview on techniques in cluster analysis Methods Mol Biol 2010 593 81 107 10.1007/978-1-60327-194-3_5 19957146

[B5] Kerr G Ruskin HJ Crane M Doolan P Techniques for clustering gene expression data Comput Biol Med 2008 38 3 283 293 10.1016/j.compbiomed.2007.11.001 18061589

[B6] Shannon W Culverhouse R Duncan J Analyzing microarray data using cluster analysis Pharmacogenomics 2003 4 1 41 52 10.1517/phgs.4.1.41.22581 12517285

[B7] Gower JC Legendre P Metric and Euclidean properties of dissimilarity coefficients J Classif 1986 3 1 5 48 10.1007/BF01896809

[B8] Schaid DJ Genomic similarity and kernel methods II: methods for genomic information Hum Hered 2010 70 2 132 140 10.1159/000312643 20606458 PMC7077756

[B9] Schaid DJ Genomic similarity and kernel methods I: advancements by building on mathematical and statistical foundations Hum Hered 2010 70 2 10.1159/000312641 PMC7077093 20610906

[B10] Horvath S Dong J Geometric interpretation of gene coexpression network analysis PLoS Comput Biol 2008 4 8 e1000117 10.1371/journal.pcbi.1000117 18704157 PMC2446438

[B11] Watts DJ Strogatz SH Collective dynamics of ‘small-world’ networks Nature 1998 393 6684 440 442 10.1038/30918 9623998

[B12] Almaas E Biological impacts and context of network theory J Exp Biol 2007 210 Pt 9 1548 1558 17449819 10.1242/jeb.003731

[B13] Horvath S Weighted network analysis. Applications in genomics and systems biology 2011 New York: Springer Book

[B14] Hodges A Strand AD Aragaki AK Kuhn A Sengstag T Hughes G Elliston LA Hartog C Goldstein DR Thu D Regional and cellular gene expression changes in human Huntington’s disease brain Hum Mol Genet 2006 15 6 965 977 10.1093/hmg/ddl013 16467349

[B15] Ross CA Margolis RL Huntington’s disease Clin Neurosci Res 2001 1 1–2 142 152

[B16] Group HDCR A novel gene containing a trinucleotide repeat that is expanded and unstable on Huntington’s disease chromosomes. The Huntington’s Disease Collaborative Research Group Cell 1993 72 6 971 983 10.1016/0092-8674(93)90585-E 8458085

[B17] Cha JH Transcriptional signatures in Huntington’s disease Prog Neurobiol 2007 83 4 228 248 10.1016/j.pneurobio.2007.03.004 17467140 PMC2449822

[B18] Cha JH Kosinski CM Kerner JA Alsdorf SA Mangiarini L Davies SW Penney JB Bates GP Young AB Altered brain neurotransmitter receptors in transgenic mice expressing a portion of an abnormal human huntington disease gene Proc Natl Acad Sci U S A 1998 95 11 6480 6485 10.1073/pnas.95.11.6480 9600992 PMC27817

[B19] Luthi-Carter R Strand A Peters NL Solano SM Hollingsworth ZR Menon AS Frey AS Spektor BS Penney EB Schilling G Decreased expression of striatal signaling genes in a mouse model of Huntington’s disease Hum Mol Genet 2000 9 9 1259 1271 10.1093/hmg/9.9.1259 10814708

[B20] Runne H Regulier E Kuhn A Zala D Gokce O Perrin V Sick B Aebischer P Deglon N Luthi-Carter R Dysregulation of gene expression in primary neuron models of Huntington’s disease shows that polyglutamine-related effects on the striatal transcriptome may not be dependent on brain circuitry J Neurosci 2008 28 39 9723 9731 10.1523/JNEUROSCI.3044-08.2008 18815258 PMC6671220

[B21] Oldham MC Konopka G Iwamoto K Langfelder P Kato T Horvath S Geschwind DH Functional organization of the transcriptome in human brain Nat Neurosci 2008 11 11 1271 1282 10.1038/nn.2207 18849986 PMC2756411

[B22] Zhang B Horvath S A general framework for weighted gene co-expression network analysis Stat Appl Genet Mol Biol 2005 4 1 Article 17 10.2202/1544-6115.1128 16646834

[B23] Vonsattel JP Myers RH Stevens TJ Ferrante RJ Bird ED Richardson EP Neuropathological classification of Huntington’s disease J Neuropathol Exp Neurol 1985 44 6 559 577 10.1097/00005072-198511000-00003 2932539

[B24] Xu M Li W James GM Mehan MR Zhou XJ Automated multidimensional phenotypic profiling using large public microarray repositories Proc Natl Acad Sci U S A 2009 106 30 12323 12328 10.1073/pnas.0900883106 19590007 PMC2708172

[B25] Ernst C Sequeira A Klempan T Ernst N Ffrench-Mullen J Turecki G Confirmation of region-specific patterns of gene expression in the human brain Neurogenetics 2007 8 3 219 224 10.1007/s10048-007-0084-2 17375343

[B26] Khaitovich P Muetzel B She X Lachmann M Hellmann I Dietzsch J Steigele S Do HH Weiss G Enard W Regional patterns of gene expression in human and chimpanzee brains Genome Res 2004 14 8 1462 1473 10.1101/gr.2538704 15289471 PMC509255

[B27] Oldham MC Horvath S Geschwind DH Conservation and evolution of gene coexpression networks in human and chimpanzee brains Proc Natl Acad Sci U S A 2006 103 47 17973 8 10.1073/pnas.0605938103 17101986 PMC1693857

[B28] Roth RB Hevezi P Lee J Willhite D Lechner SM Foster AC Zlotnik A Gene expression analyses reveal molecular relationships among 20 regions of the human CNS Neurogenetics 2006 7 2 67 80 10.1007/s10048-006-0032-6 16572319

[B29] Dorogovtsev SN Goltsev AV Mendes JF Pseudofractal scale-free web Phys Rev E Stat Nonlin Soft Matter Phys 2002 65 6 Pt 2 066122 12188798 10.1103/PhysRevE.65.066122

[B30] Ravasz E Detecting hierarchical modularity in biological networks Methods Mol Biol 2009 541 145 160 10.1007/978-1-59745-243-4_7 19381526

[B31] Ravasz E Barabasi AL Hierarchical organization in complex networks Phys Rev E Stat Nonlin Soft Matter Phys 2003 67 2 Pt 2 026112 12636753 10.1103/PhysRevE.67.026112

[B32] Ravasz E Somera AL Mongru DA Oltvai ZN Barabasi AL Hierarchical organization of modularity in metabolic networks Science 2002 297 5586 1551 1555 10.1126/science.1073374 12202830

[B33] Vazquez A Dobrin R Sergi D Eckmann JP Oltvai ZN Barabasi AL The topological relationship between the large-scale attributes and local interaction patterns of complex networks Proc Natl Acad Sci U S A 2004 101 52 17940 17945 10.1073/pnas.0406024101 15598746 PMC539752

[B34] Langfelder P Horvath S WGCNA: an R package for weighted correlation network analysis BMC Bioinforma 2008 9 559 10.1186/1471-2105-9-559 PMC2631488 19114008

[B35] Iancu OD Darakjian P Walter NA Malmanger B Oberbeck D Belknap J McWeeney S Hitzemann R Genetic diversity and striatal gene networks: focus on the heterogeneous stock-collaborative cross (HS-CC) mouse BMC Genomics 2010 11 585 10.1186/1471-2164-11-585 20959017 PMC3091732

[B36] Johnson WE Li C Rabinovic A Adjusting batch effects in microarray expression data using empirical Bayes methods Biostatistics 2007 8 1 118 127 10.1093/biostatistics/kxj037 16632515

[B37] Sun J Zhao Z A comparative study of cancer proteins in the human protein-protein interaction network BMC Genomics 2010 11 Suppl 3 S5 10.1186/1471-2164-11-S3-S5 PMC2999350 21143787

[B38] Shen-Orr SS Milo R Mangan S Alon U Network motifs in the transcriptional regulation network of Escherichia coli Nat Genet 2002 31 1 64 68 10.1038/ng881 11967538

[B39] Milo R Shen-Orr S Itzkovitz S Kashtan N Chklovskii D Alon U Network motifs: simple building blocks of complex networks Science 2002 298 5594 824 827 10.1126/science.298.5594.824 12399590

[B40] Onnela JP Saramaki J Kertesz J Kaski K Intensity and coherence of motifs in weighted complex networks Phys Rev E Stat Nonlin Soft Matter Phys 2005 71 6 Pt 2 065103 16089800 10.1103/PhysRevE.71.065103

[B41] Kuhn A Goldstein DR Hodges A Strand AD Sengstag T Kooperberg C Becanovic K Pouladi MA Sathasivam K Cha JH Mutant huntingtin’s effects on striatal gene expression in mice recapitulate changes observed in human Huntington’s disease brain and do not differ with mutant huntingtin length or wild-type huntingtin dosage Hum Mol Genet 2007 16 15 1845 1861 10.1093/hmg/ddm133 17519223

[B42] de la Fuente A From ‘differential expression’ to ‘differential networking’ - identification of dysfunctional regulatory networks in diseases Trends Genet 2010 26 7 326 333 10.1016/j.tig.2010.05.001 20570387

[B43] Bolstad BM Irizarry RA Astrand M Speed TP A comparison of normalization methods for high density oligonucleotide array data based on variance and bias Bioinformatics 2003 19 2 185 193 10.1093/bioinformatics/19.2.185 12538238

[B44] Gautier L Cope L Bolstad BM Irizarry RA affy–analysis of Affymetrix GeneChip data at the probe level Bioinformatics 2004 20 3 307 315 10.1093/bioinformatics/btg405 14960456

[B45] Troyanskaya O Cantor M Sherlock G Brown P Hastie T Tibshirani R Botstein D Altman RB Missing value estimation methods for DNA microarrays Bioinformatics 2001 17 6 520 525 10.1093/bioinformatics/17.6.520 11395428

[B46] Zhang J Finney RP Clifford RJ Derr LK Buetow KH Detecting false expression signals in high-density oligonucleotide arrays by an in silico approach Genomics 2005 85 3 297 308 10.1016/j.ygeno.2004.11.004 15718097

[B47] Dai M Wang P Boyd AD Kostov G Athey B Jones EG Bunney WE Myers RM Speed TP Akil H Evolving gene/transcript definitions significantly alter the interpretation of GeneChip data Nucleic Acids Res 2005 33 20 e175 10.1093/nar/gni179 16284200 PMC1283542

[B48] Gentleman RC Carey VJ Bates DM Bolstad B Dettling M Dudoit S Ellis B Gautier L Ge Y Gentry J Bioconductor: open software development for computational biology and bioinformatics Genome Biol 2004 5 10 R80 10.1186/gb-2004-5-10-r80 15461798 PMC545600

[B49] Mason MJ Fan G Plath K Zhou Q Horvath S Signed weighted gene co-expression network analysis of transcriptional regulation in murine embryonic stem cells BMC Genomics 2009 10 327 10.1186/1471-2164-10-327 19619308 PMC2727539

[B50] Dong J Horvath S Understanding network concepts in modules BMC Syst Biol 2007 1 24 10.1186/1752-0509-1-24 17547772 PMC3238286

[B51] Ma S Kosorok MR Huang J Dai Y Incorporating higher-order representative features improves prediction in network-based cancer prognosis analysis BMC Med Genomics 2011 4 5 10.1186/1755-8794-4-5 21226928 PMC3037289

[B52] Benjamini Y Hochberg Y Controlling the false discovery rate: a practical and powerful approach to multiple testing J Roy Statist Soc Ser B 1995 57 1 289 300

