# Supplementary material for: Network methods for describing sample relationships in genomic datasets: application to Huntington’s disease
Source: BMC Syst Biol. 2012 Jun 12;6:63. doi: 10.1186/1752-0509-6-63 (PMC3441531; doi:10.1186/1752-0509-6-63)
Supplement: Additional file 1 — Supplementary information. PDF file containing Supplementary Methods, Supplementary References, and Supplementary Figures (1–8). [file 1752-0509-6-63-S1.pdf]

SUPPLEMENTARY INFORMATION

Network methods for describing sample relationships in genomic datasets: application to Huntington's disease

Michael C. Oldham, Peter Langfelder, and Steve Horvath

Correspondence: oldhamm@stemcell.ucsf.edu and shorvath@mednet.ucla.edu

Table of Contents

1. Introduction

2. Supplementary Methods

2.1. Sample networks based on general similarity or dissimilarity measures
    2.1.1. Turning a similarity or dissimilarity matrix into a network
2.2. Exploring the relationship between correlation networks and Euclidean distance-based networks
2.3. Additional network concepts
    2.3.1. Maximum adjacency ratio (MAR)
    2.3.2. Decentralization
    2.3.3. Homogeneity
2.4. Calculating module eigengenes
2.5. Relating sample metrics to sample traits
2.6. Normalizing and correcting for batch effects

3. Supplementary References

4. Supplementary Figures

Supplementary Figure 1 | Sample networks provide a novel perspective on Huntington's disease.
Supplementary Figure 2 | Connectivity is positively correlated with the clustering coefficient in modular and random gene expression networks.
Supplementary Figure 3 | Sample adjacencies are degraded in caudate nucleus relative to other brain regions.
Supplementary Figure 4 | Caudate nucleus samples exhibit significant segregation by diagnosis in gene co-expression module M11C (black).
Supplementary Figure 5 | Caudate nucleus samples exhibit significant segregation by diagnosis in gene co-expression module M36 (royalblue).

Supplementary Figure 6 | Caudate nucleus samples exhibit significant segregation by diagnosis in gene co-expression module M19C (red).
Supplementary Figure 7 | Module enrichment analysis of differentially expressed genes in an *in vitro* model of Huntington's disease.
Supplementary Figure 8 | *cor*(*K*,*C*) exhibits similar behavior in unweighted networks.

1. Introduction

Below we provide further information on sample network construction using different similarity measures, additional network concepts, and other supplementary methods. We also describe how to calculate module eigengenes, relate sample network metrics to sample traits, and provide a brief discussion of normalization methods, including correction for batch effects. Eight supplementary figures are presented. We have also created a detailed tutorial illustrating usage of the SampleNetwork and ModuleSampleNetwork R functions, which highlights required input files and formats, parameter choices, user interactions, and output files. This tutorial, along with the SampleNetwork and ModuleSampleNetwork R functions, and the required input files, is available on our web site: http://www.genetics.ucla.edu/labs/horvath/CoexpressionNetwork/SampleNetwork.

2. Supplementary Methods

2.1. Sample networks based on general similarity or dissimilarity measures

Just as cluster analysis can take as input a variety of similarity or dissimilarity measures (correlation, Euclidean distance, mutual information, etc.), any such measure can be used to construct sample networks.

2.1.1. Turning a similarity or dissimilarity matrix into a network

A similarity matrix (also referred to as a similarity measure) is defined as an n x n dimensional, symmetric matrix $S = s_{ij}$ whose entries are non-negative numbers. Thus, the components of a similarity matrix must satisfy the following conditions:

$$s_{ij} >= 0 \text{ (non-negativity)},$$
$$s_{ij} = s_{ji} \text{ (symmetry)}.$$

A network adjacency matrix is a special case of a similarity matrix: it is a similarity matrix whose entries are less than or equal to 1 and whose diagonal elements are equal to 1. To construct a network adjacency matrix from a similarity matrix, one needs to transform the similarity matrix so that its off-diagonal elements lie between 0 and 1 and its diagonal elements equal 1. This can be achieved by finding upper bounds for the similarities. We call an n x n dimensional, symmetric matrix $UpperBounds_s = UpperBounds_{ij}$ a matrix of upper bounds for S if its elements satisfy the following conditions:

$$s_{ij} <= UpperBounds_{ij} \quad \text{(if } i \text{ is different from } j\text{)},$$
$$s_{ii} = UpperBounds_{ii} \quad \text{(for diagonal elements)},$$
$$UpperBounds_{ij} = UpperBounds_{ji} \quad \text{(symmetry)}.$$

The component-wise matrix division:

$$A = S \,/\, UpperBounds_s$$

is defined as the matrix whose *ij*-th element is given by s_{ij} / $UpperBounds_{ij}$. One can easily verify that the resulting A satisfies the properties of an adjacency matrix. As an example, consider the similarity matrix

$$S = |cov(datX)| = |cov(x_i,x_j)|$$

of absolute values of covariances between the numeric vectors x_i (i = 1,....,n). A well known inequality (the Cauchy-Schwarz inequality) can be used to show that

$$UpperBounds_s = sqrt(var(x_i)*var(x_j))$$

is a matrix of upper bounds for S. Then:

$$A = S / UpperBounds_s = |cor(x_i,x_j)|$$

is simply the absolute value of pairwise correlations, which satisfies the properties of an adjacency matrix.

In the following, we describe how to construct a network based on a dissimilarity matrix. A dissimilarity matrix is defined to be a symmetric matrix $D = D_{ij}$ of non-negative numbers with diagonal elements equal to 0, i.e. its elements satisfy the following conditions:

$$D_{ij} >= 0 \quad \text{(non-negativity)},$$
$$D_{ij} = D_{ji} \quad \text{(symmetry)},$$
$$D_{ii} = 0 \quad \text{(zero diagonal)}.$$

Any monotonically decreasing function can be used to turn a dissimilarity matrix into a similarity matrix:

$$S = DecreasingFunction(D) = DecreasingFunction(D_{ij}).$$

Next, the resulting similarity matrix can be used to define a network adjacency matrix as described above. For example, if $UpperBounds(D)$ denotes a symmetric matrix of element-wise upper bounds for D, then

$$A = 1 - D / UpperBound(D)$$

defines an adjacency matrix.

2.2. Exploring the relationship between correlation networks and Euclidean distance-based networks

While sample networks can be defined based on a general distance matrix, we typically use signed correlation networks to define sample networks. Here we argue that a signed correlation network is in a certain sense equivalent to a Euclidean distance-based network between scaled vectors. Recall that the Euclidean distance between two vectors is defined as follows:

$$|| S_i - S_j || = \sqrt{\sum_{u=1}^{m} (S_{iu} - S_{ju})^2}\,.$$

Consider the matrix of pairwise squared Euclidean distances $D = (||S_i - S_j||)^2$ between sample vectors. Denote by max$Diss$ = $max(D)$ the maximum squared distance between the vectors. One can then define a distance-based adjacency matrix as follows:

$$A_{ij} = \frac{|| S_i - S_j ||^2}{\max Diss}\,.$$

Consider the scale function that scales (standardizes) the components of a vector. In vector notation,

$$scale(S_i) = \frac{S_i - mean(S_i)}{\sqrt{\text{var}(S_i))}}\,.$$

For the Euclidean distance-based network between scaled vectors, we find that

$$A_{ij}^{Euclid} = \frac{\| scale(S_i) - scale(S_j) \|^2}{\max Diss} = \frac{cor(S_i, S_j) - \min Cor}{1 - \min Cor}$$

where min*Cor* denotes the minimum entry of the pairwise correlation matrix. This equation follows directly from the following relationship:

$$\left\| scale(S_i) - scale(S_j) \right\|^2 = 2(m-1)\left(1 - cor(S_i, S_j)\right),$$

where m equals the number of components of S_i.

Note that A_{ij}^{Euclid} is a monotonically increasing function of $cor(S_i,S_j)$. Thus, any signed correlation network (defined as an increasing function of $cor(S_i,S_j)$) is a monotonically increasing function of the Euclidean distance-based network between scaled vectors.

2.3. Additional network concepts

Sample networks can be characterized by a variety of concepts that describe properties of individual nodes (samples) or properties of a network as a whole. These concepts provide diagnostic measures for comparing the consistency of sample behavior within and across datasets. An advantage of using such measures is that they provide an unbiased approach for comparing the quality and variability of data generated by different studies, including data produced by different technology platforms. Several additional network concepts are described below.

2.3.1. Maximum Adjacency Ratio

For weighted networks, we define the maximum adjacency ratio (*MAR*) of node i as follows:

$$MAR_i = \frac{\sum_{j \neq i} \left(a_{ij}\right)^2}{\sum_{j \neq i} a_{ij}},$$

which is defined if $k_i = \sum_{j \neq i} a_{ij} > 0$. Note that $0 \leq a_{ij} \leq 1$ implies that $0 \leq MAR_i \leq 1$. If all non-zero adjacencies take on their maximum value, then $MAR_i = 1$ (hence the name "maximum adjacency ratio"). If all non-zero adjacencies take on a small, constant value $a_{ij} = \varepsilon$, then $MAR_i = \varepsilon$ will be small.

Sample network interpretation of the maximum adjacency ratio: $MAR_i = 1$ suggests that the i-th sample has extreme correlations with other samples (close to 1 or –1). $MAR_i = 0.5$ suggests that the i-th sample has moderate correlations (e.g. 0.4) with other samples. The *MAR* can help determine whether a highly connected "hub" sample has moderate correlations with many samples or very high positive correlations with relatively few samples. In weighted sample networks, we find that the *MAR* is often highly correlated with the connectivity k. But in other contexts, the *MAR* has sometimes (though not always) been found to be superior to k when it comes to identifying biologically important hubs [1].

2.3.2. Decentralization

The network *decentralization* is given by:

$$Decentralization = 1 - \frac{n}{n-2}\left(\frac{k_{\max}}{n-1} - Density\right)$$

where *Density*, or "intersample adjacency" (ISA), is defined as the mean adjacency formed across the off-diagonal node pairs. For a network with a star topology, the decentralization is 0; in contrast, the decentralization is 1 for a network in which each node has the same connectivity.

Sample network interpretation of the decentralization: The decentralization of the sample network is close to 0 if one sample is highly correlated with all others while the remaining samples have lower correlations with each other. A sample network with high decentralization consists of samples that are highly correlated with one another (as is often the case).

2.3.3. Homogeneity

The network *homogeneity* is defined as follows:

$$Homogeneity = 1 - \frac{\sqrt{var(k)}}{mean(k)}$$

The homogeneity is invariant if the connectivity is multiplied by a scalar. The homogeneity is always less than 1, but can take on negative values.
Sample network interpretation of the homogeneity: The homogeneity measures the variation of connectivity across samples. The homogeneity will be low if a few samples have high connectivity while most others have low connectivity. Thus, data with low homogeneity may contain outlying samples. As described in the journal article, in the special situation of an exactly factorizable network, we find that the network concept $cor(K,C)$ is determined by the network heterogeneity (i.e. 1 – *Homogeneity*).

2.4. Calculating module eigengenes

Assume an m x n dimensional matrix *datX* whose i-th column x_i is a numeric vector (e.g. gene expression levels) with m components (e.g. number of samples). Before carrying out the singular value decomposition (SVD), we typically scale the columns of *datX* so that they have mean = 0 and variance = 1. The SVD of *datX* is given by the matrix multiplication of three matrices:

$$datX = U\,D\,(V)^T$$

where T denotes the transpose. The m x $min(m,n)$ dimensional matrix U and the n x $min(m,n)$ dimensional matrix V contain orthonormal columns. The columns of the matrices U and V are referred to as left and right singular vectors, respectively. The matrices U and D are given by:

$$U = (u_1\ u_2 ...)$$

$$D = diag\{|d_1|,|d_2|,...\}.$$

The singular values are defined as diagonal elements $|d_1|,|d_2|,...$ of the diagonal matrix D. We use the absolute value sign around the singular values to indicate that the singular values are non-negative real numbers.

We assume that $|d_1|$ denotes the largest singular value. Often the first singular value $|d_1|$ is strictly larger than the other singular values. In this case, u_1 and v_1 are uniquely defined up to a sign. In practice, we fix the sign of u_1 by requiring that its average correlation with the columns of *datX* is positive. When the columns of *datX* refer to genes, u_1 is referred to as an eigengene. Furthermore, if the genes correspond to a gene module, then u_1 is referred to as a module eigengene.

2.5. Relating sample metrics to sample traits

In most genomic studies, there is often some available information that may be used to try to explain differences among samples (i.e. sample covariates or "sample traits"). For example, samples may possess different biological traits such as age, gender, tissue type, treatment type, etc. There may also be technical sources of variation that can contribute to differences among samples (e.g. processing date, ascertainment center, or batch effects). Given all of the known

biological and technical sources of variation that may exist within a dataset, it is useful to know which traits exert significant effects on measured activity (e.g. gene expression levels, protein abundance, etc). Global effects can be ascertained by fitting a model in which the outcome is summarized for each sample and regressed upon a linear combination of sample traits. There are many ways to summarize measured activity for a given sample, and three are currently implemented in the SampleNetwork R function: mean (i.e. the mean activity level), *Z.K* (i.e. the standardized sample connectivity), and pc1 (i.e. the first principal component obtained by singular value decomposition of the activity matrix). Note that the user may implement each of these summaries for all features or a user-defined subset of features. SampleNetwork automatically relates the chosen summary measure to the user-specified sample traits using Analysis of Variance (ANOVA) and univariate or multivariate regression models, as illustrated in Figure 6E from the journal article and in our online R tutorial, which is available at http://www.genetics.ucla.edu/labs/horvath/CoexpressionNetwork/SampleNetwork.

While it is useful to know whether a given source of variation exerts a significant global effect on measured activity, it is often the case that only certain "levels" of a categorical variable will exert a significant effect. For example, consider a microarray experiment in which samples were hybridized on six separate days over a span of many months. Imagine that on five of these days an experienced technician performed the experiments, but on the sixth day, an inexperienced new hire performed the experiments and inadvertently used more labeled target material. Because samples hybridized on this day constitute only a small fraction of the total number, hybridization batch may not appear to exert a significant effect on global gene expression when all batches are considered. However, if each batch is considered in isolation, then the effect may become very obvious. This functionality has been implemented in SampleNetwork such that individual levels of categorical variables may be screened for significant effects using ANOVA and univariate or multivariate regression models, as illustrated in Figure 6E from the journal article and in our online tutorial.

2.6. Normalizing and correcting for batch effects

Normalization refers to the process of adjusting measured activity to remove or reduce the impact of technical sources of variation. A large number of algorithms have been developed to accomplish this goal, many of which are specific to different technology platforms [2-4]. It is beyond the scope of the present work to provide a review of these methods, as it is beyond the scope of SampleNetwork to provide a complete menu of normalization options. At present, if the user wishes to use SampleNetwork for data normalization, quantile normalization [5] will be implemented. Quantile normalization imposes the same distribution of measured activity on every sample within the dataset, and is therefore an appropriate normalization method when there is good reason to believe that the true underlying distributions of activity levels among samples within a dataset should be very similar (such as when all samples are taken from the same tissue). It should be noted that SampleNetwork will also export an unnormalized matrix (with outlier samples removed) that can be used as input for other normalization functions, if desired.

Standard normalization methods, including quantile normalization, typically do not eliminate batch effects [6]. SampleNetwork will allow the user to perform additional normalization to remove batch effects if they are deemed to be present. Batch normalization is performed by calling an R function called ComBat [7], which we have found to be very effective in eliminating batch effects (see also ref. [6]). ComBat uses empirical Bayes methods to adjust

batch effects and has been implemented within SampleNetwork using its default options. To call ComBat from within SampleNetwork, the user must indicate which column in the sample information file provides the batch structure, as well as which columns (if any) in the sample information file should be included as biological covariates (see our online tutorial at http://www.genetics.ucla.edu/labs/horvath/CoexpressionNetwork/SampleNetwork and ref. [7]). Note that SampleNetwork enables the user to run ComBat independently of quantile normalization, and vice versa. If the user chooses to run ComBat, a sample information file required by ComBat will be automatically generated by SampleNetwork and exported to the /SampleNetwork subdirectory. In the event that only one level of a batch or a subset of batch levels is deemed significant, it is up to the user to decide how the batch normalization should be performed. Continuing with the example above, imagine that there is one batch among six that has a strong influence on gene expression. If the user were to employ SampleNetwork to correct only for this batch, then a total of two batches would be passed to ComBat (one for samples from the "bad" batch, and one for all of the other samples). Alternatively, the user could pass the entire batch structure to ComBat (i.e. all six), even though only one batch exerted a strong influence on activity levels. The relative merits of each approach deserve further study.

3. Supplementary References

1. Horvath S, Dong J: **Geometric interpretation of gene coexpression network analysis.** *PLoS Comput Biol* 2008, **4**(8):e1000117.
2. Autio R, Kilpinen S, Saarela M, Kallioniemi O, Hautaniemi S, Astola J: **Comparison of Affymetrix data normalization methods using 6,926 experiments across five array generations**. *BMC Bioinformatics* 2009, **10 Suppl 1**:S24.
3. Mecham BH, Nelson PS, Storey JD: **Supervised normalization of microarrays**. *Bioinformatics* 2010, **26**(10):1308-1315.
4. Schmid R, Baum P, Ittrich C, Fundel-Clemens K, Huber W, Brors B, Eils R, Weith A, Mennerich D, Quast K: **Comparison of normalization methods for Illumina BeadChip HumanHT-12 v3.** *BMC Genomics* 2010, **11**:349.
5. Bolstad BM, Irizarry RA, Astrand M, Speed TP: **A comparison of normalization methods for high density oligonucleotide array data based on variance and bias**. *Bioinformatics* 2003, **19**(2):185-193.
6. Luo J, Schumacher M, Scherer A, Sanoudou D, Megherbi D, Davison T, Shi T, Tong W, Shi L, Hong H *et al*: **A comparison of batch effect removal methods for enhancement of prediction performance using MAQC-II microarray gene expression data**. *Pharmacogenomics J* 2010, **10**(4):278-291.
7. Johnson WE, Li C, Rabinovic A: **Adjusting batch effects in microarray expression data using empirical Bayes methods**. *Biostatistics* 2007, **8**(1):118-127.
8. Hodges A, Strand AD, Aragaki AK, Kuhn A, Sengstag T, Hughes G, Elliston LA, Hartog C, Goldstein DR, Thu D *et al*: **Regional and cellular gene expression changes in human Huntington's disease brain**. *Hum Mol Genet* 2006, **15**(6):965-977.
9. Runne H, Regulier E, Kuhn A, Zala D, Gokce O, Perrin V, Sick B, Aebischer P, Deglon N, Luthi-Carter R: **Dysregulation of gene expression in primary neuron models of Huntington's disease shows that polyglutamine-related effects on the striatal transcriptome may not be dependent on brain circuitry**. *J Neurosci* 2008, **28**(39):9723-9731.

10. Oldham MC, Konopka G, Iwamoto K, Langfelder P, Kato T, Horvath S, Geschwind DH: **Functional organization of the transcriptome in human brain**. *Nat Neurosci* 2008, **11**(11):1271-1282.

4. Supplementary Figures

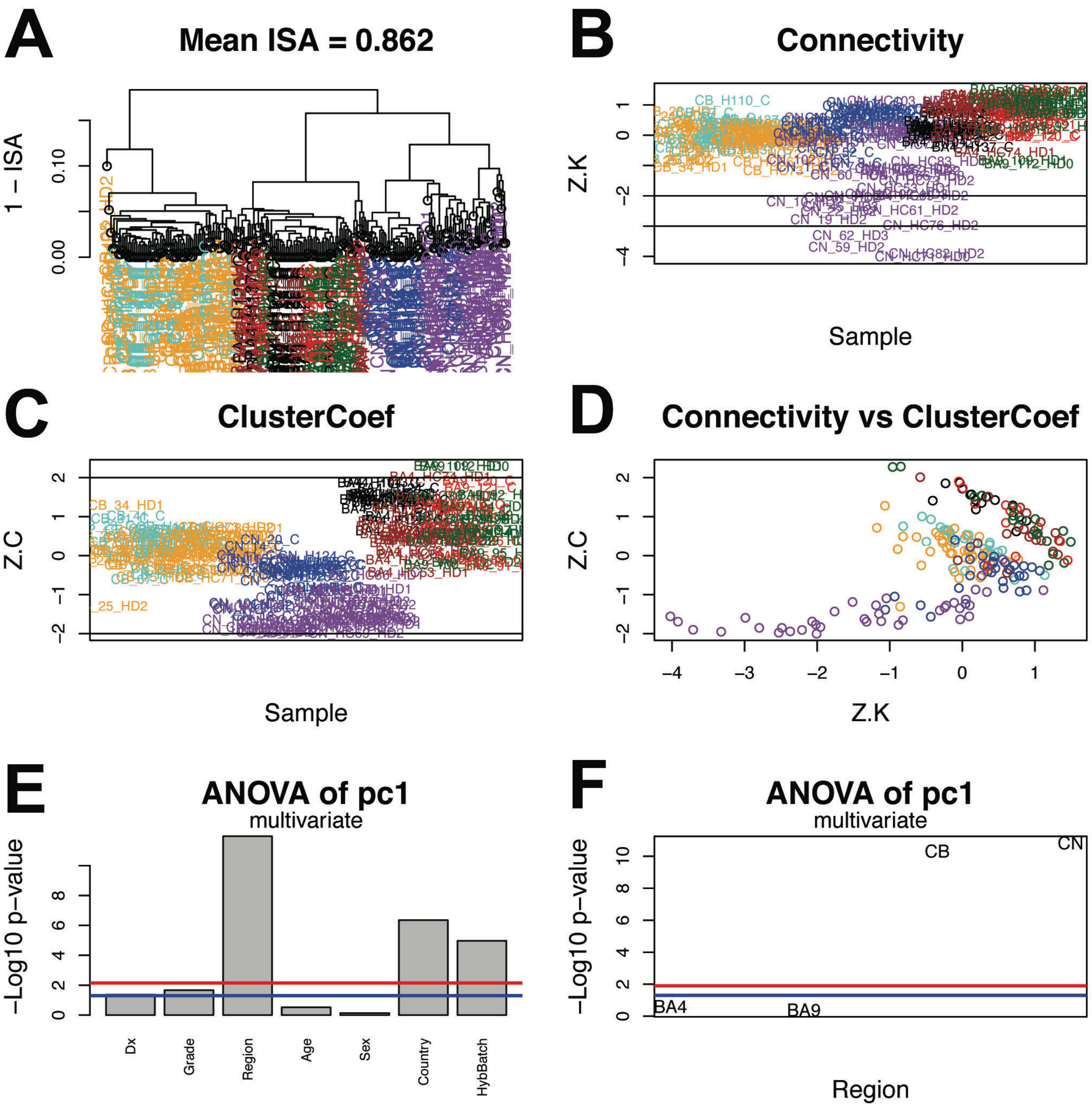

Supplementary Figure 1 | Sample networks provide a novel perspective on Huntington's disease. Analysis of 201 human brain microarray samples from [8] using SampleNetwork. (**A**) Dendrogram produced by average linkage hierarchical clustering using 1 – ISA (intersample adjacency) for all probe sets (n = 18,631) as a dissimilarity measure. Samples were colored according to brain region and HD diagnosis status: turquoise = CB CTRL (n = 27); orange = CB HD (n = 39); red = BA9 CTRL (n = 12); darkgreen = BA9 HD (n = 18); black = BA4 CTRL (n = 16); brown = BA4 HD (n = 19); blue = CN CTRL (n = 32); purple = CN HD (n = 38). CB = cerebellum; BA9 = Brodmann's area 9 (prefrontal cortex); BA4 = Brodmann's area 4 (primary motor cortex); CN = caudate nucleus; CTRL = control; HD = Huntington's disease. Standardized sample connectivities (*Z.K*; **B**) and standardized sample clustering coefficients (*Z.C*; **C**) for the same samples using all probe sets. Samples were colored as in (A). (**D**) The relationship between *Z.K* and *Z.C* is shown for all samples (colored as in [A]). (**E**) Significance testing of sample covariates using multivariate linear regression with pc1 as the outcome (i.e. the first principal component for all probe sets obtained via singular value decomposition). Blue line: $P = .05$; red line: Bonferroni correction for multiple comparisons. (**F**) Using the same model as in (E) but sub-setting by brain region isolated the significance levels of individual brain regions with respect to pc1.

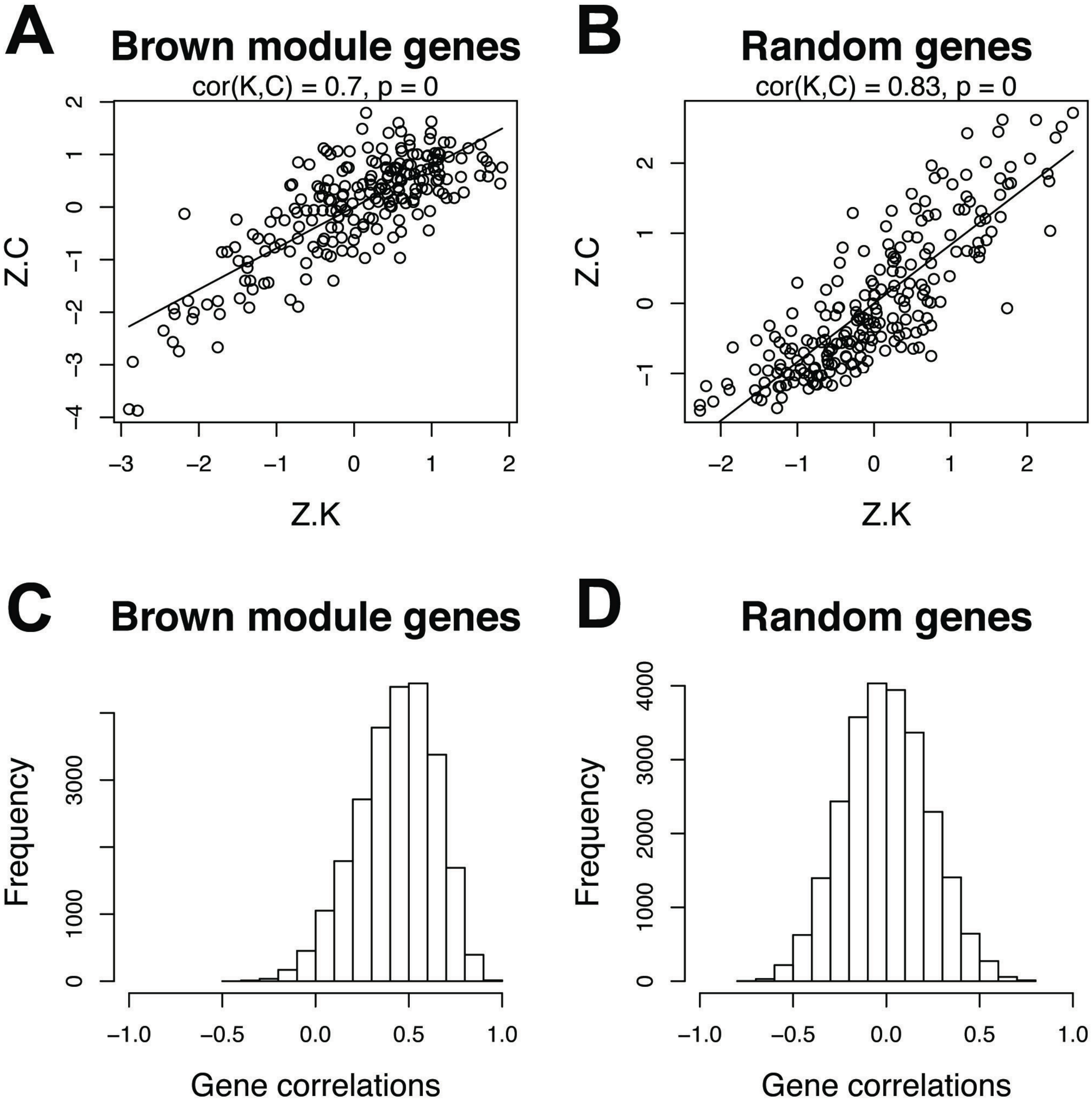

Supplementary Figure 2 | Connectivity is positively correlated with the clustering coefficient in modular and random gene expression networks. A module of co-expressed genes (M15C [brown], n = 221; [10]) (**A**) and 221 randomly selected genes (**B**) were used to construct signed weighted gene networks ($\beta = 2$) from human caudate nucleus control subjects (n=31; [8]). The brown module was chosen to approximate the number of nodes found in the HD study sample network (n = 201), and genes were restricted to those that were positively correlated with the brown module eigengene [1]. The standardized connectivity (*Z.K*) and standardized clustering coefficient (*Z.C*) exhibited strong positive correlations in each gene network (linear least squares regression lines in black). Note that the mean *Z.K* and mean *Z.C* were substantially higher in the module network (A), as expected. (**C** and **D**) Histograms of pairwise gene correlations are shown for the module gene network (A) and the random gene network (B), respectively.

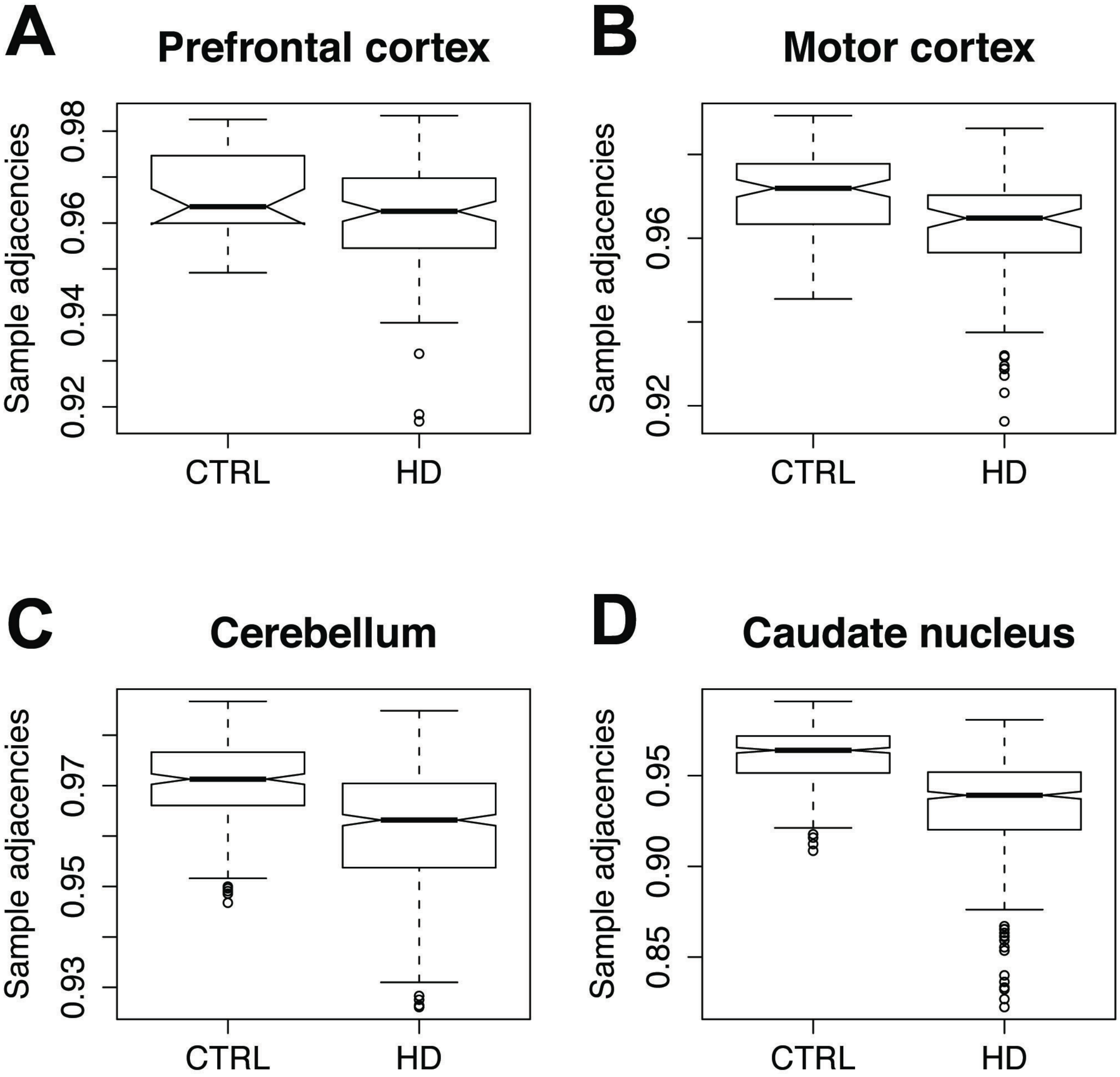

Supplementary Figure 3 | Sample adjacencies are degraded in caudate nucleus relative to other brain regions. Distributions of pairwise samples adjacencies for control (CTRL) and Huntington's disease (HD) subjects across all probe sets (n = 18,631) in prefrontal cortex (**A**; n = 9 CTRL and 16 HD), motor cortex (**B**; n = 16 CTRL and 14 HD), cerebellum (**C**; n = 23 CTRL and 34 HD), and caudate nucleus (**D**; n = 31 CTRL and 35 HD). Networks were constructed using all samples (CTRL and HD) from each brain region, and pairwise adjacencies among samples from each brain region x diagnosis cohort are shown.

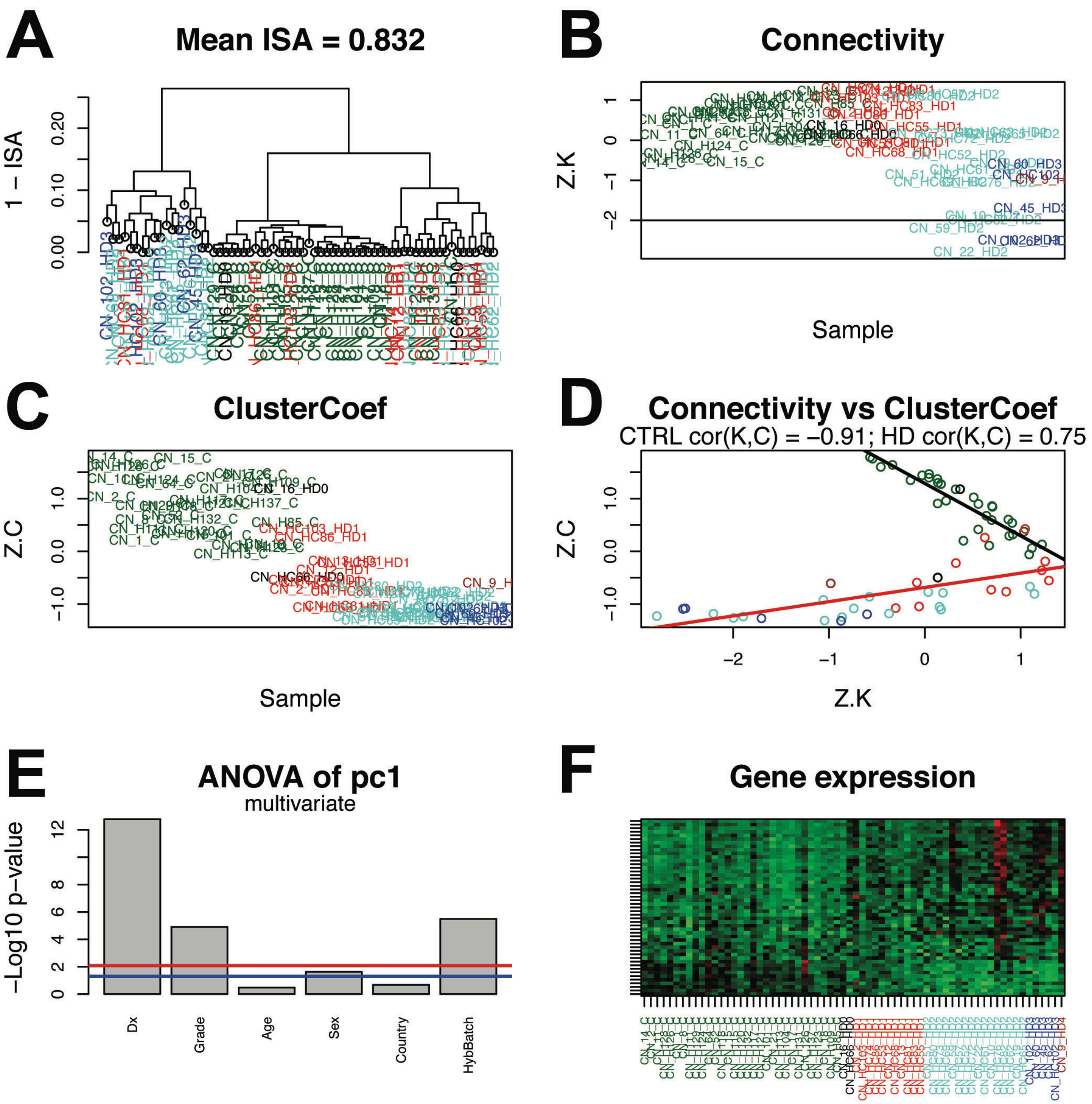

Supplementary Figure 4 | Caudate nucleus samples exhibit significant segregation by diagnosis in gene co-expression module M11C (black). Analysis of caudate nucleus (CN) sample network properties for genes comprising the CN black co-expression module M11C [10]. (**A**) Average linkage hierarchical clustering of samples using 1 – ISA (intersample adjacency). Colors denote control (CTRL) subjects (darkgreen; n = 31) and Huntington's disease (HD) subjects with varying grades of disease severity: HD grade 0 (black; n = 2), HD grade 1 (red; n = 11), HD grade 2 (turquoise; n = 16), HD grade 3 (blue; n = 5), and HD grade 4 (brown; n = 1). Standardized sample connectivities (*Z.K*; **B**) and standardized sample clustering coefficients (*Z.C*; **C**). (**D**) HD and CTRL samples segregated into two distinct groups when depicted in terms of *Z.K* and *Z.C* (linear least squares regression lines in black [CTRL] and red [HD]). (**E**) Multivariate linear regression revealed a significant effect of diagnosis (Dx) on the black module eigengene. Blue line: $P = .05$; red line: Bonferroni correction for multiple comparisons. (**F**) Heat map of expression levels for genes comprising the black co-expression module M11C. Rows correspond to probe sets (genes) and columns correspond to samples. Green = low expression; red = high expression. Note that the prevailing pattern of gene expression is opposite that seen in the salmon module M8C (Figure 6F from the main article). Samples in (B–D, F) are colored as in (A).

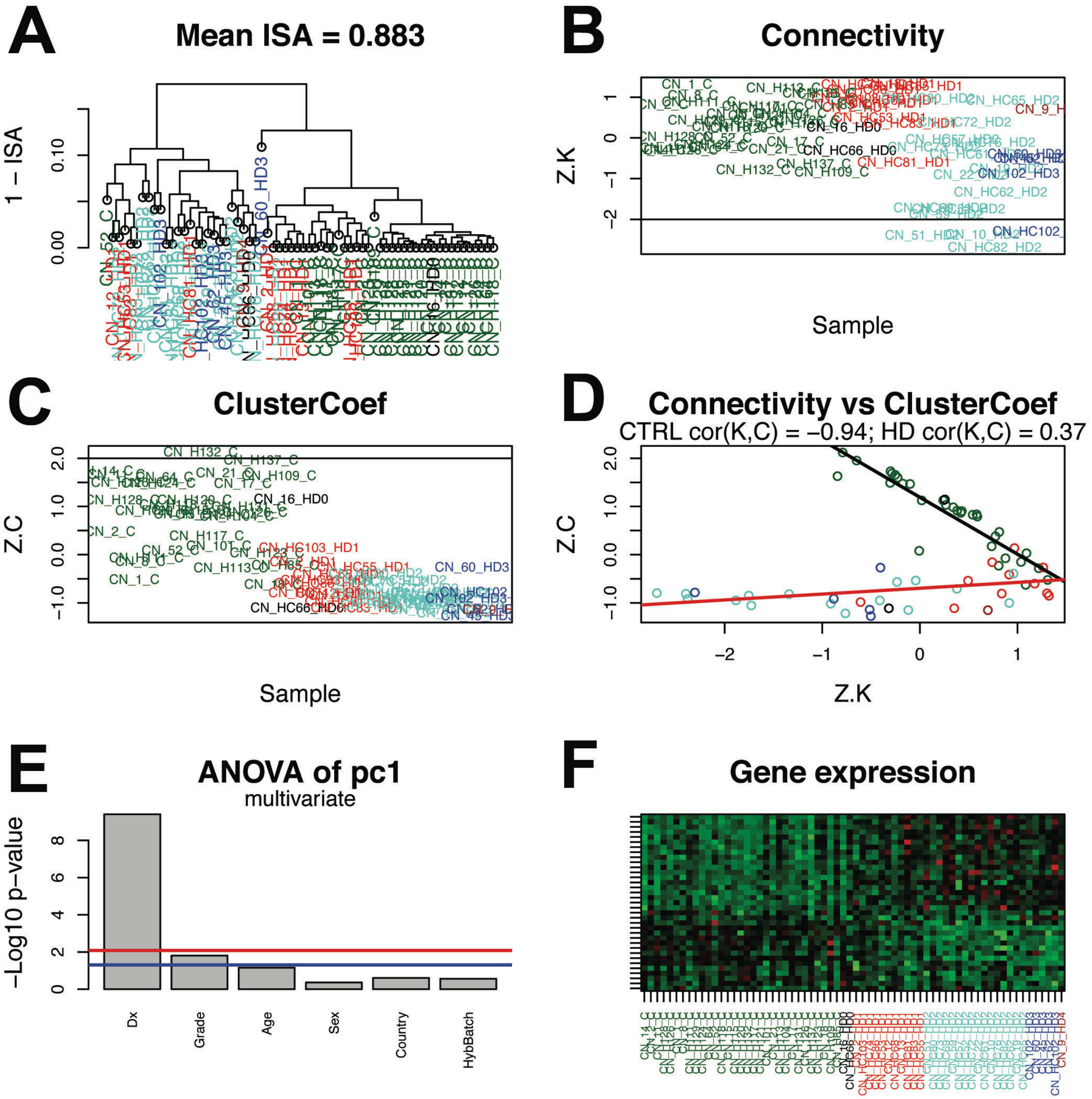

Supplementary Figure 5 | Caudate nucleus samples exhibit significant segregation by diagnosis in gene co-expression module M36 (royalblue). Analysis of caudate nucleus (CN) sample network properties for genes comprising the CN royalblue co-expression module M36 [10]. (**A**) Average linkage hierarchical clustering of samples using 1 – ISA (intersample adjacency). Colors denote control (CTRL) subjects (darkgreen; n = 31) and Huntington's disease (HD) subjects with varying grades of disease severity: HD grade 0 (black; n = 2), HD grade 1 (red; n = 11), HD grade 2 (turquoise; n = 16), HD grade 3 (blue; n = 5), and HD grade 4 (brown; n = 1). Standardized sample connectivities (*Z.K*; **B**) and standardized sample clustering coefficients (*Z.C*; **C**). (**D**) HD and CTRL samples segregated into two distinct groups when depicted in terms of *Z.K* and *Z.C* (linear least squares regression lines in black [CTRL] and red [HD]). (**E**) Multivariate linear regression revealed a significant effect of diagnosis (Dx) on the royalblue module eigengene. Blue line: $P = .05$; red line: Bonferroni correction for multiple comparisons. (**F**) Heat map of expression levels for genes comprising the royalblue co-expression module M36. Rows correspond to probe sets (genes) and columns correspond to samples. Green = low expression; red = high expression. Note that the prevailing pattern of gene expression is opposite that seen in the salmon module M8C (Figure 6F from the main article). Samples in (B–D, F) are colored as in (A).

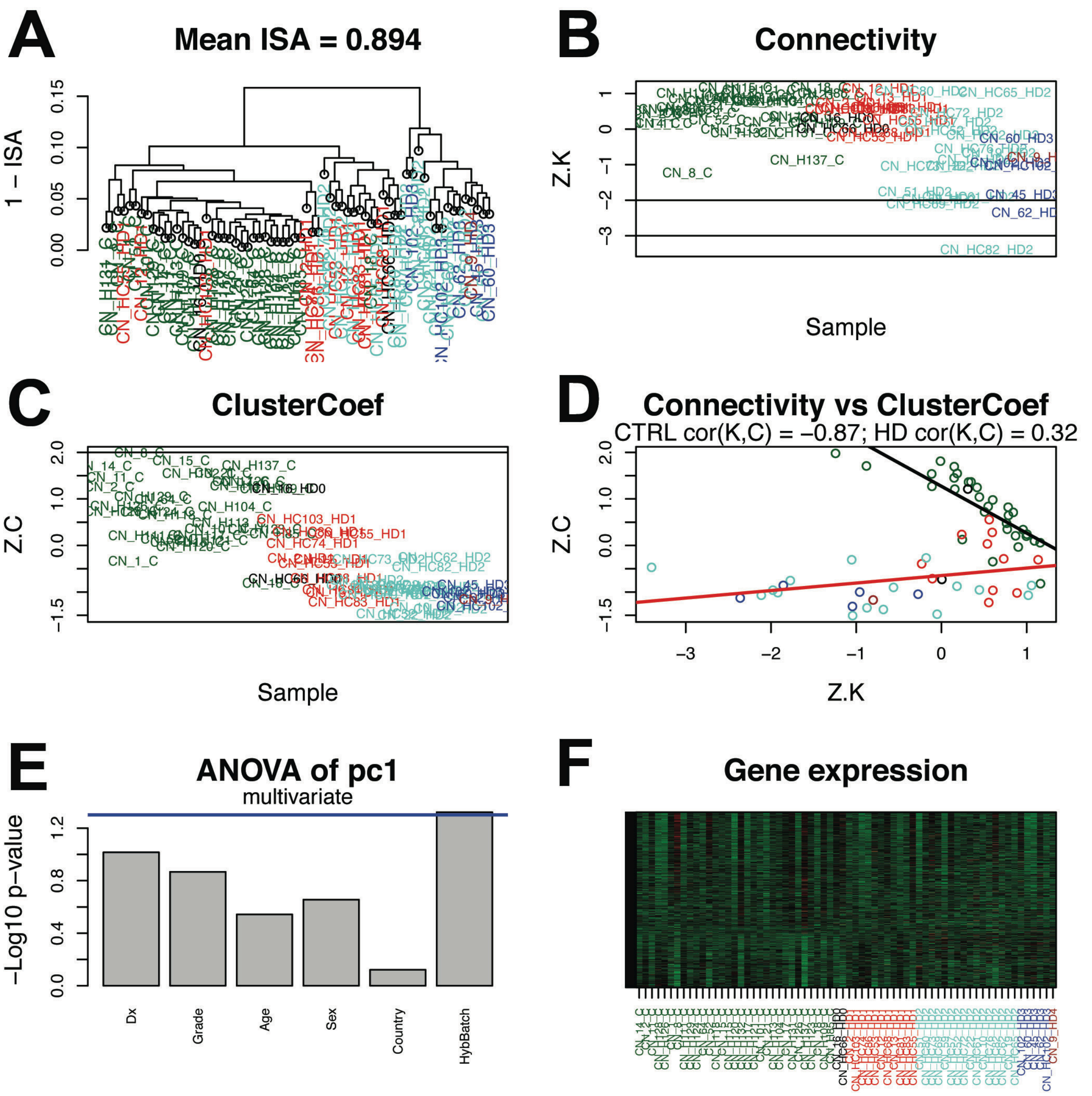

Supplementary Figure 6 | Caudate nucleus samples exhibit significant segregation by diagnosis in gene co-expression module M19C (red). Analysis of caudate nucleus (CN) sample network properties for genes comprising the CN red co-expression module M19C [10]. (**A**) Average linkage hierarchical clustering of samples using 1 – ISA (intersample adjacency). Colors denote control (CTRL) subjects (darkgreen; n = 31) and Huntington's disease (HD) subjects with varying grades of disease severity: HD grade 0 (black; n = 2), HD grade 1 (red; n = 11), HD grade 2 (turquoise; n = 16), HD grade 3 (blue; n = 5), and HD grade 4 (brown; n = 1). Standardized sample connectivities (*Z.K*; **B**) and standardized sample clustering coefficients (*Z.C*; **C**). (**D**) HD and CTRL samples segregated into two distinct groups when depicted in terms of *Z.K* and *Z.C* (linear least squares regression lines in black [CTRL] and red [HD]). (**E**) Multivariate linear regression revealed no significant effect of diagnosis (Dx) on the red module eigengene. Blue line: $P = .05$; red line: Bonferroni correction for multiple comparisons. (**F**) Heat map of expression levels for genes comprising the red co-expression module M19C. Rows correspond to probe sets (genes) and columns correspond to samples. Green = low expression; red = high expression. Samples in (B–D, F) are colored as in (A).

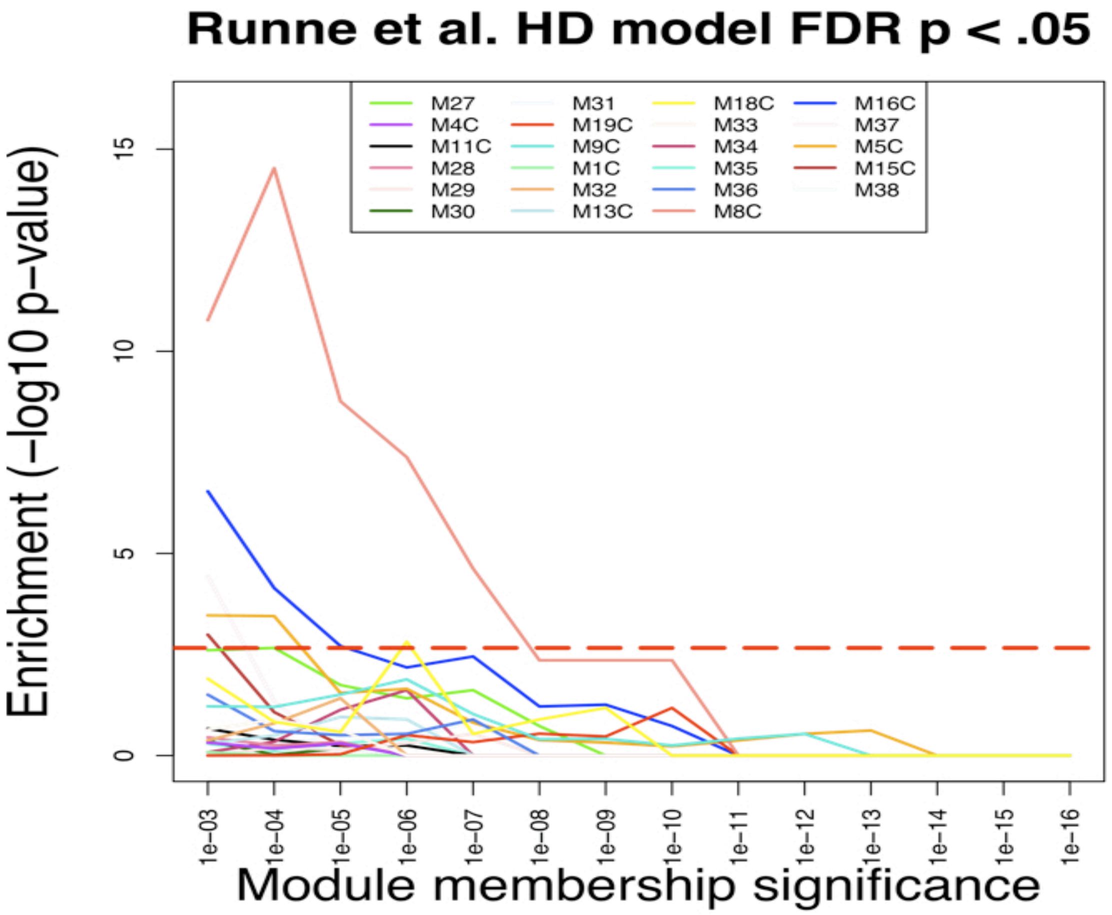

Supplementary Figure 7 | Module enrichment analysis of differentially expressed genes in an *in vitro* model of Huntington's disease. Genes that were differentially expressed (DE) in an *in vitro* model of Huntington's disease (HD), comprised of rat primary striatal neurons expressing an N-terminal fragment of the mutant huntingtin protein [9], were cross-referenced with human caudate nucleus (CN) gene co-expression modules from normal subjects [10]. The significance of enrichment (y-axis) is reported for various levels of stringency for module definitions (x-axis). Modules were iteratively re-defined at various levels of stringency by including all genes with positive module membership values (i.e. positive correlations with the module eigengene) and module membership P-values < 1e–03, < 1e–04, < 1e–05, etc. (as reported in Table S5 from [10]). DE genes (n = 1,036) were restricted to those with a false-discovery rate (Q-value) < .05 and with expression changes that were concordant with the direction of change in gene expression between control and HD subjects in caudate nucleus (Table S2 from [9]). Each line corresponds to a module, as denoted by its color and the legend. Note that M8C = the salmon CN module.

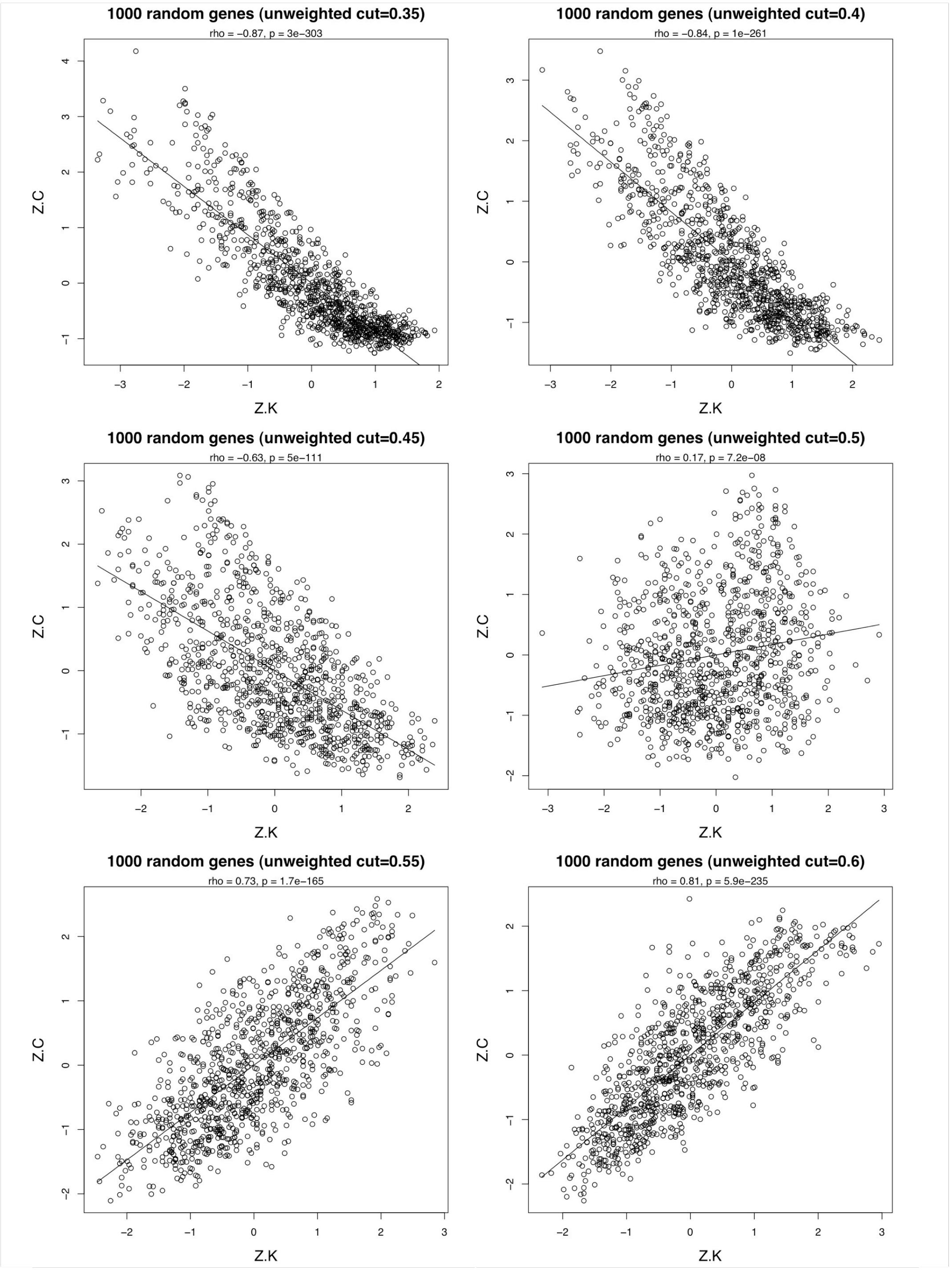

Supplementary Figure 8 | *cor*(K,C) exhibits similar behavior in unweighted networks. Here we explore the properties of the standardized $C(k)$ curve in signed unweighted networks using hard thresholds. As the threshold for dichotomizing the adjacency matrix to produce an unweighted network is progressively increased (from top left to bottom right), we observe a transition in the standardized $C(k)$ curve that is similar to the transition we observe in weighted sample networks. For the examples shown below, 1000 randomly selected genes (probe sets) were used to construct signed unweighted gene networks from human

caudate nucleus control subjects (n=31; [8]). Thus, at permissive (low) thresholds (e.g. top left panel), which produce networks in which most nodes are connected, the standardized *C*(*k*) curve is negative; as the threshold is raised, producing networks in which most nodes are not connected, the relationship begins to invert, becoming positive at more stringent (high) thresholds (e.g. bottom right).
