# Supplementary material for: Network methods for describing sample relationships in genomic datasets: application to Huntington’s disease
Source: BMC Syst Biol. 2012 Jun 12;6:63. doi: 10.1186/1752-0509-6-63 (PMC3441531; doi:10.1186/1752-0509-6-63)
Supplement: Additional file 3 — SampleNetwork R tutorial. DOC file containing annotated R code and detailed instructions for executing the SampleNetwork and ModuleSampleNetwork R functions. The datasets that are referenced in the tutorial and analyzed in this study can be downloaded from: http://www.genetics.ucla.edu/labs/horvath/CoexpressionNetwork/SampleNetwork. [file 1752-0509-6-63-S3.doc]

**ADDITIONAL FILE 3: SAMPLE NETWORK R TUTORIAL**

**Sample Networks for Enhancing Cluster Analysis of Genomic Data: Application to Huntington's Disease**

Michael C. Oldham, Peter Langfelder, and Steve Horvath

Correspondence: [oldhamm@stemcell.ucsf.edu](mailto:oldhamm@stemcell.ucsf.edu) and [shorvath@mednet.ucla.edu](mailto:shorvath@mednet.ucla.edu)

**Introduction**

This R tutorial describes how to use the SampleNetwork R function to construct sample networks from large genomic (or other) datasets. As described in the corresponding journal article, sample networks can enhance the results of cluster analysis for outlier detection, identification of sample characteristics (traits) with global effects, and group comparisons using pre-selected lists of features (e.g. transcripts). A major advantage of constructing sample networks is that individual samples can subsequently be described using established node-based network concepts such as the connectivity and the clustering coefficient. These concepts are invariant with respect to the choice or use of clustering algorithms, and depend only on the adjacency measure used to construct the network. In our implementation, we define adjacencies among samples as signed weighted correlations with values that approximate the underlying correlations when these correlations are large, as is usually the case in sample networks. A signed weighted correlation network is attractive since it preserves sign information, is robust with respect to the soft threshold (power) parameter (β), and preserves the continuous nature of correlations (i.e. the result is a fully connected network in which all nodes are neighbors with one another) [1].

Within this framework, the distributions of standardized node-based measures such as the connectivity can reveal outlying samples in an objective and quantifiable fashion that depends only on the choice of the network adjacency measure. By relating the characteristics of individual samples (nodes) to the characteristic of the group (network), our approach provides an unbiased and encompassing framework for identifying samples that "behave" differently, even if the underlying causes of this behavior are unknown. Intuitively, if the connectivity for a given sample (when measured over all genes) is significantly lower than all other sample connectivities from the same biological system, it suggests that there is something different about that sample compared to the others. The investigator must ask him/herself whether the observed difference is likely to reflect biological or technical variation. In light of the multiple steps that comprise a typical genomic experiment, each of which may introduce technical variation, a conservative approach is to exclude aberrant samples if there are no obvious biological factors that might explain their discordant behavior.

Compared with other methods for identifying outlying samples in genomic data, our approach offers several additional advantages. First, it is platform-agnostic and does not require access to raw data (although in practice it is preferable to process raw data in a consistent fashion), since it is defined with respect to a correlation matrix. Second, it is easily applied to very large datasets, in contrast to clustering procedures that rely upon visual inspection of dendrograms to identify outlying samples. Third, it produces a battery of measures for summarizing the consistency and integrity of genomic datasets (e.g. mean ISA [density], decentralization, homogeneity, etc.), which can be compared across disparate studies, technology platforms, and biological systems. Such measures are especially useful for meta-analyses, where objective assessment of data quality is highly desirable before seeking to pool or compare results across datasets. Finally, as implemented in the SampleNetwork R function and described below, our approach is both flexible and efficient, enabling users to move quickly through large datasets in an iterative fashion, specifying groups of samples for processing, identifying and removing outliers, testing the significance of sample covariates, and performing data normalization. To enhance user-friendliness, we have also incorporated the R function ComBat [2], which we have found to be a very effective tool for removing batch effects (see Text S1 from the journal article). At each stage of processing, relevant output files are produced and exported automatically.

We illustrate our approach using the same microarray dataset [3] analyzed in the corresponding journal article. This dataset was generated from brain samples of patients with Huntington's disease (HD; n = 44 individuals) and unaffected controls (CTRL; n = 36 individuals, matched for age and sex). The authors of this study [3] used Affymetrix U133A microarrays to survey gene expression in caudate nucleus (CN), cerebellum (CB), primary motor cortex (Brodmann's area 4; BA4), and prefrontal cortex (Brodmann's area 9; BA9) in the CTRL group and across five grades of HD severity, which were scored between 0 (least severe) and 4 (most severe) using Vonsattel's neuropathological criteria [4]. In addition to disease status and severity, sample information included age, sex, the country where the experiment was performed (samples were processed in the United States and New Zealand), and the microarray hybridization batch. In light of these myriad biological and technical sources of variation, this dataset presents a challenging analytical task.

In this tutorial we highlight the required input files, parameter choices, user interactions, and resulting output files of the SampleNetwork function. The beneficial effects of using the SampleNetwork approach are clearly delineated by significance testing of sample covariates with respect to sample metrics, analysis of differential expression, and analysis of network concepts with each successive round of data processing, as illustrated below.

Beyond facilitating relatively simple analytical tasks such as outlier identification, sample networks also provide a novel perspective on more complex analytical challenges such as group comparisons. Therefore, in this tutorial we also describe how to use the companion R function ModuleSampleNetwork to compare properties of sample networks among sample subgroups when formed over subsets of features. ModuleSampleNetwork does not enable outlier identification and removal or data normalization, but instead seeks to facilitate supervised comparisons of module sample network properties. We illustrate the ModuleSampleNetwork function using the same microarray dataset [3] described above.

**SampleNetwork**

**Getting the software and the data**

1. Download the R software at <http://www.r-project.org/> and install it on your computer. After installing R, you will need to install several additional R library packages. For example, to install the package WGCNA, open R, go to the "Packages and Data" menu and select "Package Installer...", then choose WGCNA. R will automatically install the package. Do the same for the impute and cluster packages. The affy package is from Bioconductor (<http://www.bioconductor.org/>). To install this package, open an R session and type:

source("http://bioconductor.org/biocLite.R")

biocLite()

This will install a core selection of Bioconductor packages, including affy. To install the preprocessCore package (another Bioconductor package), type:

biocLite("preprocessCore")

1. Download the .zip file "SampleNetwork files" at: http://www.genetics.ucla.edu/labs/horvath/CoexpressionNetwork/SampleNetwork. There are two .csv files in this .zip file:

- GSE3790_expression_data.csv: This file contains the expression data from "the HD study" [3], which have been minimally processed as described in Materials and Methods from the journal article.
- GSE3790_sample_information.csv: This file contains the sample information for the HD study (i.e. sample labels, sample traits, and sample grouping information).

1. Download the SampleNetwork R functions file at: http://www.genetics.ucla.edu/labs/horvath/CoexpressionNetwork/SampleNetwork. This file contains the SampleNetwork_1.0 R function and several other required functions. In this file you will also find a brief description of the arguments for the SampleNetwork function. DISCLAIMER: Absolutely no warranty on the code. Please contact MCO ([oldhamm@stemcell.ucsf.edu](mailto:oldhamm@stemcell.ucsf.edu)) with bug reports or suggestions.
2. Unzip SampleNetworkFiles.zip and place the files in the same directory as SampleNetwork_1.0.r, e.g.: "/Users/mcoldham/Documents/SampleNetworkTutorial"

**SampleNetwork: usage and defaults**

SampleNetwork requires two input files: (1) a file containing the feature activity (e.g. gene expression data; see GSE3790_expression_data.csv above for an example), and (2) a file containing the sample information (i.e. sample traits; see GSE3790_sample_information.csv above for an example). The usage and default settings of the SampleNetwork_1.0 R function are described below, followed by a brief description of each argument and its corresponding options.

**Usage**

SampleNetwork(

datExprT,

method1="correlation",

impute1=FALSE,

subset1=NULL,

skip1,

indices1,

sampleinfo1,

subgroup1,

samplelabels1,

grouplabels1,

fitmodels1=FALSE,

whichmodel1="univariate",

whichfit1="pc1",

btrait1=NULL,

trait1=NULL,

asfactors1=NULL,

projectname1,

cexlabels1=0.7,

normalize1=TRUE,

replacenegs1=FALSE,

exportfigures1=TRUE,

verbose=TRUE

)

**Arguments**

datExprT Expression data (or other types of data) in the form of a data frame where rows are features (e.g. probe sets) and columns are samples.

method1 Controls which type of similarity measure will be used to define a measure of distance among samples. Choices are "correlation" (Pearson; default) or "euclidean" for Euclidean distance. Any other value will trigger an error.

impute1 If TRUE, expression data will be checked for the presence of NA entries; if present, numerical data will be imputed for these entries, using the function impute.knn. The function impute.knn uses a fixed random seed giving repeatable results. If FALSE (default), no imputation will be performed.

subset1 A binary indicator vector (0,1) that can be used to specify a subset of features for running SampleNetwork. The length of the vector must equal the number of features. By default (NULL), all features will be used.

skip1 An integer describing the number of feature information columns in datExprT (e.g. probe set ID, gene symbol, etc.). Note: there must be at least one column of feature information (unique identifiers) in datExprT.

indices1 A list of vectors that indicates if there are groups of samples to be processed separately by SampleNetwork. Each vector is used to subset the samples (columns) in datExprT.

sampleinfo1 Sample traits in the form of a data frame where rows are samples and columns are traits.

subgroup1 An integer that points to the column number in sampleinfo1 that specifies sample subgroups to be colored separately in plots (default = NULL).

samplelabels1 An integer that points to the column number in sampleinfo1 containing the sample labels that will appear in plots. Note: these sample labels must be identical to the sample column headers in datExprT or an error will be triggered.

grouplabels1 An integer that points to the column number in sampleinfo1 that contains labels for the sample groups specified by indices1. Note: the number of unique group labels in this column must equal the number of vectors (indices) specified by indices1.

fitmodels1 Logical: should linear regression models be constructed and the significance of sample traits assessed with respect to a summary measure of datExprT? If FALSE (default), the number of panels in each plot will be reduced. If TRUE, analysis of variance (ANOVA) will be used to assess the significance of specified sample traits.

whichmodel1 If fitmodels1 = TRUE, should a univariate (default) or multivariate linear regression model be constructed?

whichfit1 If fitmodels1 = TRUE, which summary measure of datExprT should be used as the outcome for model-fitting? Choices are pc1 (default; i.e. the first principal component obtained by singular value decomposition), mean (sample means), and K (sample connectivities).

btrait1 If fitmodels1 = TRUE, a vector of integers that specifies the column numbers (traits) in sampleinfo1 that should be included in model-fitting; otherwise, NULL.

trait1 If fitmodels1 = TRUE, a vector of integers that specifies the column numbers (traits) in sampleinfo1 that should be tested for the significance of individual factor levels in model-fitting; otherwise, NULL. Note: trait1 must be a subset of btrait1 and can specify only categorical variables.

asfactors1 If fitmodels1 = TRUE, a vector of integers that specifies the column numbers (traits) in sampleinfo1 that should be treated as factors (i.e. categorical variables) in model-fitting; otherwise, NULL.

projectname1 Character string that provides a convenient label for the project; this string will appear as part of the filename for some output files.

cexlabels1 Numeric scaling factor for sample labels in plots (default = 0.7).

normalize1 Logical: should normalization be performed at the feature level? If TRUE (default), quantile normalization [5] will be performed. If FALSE, no quantile normalization will be performed; however, the user will still have the option to perform batch normalization via ComBat [2].

replacenegs1 Logical: should negative expression (or other) values introduced by ComBat [2] or already present in the data be replaced by the median for the corresponding feature? Default = FALSE.

exportfigures1 Logical: should figures generated by the function be exported as .pdfs? If TRUE (default), all of the figures generated after each round of outlier removal, normalization, and correction for batch effects, as specified by the user through interactions with the function, will be exported to the SampleNetwork subdirectory.

verbose Logical: should additional network metrics (Z.C) and cor(K,C) be reported and appear in plots? If FALSE, only hierarchical clustering of samples and Z.K will appear. Default = TRUE.

**Details**

SampleNetwork has been designed to facilitate flexible, interactive, and iterative exploration of sample networks. To derive maximum utility from the function, we prefer to apply SampleNetwork to gene expression data that have been minimally processed in a consistent fashion, as described in Materials and Methods from the journal article. In principle, however, SampleNetwork can be applied to any feature data that can be represented in the form of a matrix. Missing data can be inferred by setting the argument impute1 = TRUE. The user can process all samples simultaneously or in groups, as indicated by the argument grouplabels1, which specifies a column number in sampleinfo1. For example, if the user wishes to process all samples simultaneously, the column specified by grouplabels1 would simply contain the same value for all samples (e.g. column 18 of GSE3790_sample_information.csv). Continuing with the example of the HD study, if the user wishes to process all samples from each brain region separately, this would be done by setting grouplabels1 = 7 (i.e. column 7 of GSE3790_sample_information.csv). Note that in this case, data from each brain region will be analyzed separately, which may be desirable for some downstream applications (e.g. coexpression analysis), but not others (e.g. differential expression analysis).

The argument method1 currently implements two types of similarity measures for constructing sample networks: correlation and Euclidean distance. In practice, we find it advantageous to use a correlation-based distance measure (the default) for constructing sample networks, for reasons described in the journal article and above. Although we have not thoroughly investigated the properties of sample networks constructed using Euclidean distance, there may be situations in which it is preferable to use Euclidean distance as a similarity measure.

Gene expression or other feature data may sometimes contain negative values, depending upon processing algorithms. We note that ComBat [2] may occasionally introduce negative expression values after correcting for batch effects. Negative expression values may be undesirable for some downstream applications. Therefore, we have included an option to replace negative expression values with the median of the corresponding feature by setting the argument replacenegs1 = TRUE. Alternatively, the user may choose to replace negative values manually using a different approach by manipulating the exported data files. In this case, the user should set replacenegs1 = FALSE.

Detailed sample information is not required to perform sample outlier detection and removal or quantile normalization. However, model-fitting and identification/correction of batch effects require that sample traits of interest are present in the sample information file. For an investigator designing a new experiment, we recommend carefully balancing and tracking all potential sources of technical variation. For example, in a microarray experiment, these might include sample batch information for RNA extractions, sample RNA integrity (RIN) scores, hybridization batch information, and array batch information. It is critical that the biological effects under examination are not confounded with any significant sources of technical variation. For example, it is a bad idea to hybridize control samples in one batch and treatment samples in a separate batch if the goal is to perform a differential expression analysis between the groups.

When fitting linear models, SampleNetwork will exclude wholly redundant factor levels and factor levels with only one constituent (see below). In these cases, warning messages will be produced. It is important to specify categorical variables as such via the argument asfactors1 to ensure the accuracy of linear models. We recommend using pc1 (the default) as the summary measure (outcome) for linear models. In practice, we have found that P-values obtained using mean (mean expression) are very similar to those obtained via pc1, but slightly less significant. Linear models constructed using K (connectivity) as the outcome often produce different results.

If a technical sample trait (e.g. hybridization batch) shows a significant association with the expression summary measure after linear modeling, the user may wish to perform batch normalization to remove this effect from the data. Batch normalization is performed by calling the ComBat function [2] using default parameters from within the SampleNetwork function. Note that ComBat can be executed independently of outlier removal or standard (quantile [5]) normalization. For example, to run only ComBat, the user would set the argument normalize1=FALSE (no quantile normalization), and type None when prompted to remove outliers. The user would then still be able to execute ComBat from within SampleNetwork. Note that a sample information file required by ComBat is generated automatically by SampleNetwork and exported to the /SampleNetwork subdirectory. Also note that the user has the option of supplying additional sample covariates (traits) to ComBat in addition to sample batch information. In general, the authors of ComBat recommend providing sample covariates whenever they are believed to have a significant effect on measured activity [5]. Covariates must not possess perfect concordance with batch structure (e.g. all control samples processed in one hybridization batch and all treatment samples in another), or an error will be triggered.

**Starting the R session**

Open the R software by double-clicking the desktop icon. To interact with the R software, copy and paste commands into the R console. There are a number of good introductory R tutorials that can be found simply by Googling "R tutorial".

Note: Text written after the "#" character is interpreted as a comment and is automatically ignored by R.

# Set the working directory of the R session to the directory containing the downloaded files, e.g.:

setwd("/Users/mcoldham/Documents/SampleNetworkTutorial")

# Load the SampleNetwork_1.0 R functions file:

source("SampleNetwork_1.0.r")

# Note: This file can also be opened with any text editor, and its contents copied and pasted directly into the R session. Note that the required libraries will be loaded automatically by virtue of reading in this file (assuming the packages are installed).

**Reading in the expression data and sample information file**

datE=read.csv("GSE3790_expression_data.csv")

datS=read.csv("GSE3790_sample_information.csv")

# Check the dimensions of datE:

dim(datE)

# [1] 18631 203

# So datE is a data frame containing 18,631 rows (corresponding to probe sets) and 203 columns (corresponding to 201 samples + 2 feature information columns: "Probe_set" and "Gene").

# Note: It is helpful to order samples according to biologically meaningful groupings, if possible. For example, we can see the order of the samples by typing:

colnames(datE)

# After the two feature information columns, samples have been grouped by brain region, where CB = cerebellum, CN = caudate nucleus, BA4 = motor cortex, and BA9 = prefrontal cortex.

# Check the dimensions of datS:

dim(datS)

# [1] 201 18

# So datS is a data frame containing 201 rows (corresponding to samples) and 18 columns (corresponding to sample labels, traits, and sample group information). We can see the column headers for datS by typing:

colnames(datS)

# [1] "Array" "Sample" "Label" "Platform" "Dx" "Grade" "Region" "Genotype" "GenNum" "GenDenom" "Age" "Sex" "Individual" "HybDate"

"HybBatch" "Country" "Dx_Region" "All"

# Note that one of the columns in datS must provide the sample labels, which in turn must match the sample column headers in datE identically. In this case, the third column ( "Label") contains the sample labels. To check that the sample labels in datS match the sample column headers in datE, type:

all(datS$Label==colnames(datE)[3:203])

# [1] TRUE

# where the subset [3:203] excludes the Probe_set and Gene columns.

# Note: It is a good idea to provide brief but informative sample labels. For example, in this case, our sample labels take the form CB_23_C, CB_24_HD2, etc., where the first field (e.g. CB) denotes the brain region, the next field denotes the individual (23 or 24), and the next field denotes the diagnosis (control or Huntington's disease). Note as well the number after HD (e.g. HD2), which denotes the grade of HD severity (e.g. grade 2).

**Running the SampleNetwork function: user interactions**

We will first illustrate the SampleNetwork function by analyzing all samples from the HD study, using the same settings that produced Figure S1 from the journal article. SampleNetwork may be executed by copying and pasting the following code into the R session:

SampleNetwork(

datExprT=datE,

method1="correlation",

impute1=FALSE,

subset1=NULL,

skip1=2,

indices1=list(c(3:203)),

sampleinfo1=datS,

subgroup1=17,

samplelabels1=3,

grouplabels1=18,

fitmodels1=TRUE,

whichmodel1="multivariate",

whichfit1="pc1",

btrait1=c(5,6,7,11,12,16,15),

trait1=c(7),

asfactors1=c(5,6,7,12,15,16),

projectname1="GSE3790",

cexlabels1=0.7,

normalize1=TRUE,

replacenegs1=FALSE,

exportfigures1=TRUE,

verbose=TRUE

)

## Let us briefly describe the parameters specified above in English:

## We will construct sample networks using expression data contained in datE (datExprT=datE) and correlation as a distance measure among samples (method1="correlation"). There are no missing data (impute1=FALSE), and we will use all probe sets (subset1=NULL). The first two columns of datE contain feature information (skip1=2), and we will process all samples simultaneously (indices1=c(3:203)). datS specifies the sample information file (sampleinfo1=datS). We will color samples in plots according to diagnosis (CTRL vs HD) and brain region (CB, CN, BA4, BA9), i.e. 8 colors, as specified in column 17 of datS (subgroup1=17). The sample labels in datS are in column 3 (samplelabels1=3), and the group labels (in this case, "All", since we are processing all samples simultaneously), are in column 18 (grouplabels1=18). We will perform multivariate linear regression analysis (fitmodels1=TRUE) and (whichmodel1="multivariate") using the first principal component of datE as the outcome measure (whichfit1="pc1"). We will include the sample traits that are present in columns 5, 6, 7, 11, 12, 16, and 15 of datS, in that order, for building the model (btrait1=c(5,6,7,11,12,16,15)). We will also test the significance of the individual levels of the categorical variable in column 7 of datS (trait1=c(7)). Among the sample traits we are including in the model, all but one (column 11) should be modeled as categorical variables (factors; asfactors1=c(5,6,7,12,16,15)). Note: it is very important that categorical variables be indicated as such via asfactors1 for accuracy in the modeling process. The name we will give the project is "GSE3790" (projectname1="GSE3790"), and the default setting for the size of sample labels in the plots will be retained (cexlabels1="GSE3790"). Quantile normalization will be performed (normalize1=TRUE), and negative expression values, should they be present, will not be replaced (replacenegs1=FALSE). PDFs of figures will be exported into the /SampleNetwork subdirectory (exportfigures1=TRUE), and additional plots of Z.C and cor(K,C) will be produced (verbose=TRUE).

## These parameters generate the following figure:


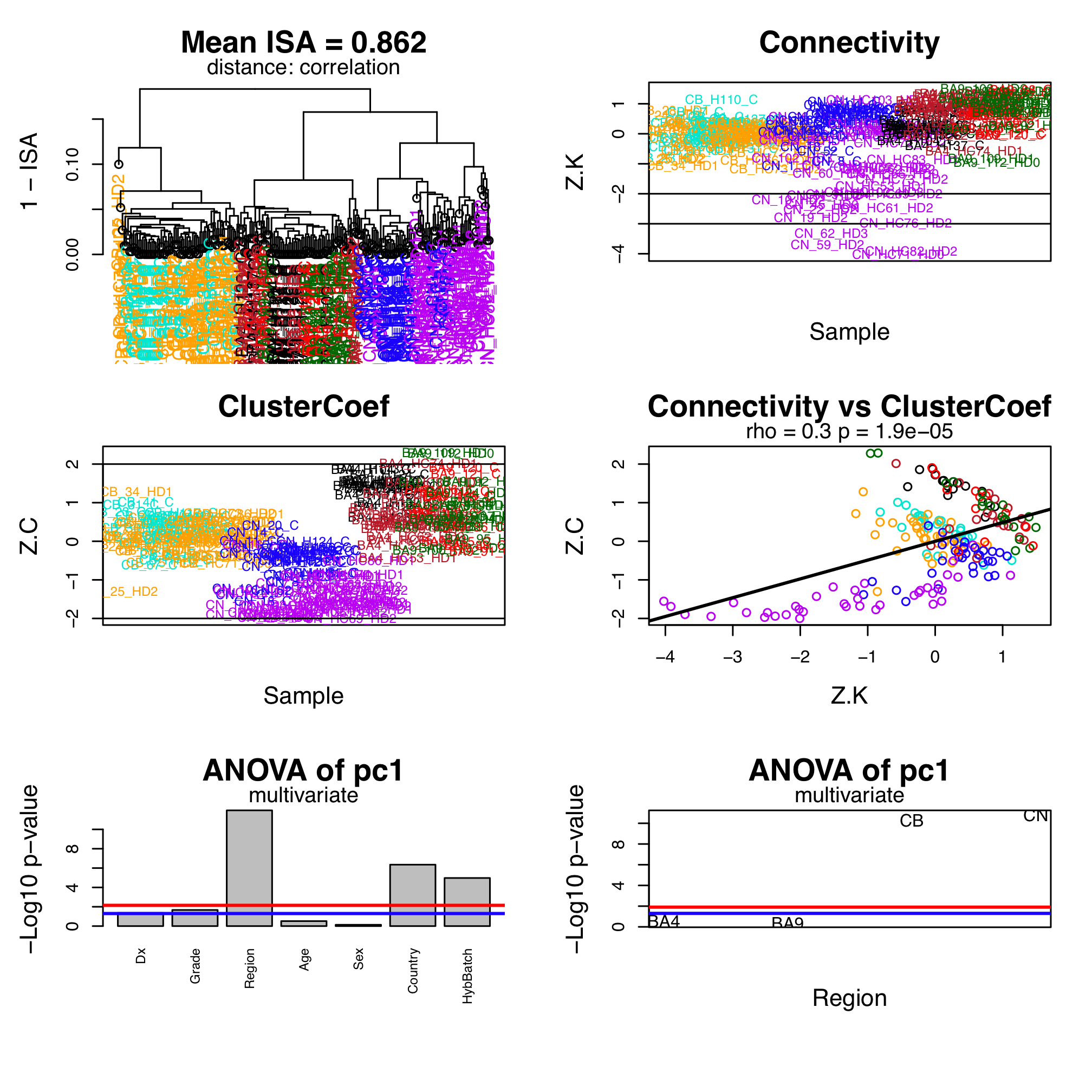


## In addition, a large table is returned within the R session, along with several warnings, some metrics, and a request for information:

All Label Dx Grade Region Age Sex Country HybBatch K Z.K Z.C Z.MAR

127 All CN_HC71_HD0 HD 0 CN 70 F NZ 16 0.860 -4.020000 -1.55000 -4.07000

134 All CN_HC82_HD2 HD 2 CN 74 M NZ 29 0.863 -3.920000 -1.69000 -3.91000

89 All CN_59_HD2 HD 2 CN 57 M US 29 0.868 -3.710000 -1.90000 -3.75000

91 All CN_62_HD3 HD 3 CN 56 F US 19 0.878 -3.320000 -1.95000 -3.38000

131 All CN_HC76_HD2 HD 2 CN 71 M NZ 29 0.886 -2.990000 -1.59000 -2.99000

80 All CN_19_HD2 HD 2 CN 79 F US 18 0.891 -2.800000 -1.85000 -2.81000

85 All CN_22_HD2 HD 2 CN 65 M US 19 0.898 -2.530000 -1.97000 -2.60000

121 All CN_HC61_HD2 HD 2 CN 65 M NZ 15 0.899 -2.490000 -1.85000 -2.53000

86 All CN_45_HD3 HD 3 CN 52 M US 29 0.902 -2.370000 -1.94000 -2.41000

68 All CN_10_HD2 HD 2 CN 70 M US 18 0.906 -2.210000 -1.90000 -2.24000

87 All CN_51_HD2 HD 2 CN 42 M US 18 0.910 -2.070000 -1.68000 -2.15000

******************** TRUNCATED FOR BREVITY *******************

162 All BA4_HC65_HD2 HD 2 BA4 82 F NZ 20 0.996 1.320000 0.02970 1.26000

165 All BA4_HC69_HD2 HD 2 BA4 50 F NZ 28 0.996 1.330000 0.39300 1.30000

192 All BA9_86_C C c BA9 51 M US 25 0.996 1.350000 0.36800 1.32000

195 All BA9_90_HD3 HD 3 BA9 52 M US 24 0.997 1.390000 0.63500 1.39000

186 All BA9_119_HD2 HD 2 BA9 70 M US 23 0.998 1.400000 -0.18000 1.33000

193 All BA9_88_C C c BA9 68 F US 26 0.999 1.450000 -0.11600 1.39000

173 All BA9_103_HD2 HD 2 BA9 65 M US 26 1.000 1.490000 0.35800 1.47000

[1] "Warning: the following coefficient was not defined because of a singularity: factor(sampleinfo2[indexgp2 - skip1, 6])c"

[2] "Warning: the following coefficient was not defined because of a singularity: factor(sampleinfo2[indexgp2 - skip1, 15])14"

[3] "Warning: the following coefficient was not defined because of a singularity: factor(sampleinfo2[indexgp2 - skip1, 15])28"

[1] "Group All mean correlation = 0.8558"

[1] "Group All mean connectivity = 172.5"

[1] "Group All mean scaled connectivity = 0.9622"

[1] "Group All mean clustering coefficient = 0.8637"

[1] "Group All mean maximum adjacency ratio = 0.8673"

[1] "Group All density = 0.8624"

[1] "Group All decentralization = 0.9657"

[1] "Group All homogeneity = 0.9737"

[1] "Group All PC 1-5 var explained = c(0.3407, 0.1278, 0.07704, 0.03041, 0.01927)"

[1] Please enter the row number(s) from the output of the sample(s) you would like to remove (comma separated) or type 'None'

[1] Alternatively, please enter a sample connectivity threshold for removing outliers. For example, type '<-2' to remove all samples with Z.K less than -2

Response:

## Let us digest this output. Each row in the table above corresponds to a sample, and the columns correspond to the sample traits specified by btrait1 as well as some newly calculated sample network metrics (K, Z.K, Z.C, and Z.MAR), which denote the connectivity, scaled connectivity, scaled clustering coefficient, and scaled maximum adjacency ratio, respectively (see Materials and Methods and Text S1 from the journal article). The first column denotes the row number from datS. The warning messages indicate that some of the factor levels for the sample traits specified by asfactors1 were excluded during linear regression modeling. These factor levels were excluded because they were wholly redundant with other factor levels already present in the model. For example, the exclusion of factor(sampleinfo2[indexgp2 - skip1, 6])c reflects the fact that the factor level "control" in the sample trait "Grade" (i.e. "c" in column 6 of datS) is identical to the designation of control ("C") in the sample trait "Dx" (diagnosis; column 5 in datS), and therefore contains no new information. The metrics reported below the warning messages are summaries of sample network statistics that can be used to gauge the overall quality and properties of the sample network. Finally, the user is also prompted to enter the row number(s) from the table of any outlying samples to remove, or to specify a sample connectivity threshold for removing outliers, which can be done by typing <-2 to remove all samples with Z.K less than –2. After the user indicates which outlying samples are to be removed, SampleNetwork regenerates the same outputs shown above after exclusion of these samples. This process is performed iteratively until the user indicates that there are no more outlying samples by typing None.

## As seen above and as discussed in the journal article, all of the samples with Z.K less than –2 are CN HD samples. Because HD preferentially targets destruction of CN relative to the other analyzed brain regions, it is plausible, if not likely, that the "outlying" status of these samples actually reflects a biological effect of interest, as opposed to a technical artefact. Furthermore, as shown in the bottom left panel of the figure above, brain region is exerts a strong effect on measured gene expression activity in this dataset. Therefore, rather than attempt to process this heterogeneous group of samples simultaneously, it makes more sense to process samples from each brain region separately. This can easily be done using SampleNetwork as follows:

## Hit the ESC key to escape the prompt. Then type:

indexCB = grep("CB", colnames(datE))

indexCN = grep("CN", colnames(datE))

indexBA4 = grep("BA4", colnames(datE))

indexBA9 = grep("BA9", colnames(datE))

## Where the grep command finds all columns that contain "CB" in the column headers of datE (i.e. all CB samples), etc. Then copy and paste the following command into the R session:

SampleNetwork(

datExprT=datE,

method1="correlation",

impute1=FALSE,

subset1=NULL,

skip1=2,

indices1=list(indexCB,indexCN,indexBA4,indexBA9),

subgroup1=5,

sampleinfo1=datS,

samplelabels1=3,

grouplabels1=7,

fitmodels1=TRUE,

whichmodel1="multivariate",

whichfit1="pc1",

btrait1=c(5,6,11,12,16,15),

trait1=c(6),

asfactors1=c(5,6,12,15,16),

projectname1="GSE3790",

cexlabels1=0.7,

normalize1=TRUE,

replacenegs1=FALSE,

exportfigures1=TRUE,

verbose=TRUE

)

## Note that a few parameters have changed. First, we are instructing SampleNetwork to process samples from CB, CN, BA4, and BA9 separately by providing a list of vectors that identify these sample columns in datE via the argument indices1. Instead of coloring samples according to brain region and diagnosis, we are now only going to color them by diagnosis (subgroup1=5), since we are processing each brain region separately. The group labels are now contained in the Region column from datS (grouplabels1=7); consequently, btrait1 and trait1 now exclude the Region column from linear modeling. Lastly, we will now test the significance of discrete grades of HD severity with respect to pc1 via trait1=c(6). The following output is produced:


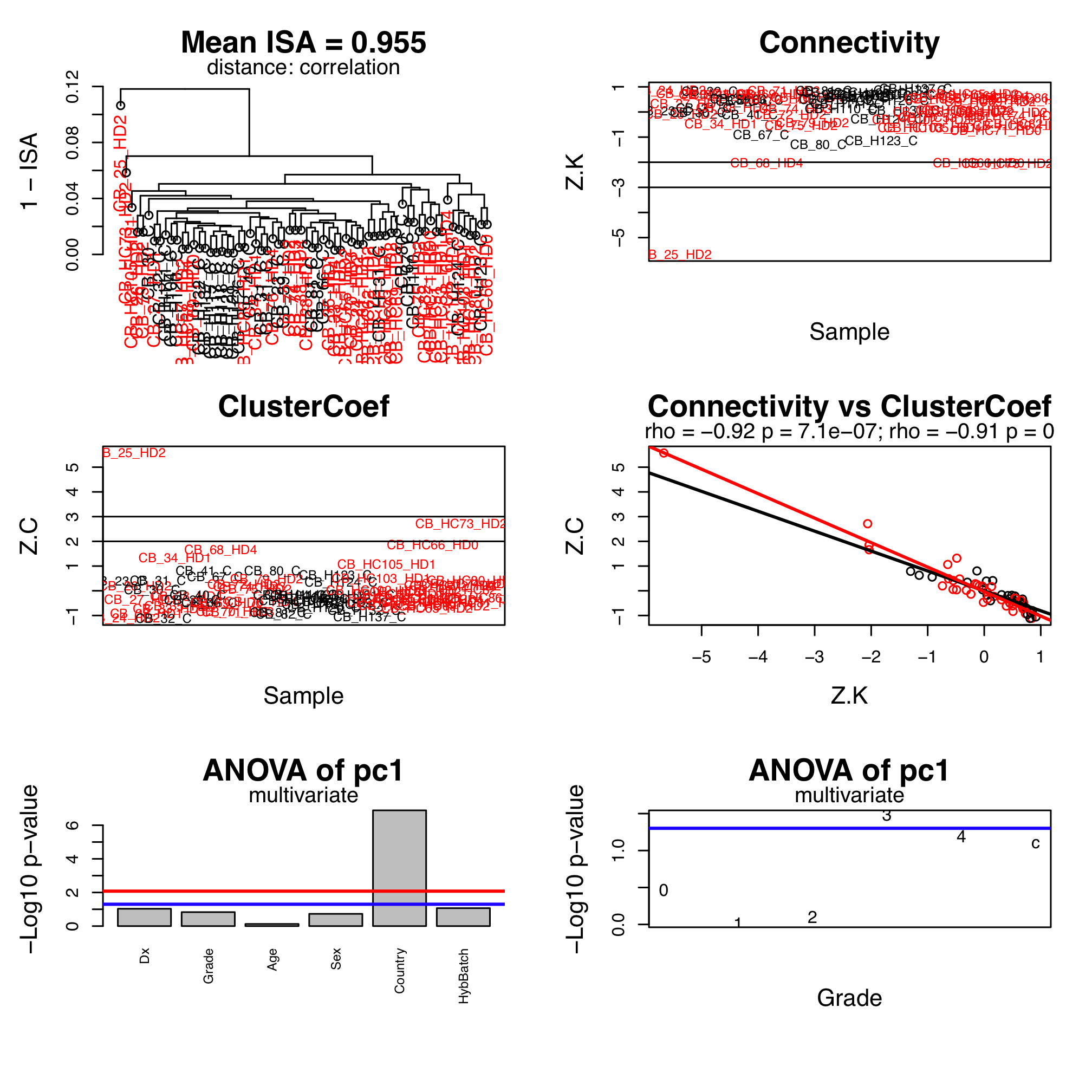


## Here we see the initial sample network constructed using only CB samples, with CTRL samples in black and HD samples in red. Overall, the mean ISA (intersample adjacency, or density), is 0.955, which we know from previous experience is reasonably good. In addition, initial cor(K,C) values for both CTRL and HD samples approach –1, which is a strong indicator of sample homogeneity (see journal article). In practice, when constructing correlation-based sample networks, high values for the mean ISA (e.g. > 0.95) in conjunction with cor(K,C) values approaching –1 indicate high correlations and high homogeneity among samples (i.e. clean data). We find that using these two indicators in tandem often provides useful guideposts for sample outlier detection and removal.

As can be seen in the top four panels, one sample is a clear outlier. To remove this sample, we can type:

Response: <-3

## Which will remove all samples with Z.K <-3 (in this case only one: CB_25_HD2, with Z.K = –5.67). After excluding this sample, the CB sample network is reconstructed as follows:


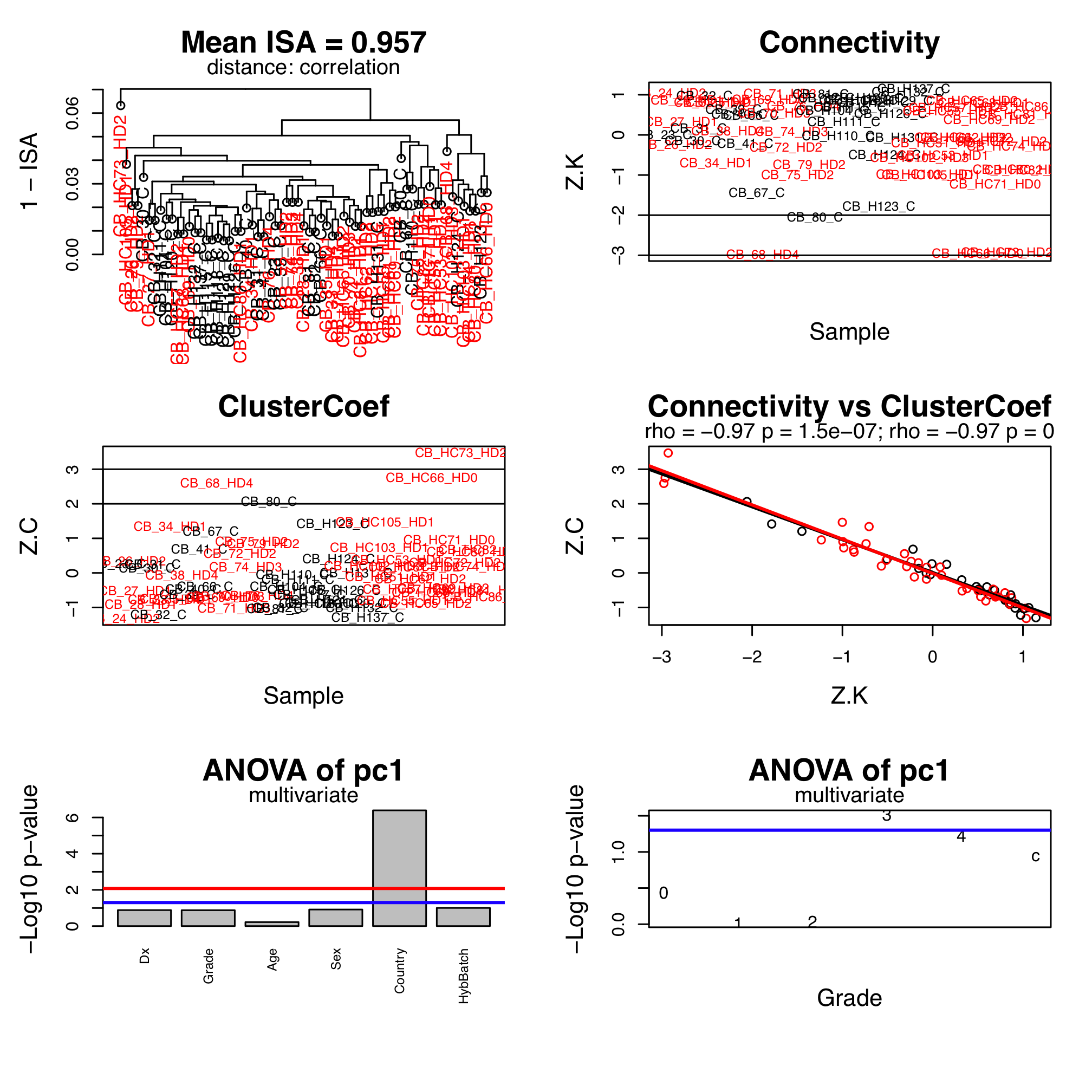


## Removal of this one sample has caused the mean ISA to increase to 0.957 and cor(K,C) to jump to –0.97 for both CTRL and HD samples (middle right panel). However, several additional CB samples look substantially lower than the rest. To remove these:

Response: <-2.5

## Which will remove the three samples with Z.K <-2.5. Rebuilding:


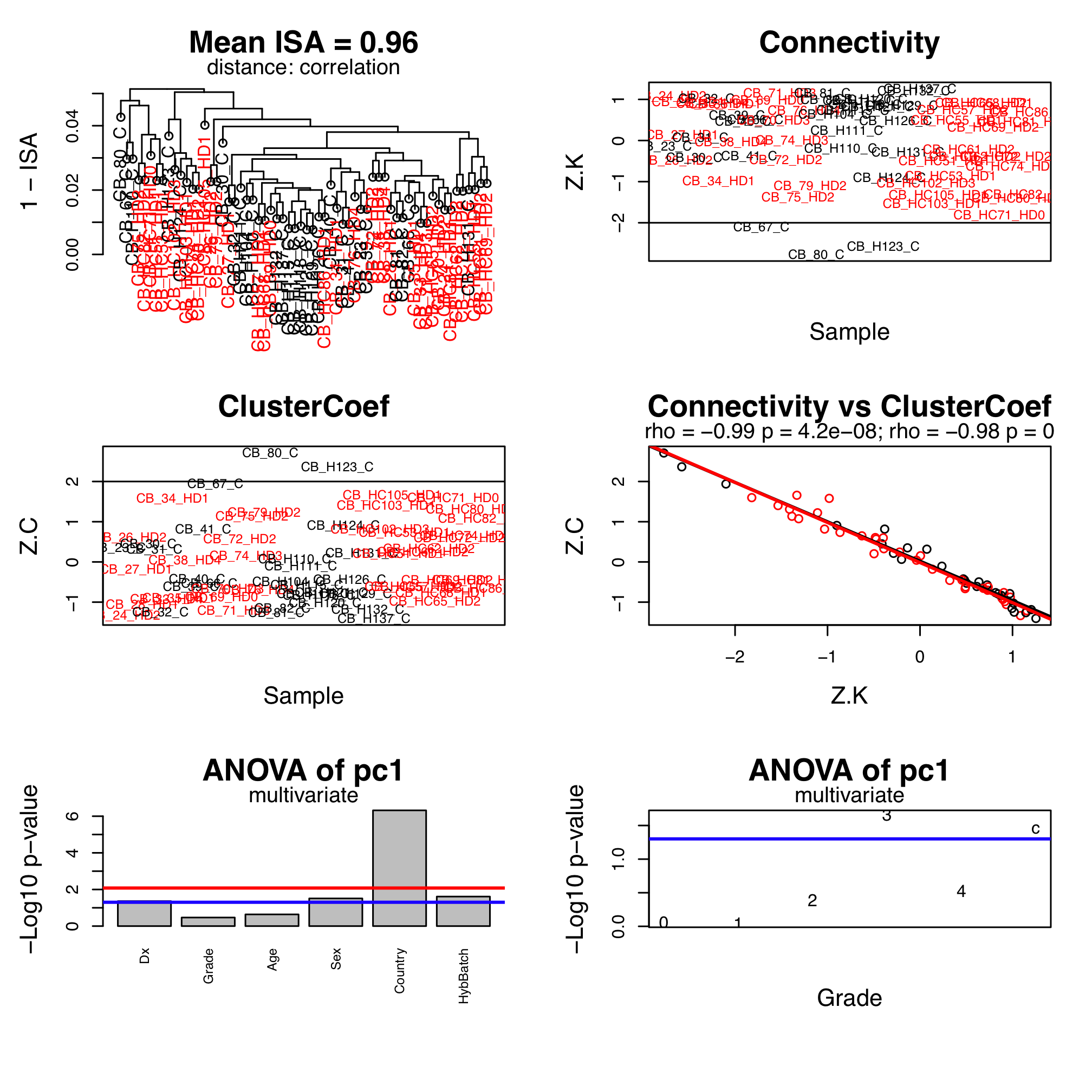


## We note that the choice of Z.K threshold for removing outlying samples is up to the user. In general, this decision can be guided by the overall sample homogeneity in the dataset (i.e. mean ISA and cor(K,C)), the number of samples, and the distribution of Z.K values. It is good to start with the obvious outliers (e.g. Z.K < –3), prune the dataset iteratively until no samples fall outside of this initial range, then reassess. The distribution of samples shown above reveals three with Z.K < –2 that are substantially lower than the rest. Removing these and rebuilding:

Response: <-2


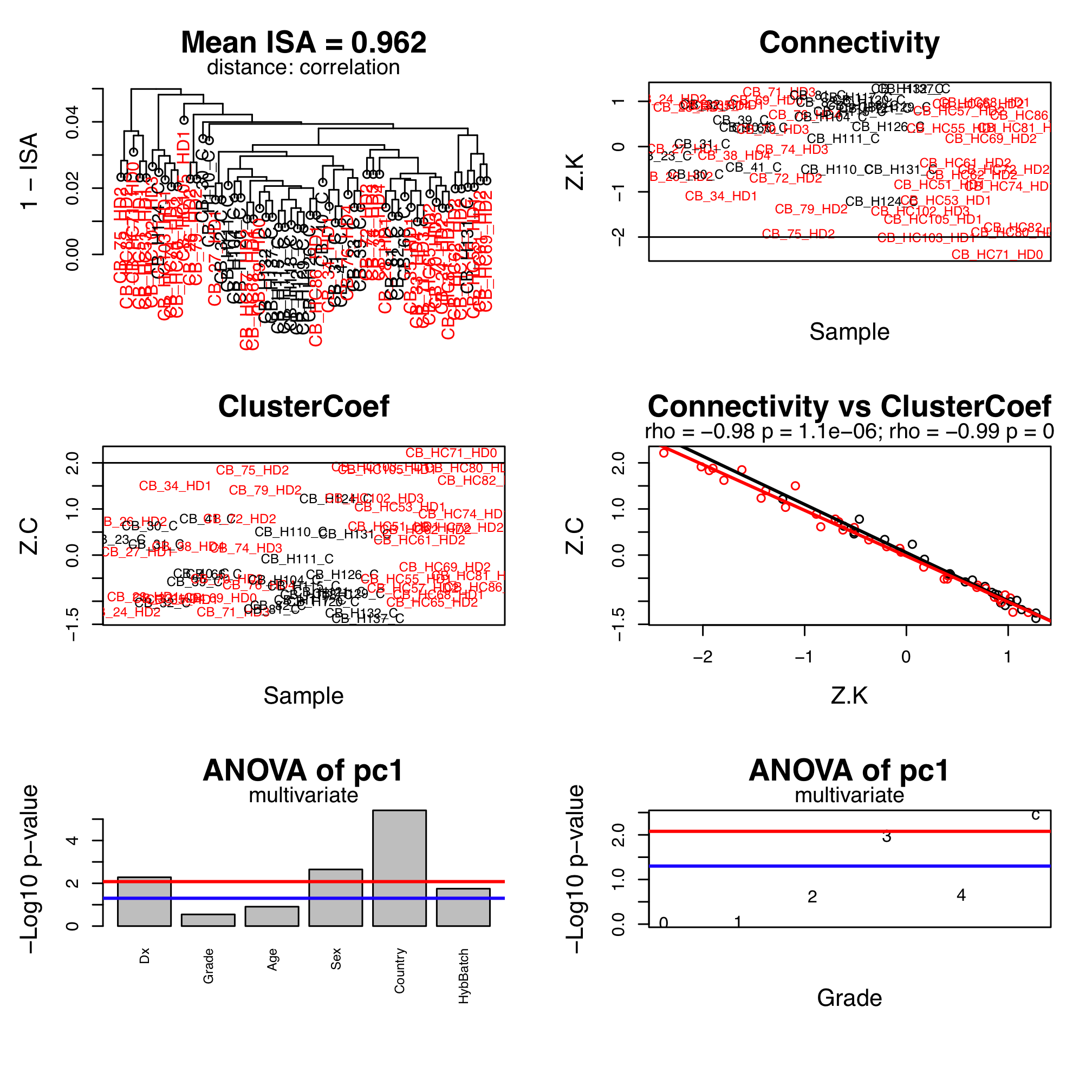


## We now see only one sample with Z.K ≅ –2.3 that is below the rest. We will remove this sample by row number and rebuild:

Response: 59


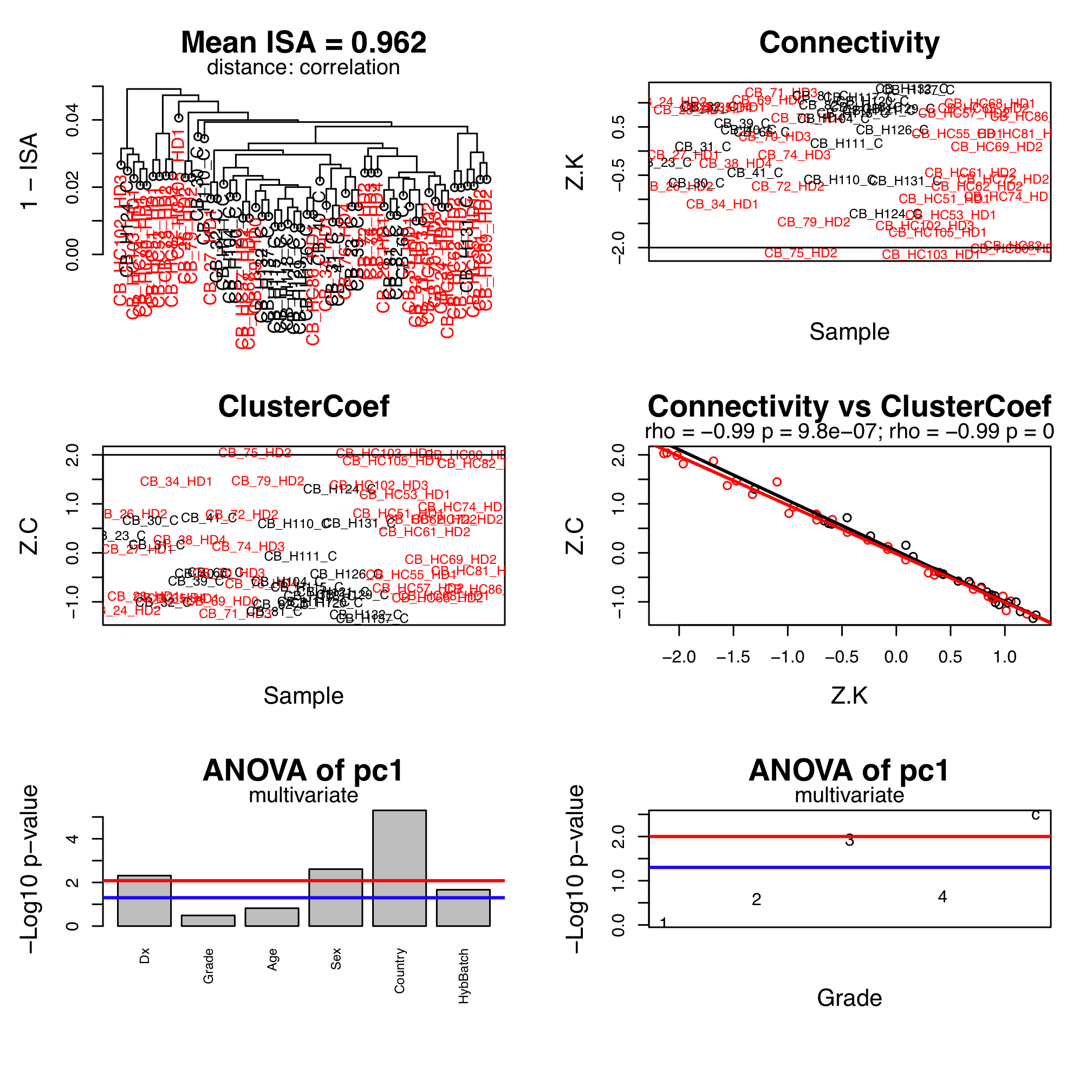


## At this point, we have removed 8 / 66 or 12.1% of CB samples (SampleNetwork keeps a running tally of the cumulative number / percentage of samples removed after each round of processing, which is reported in the R session). The mean ISA is now 0.963, cor(K,C) ≅ –1 for both CTRL and HD samples, and the distribution of Z.K values is now much tighter. However, careful inspection reveals that one CTRL sample (CB_H124_C) is clustering with the HD samples in the top left panel, and has Z.K significantly lower than all of the other CTRL samples (top right). Therefore, we will remove this sample by row number and rebuild:

Response: 40


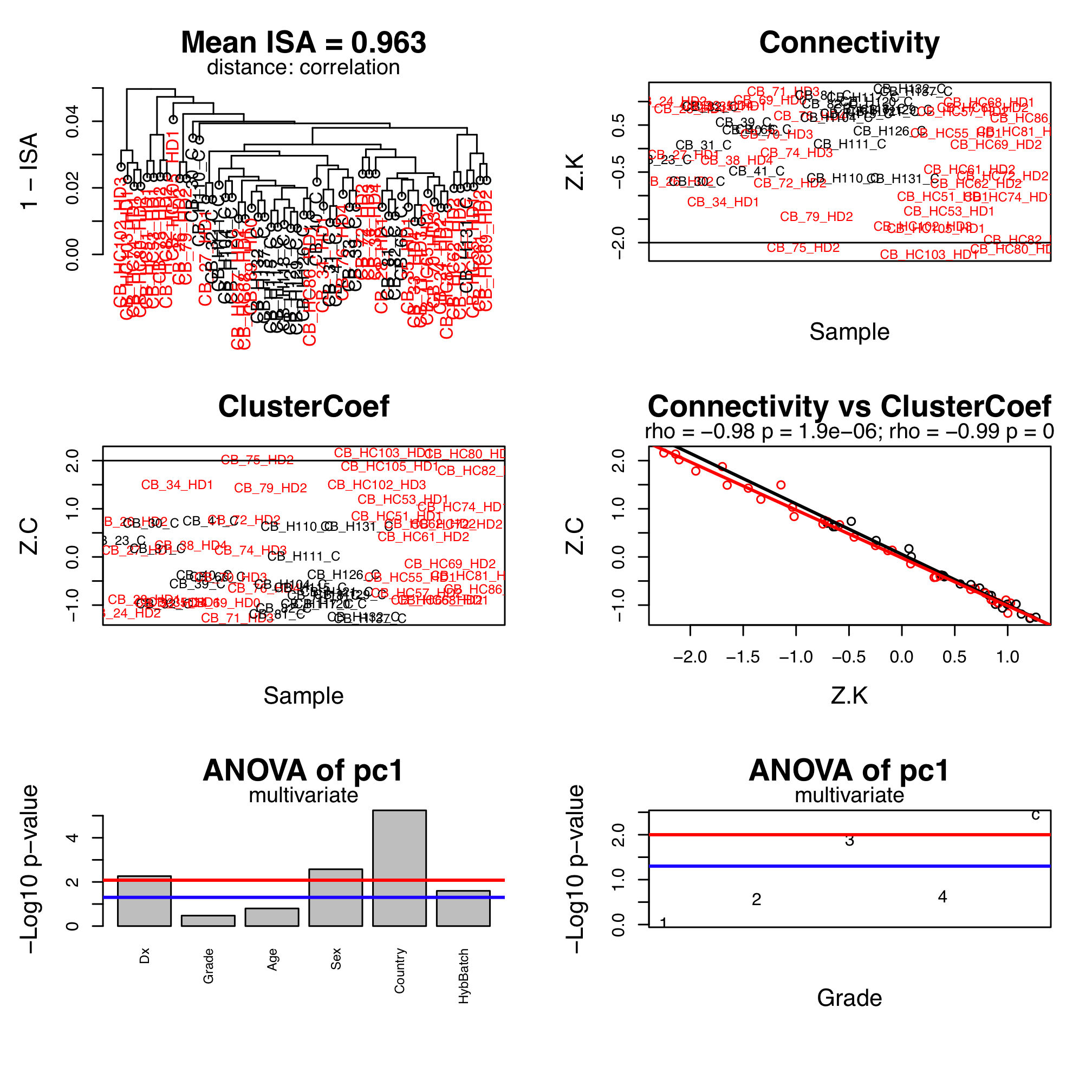


## Since there are no obvious outliers left, we will type None at the prompt. Because the argument normalize1 = TRUE, SampleNetwork will automatically perform quantile normalization and rebuild the sample network. Note that prior to quantile normalization, the sample trait "Country" (i.e. the country where samples were processed [US or NZ]) shows a significant association with pc1 (along with Diagnosis [Dx] and Sex).

Response: None


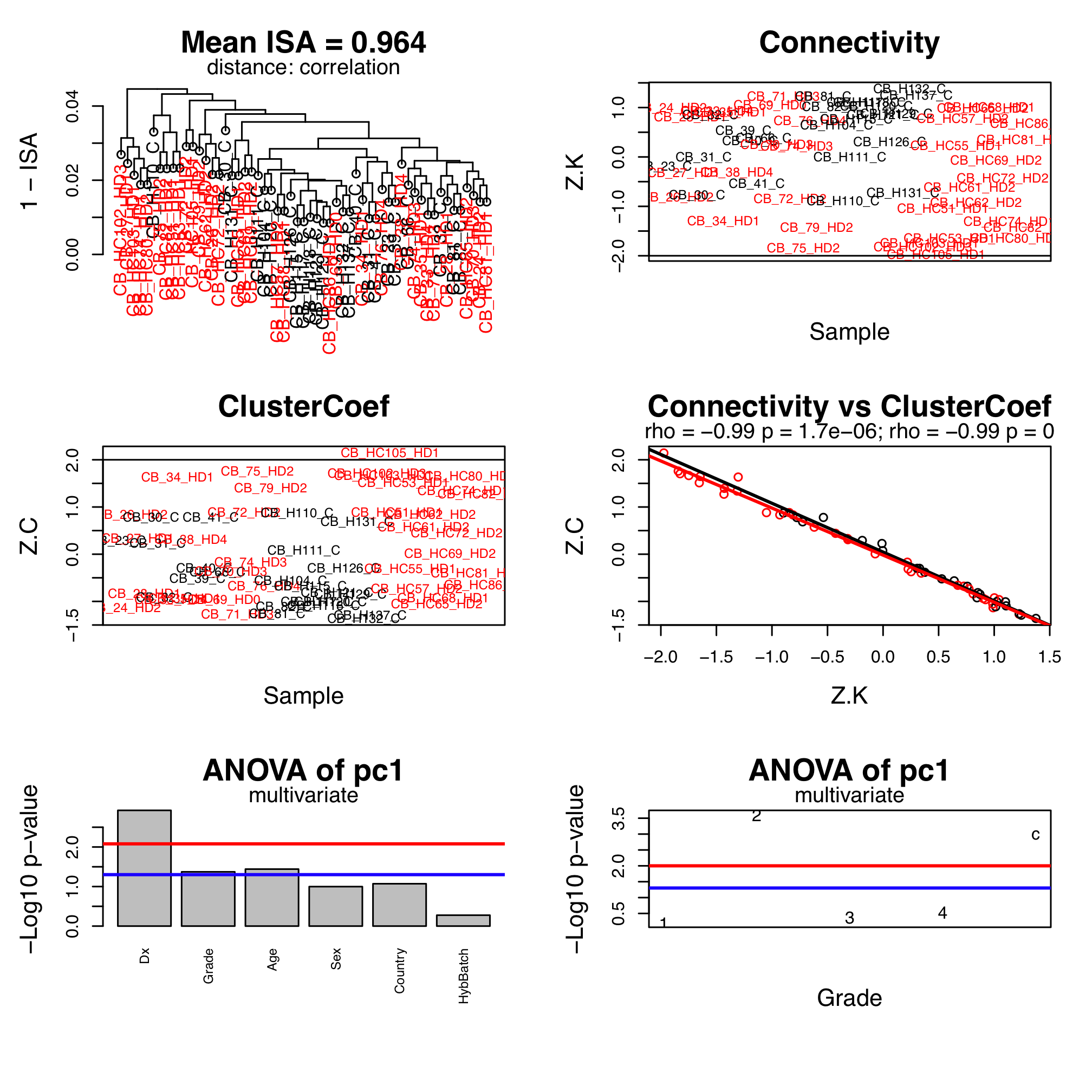


## However, as seen in the bottom left panel above, quantile normalization has in fact mostly corrected for the effect of Country on pc1, such that it is no longer significant. Note that Dx does exert a significant effect with respect to pc1, which suggests that subtle effects of HD on CB gene expression may be present. The user is now prompted to perform batch correction:

[1] Please enter the row number from the output below that you would like to use for batch normalization or type 'None'

Sample info

1 Region

2 Label

3 Dx

4 Grade

5 Age

6 Sex

7 Country

8 HybBatch

9 K

10 Z.K

11 Z.C

12 Z.MAR

## Because batch correction is not necessary in this case, we will type "None":

Response: None

## At this point, the user is informed that:

[1] "CB processing complete. Hit enter to proceed to next group."

## Doing so moves on to the next batch of samples: CN. The initial sample network is shown below:


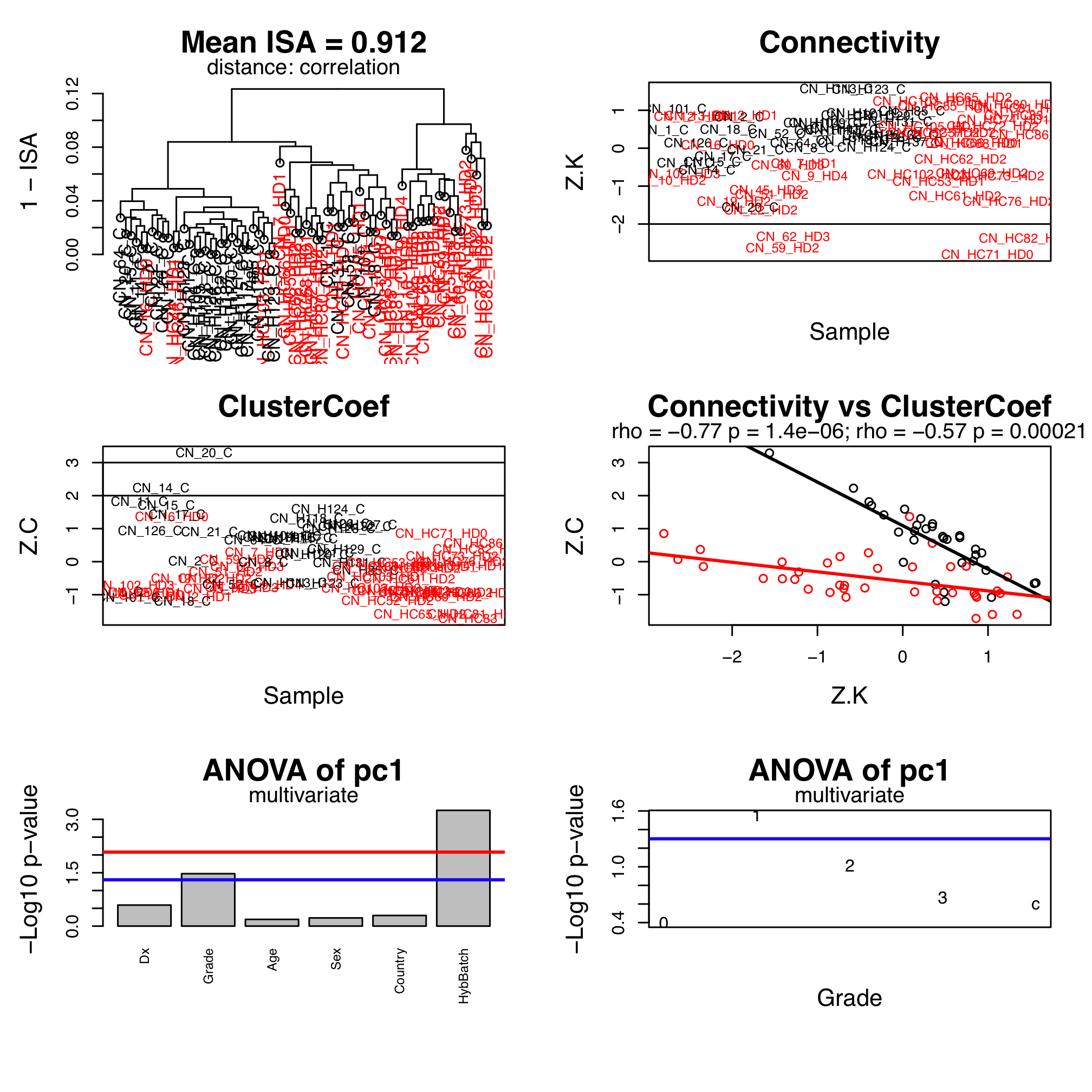


## Here it can be seen that the mean ISA is much lower, and there is a clear separation between CTRL and HD samples in the cor(K,C) plot (as discussed in the journal article). In addition, while Country does not show a significant effect with respect to pc1, HybBatch does. Paradoxically, Dx does not initially appear significant with respect to pc1. Because we expect CN to show the most significant association with Dx, this result indicates that there is likely a strong need for additional data preprocessing. However, before addressing batch effects, we need to decide whether to remove any outlying samples. This problem is complicated by the fact that CN is the neuroanatomical epicenter of HD, and therefore we must try to dissociate between non-biological (i.e. technical) effects and effects caused by HD. To help us with this task, we can make use of the available biological information, which includes the grade of HD severity. Three of the four HD samples with Z.K < -2 are grade 2 or grade 3 HD (i.e. severe pathology). However, the sample with the lowest Z.K is grade 0 (i.e. very minimal pathology). Therefore, it seems unlikely that this sample's outlying status would be solely attributable to HD, and to be conservative, we will remove it by row number and rebuild:

Response: 127


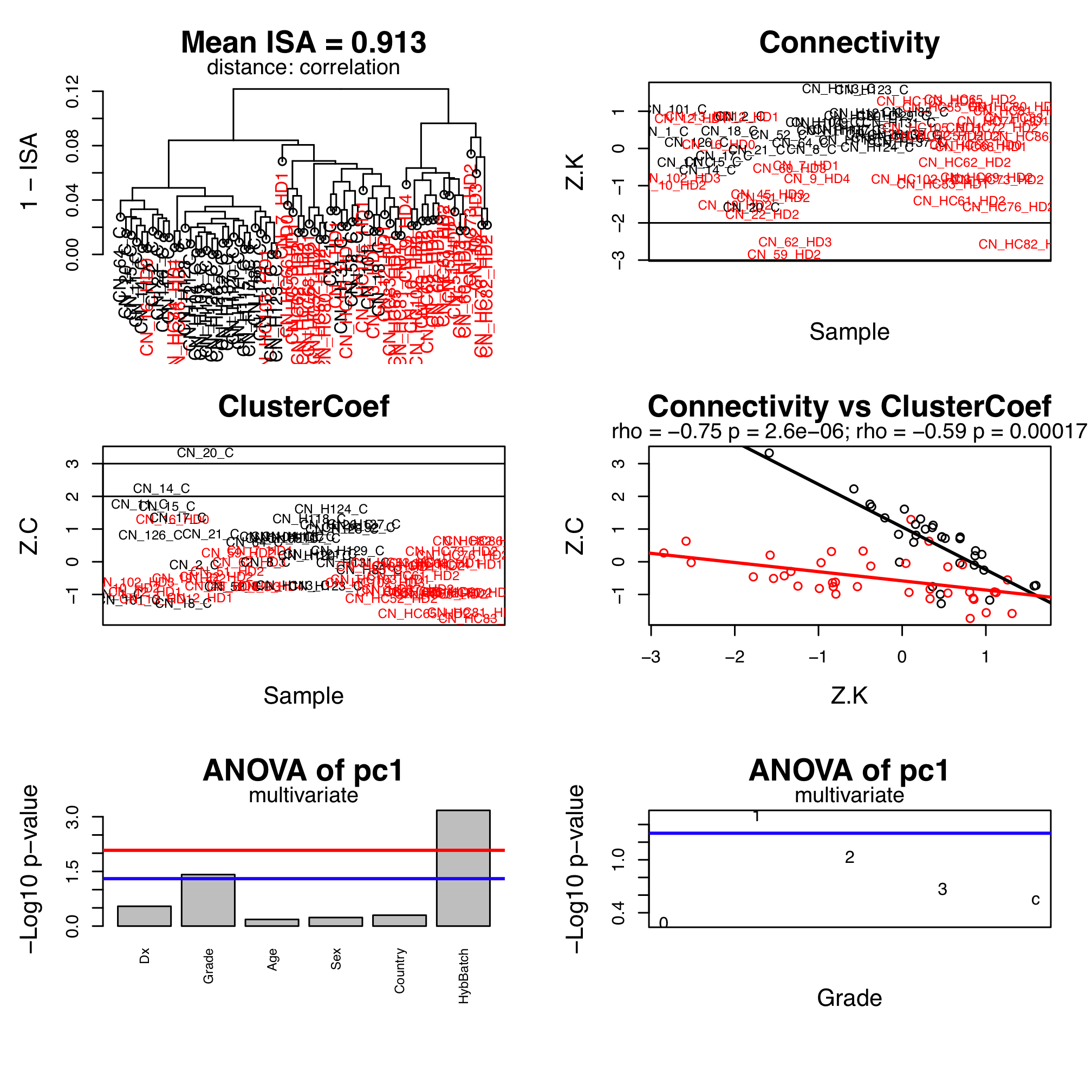


## We also see that there is one control sample with Z.K that is substantially lower than all of the other control samples: CN_20_C. Relative to its peers, this sample is also an outlier, so we will remove it by row number and rebuild:

Response: 83


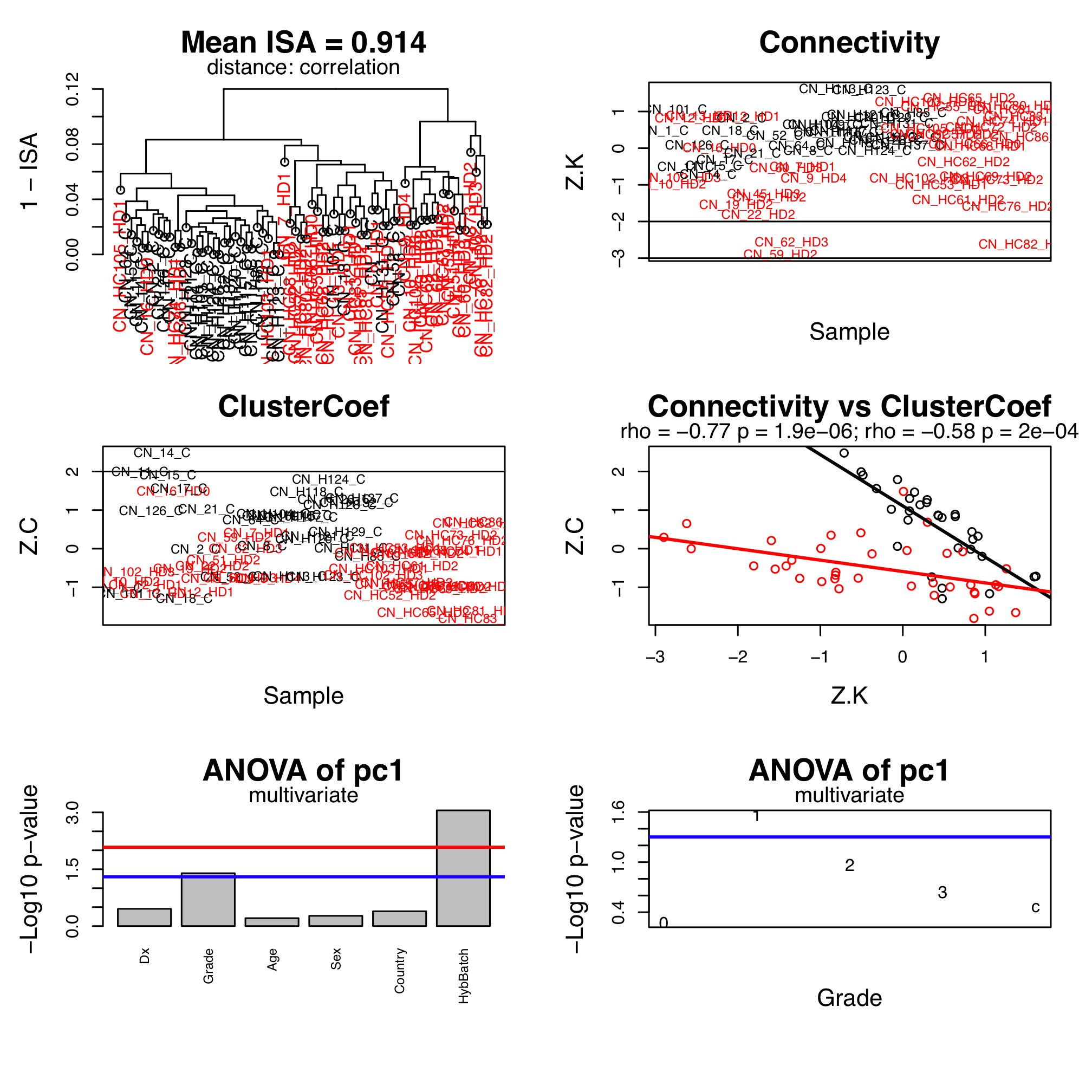


## One may also make use of the clustering dendrogram, when practical. For example, on the far left of the dendrogram in the top left panel, there is one HD sample that exhibits divergent clustering (CN_HC105_HD1) from a group comprised almost exclusively of CTRL samples. Targeting this sample by row number:

Response: 116


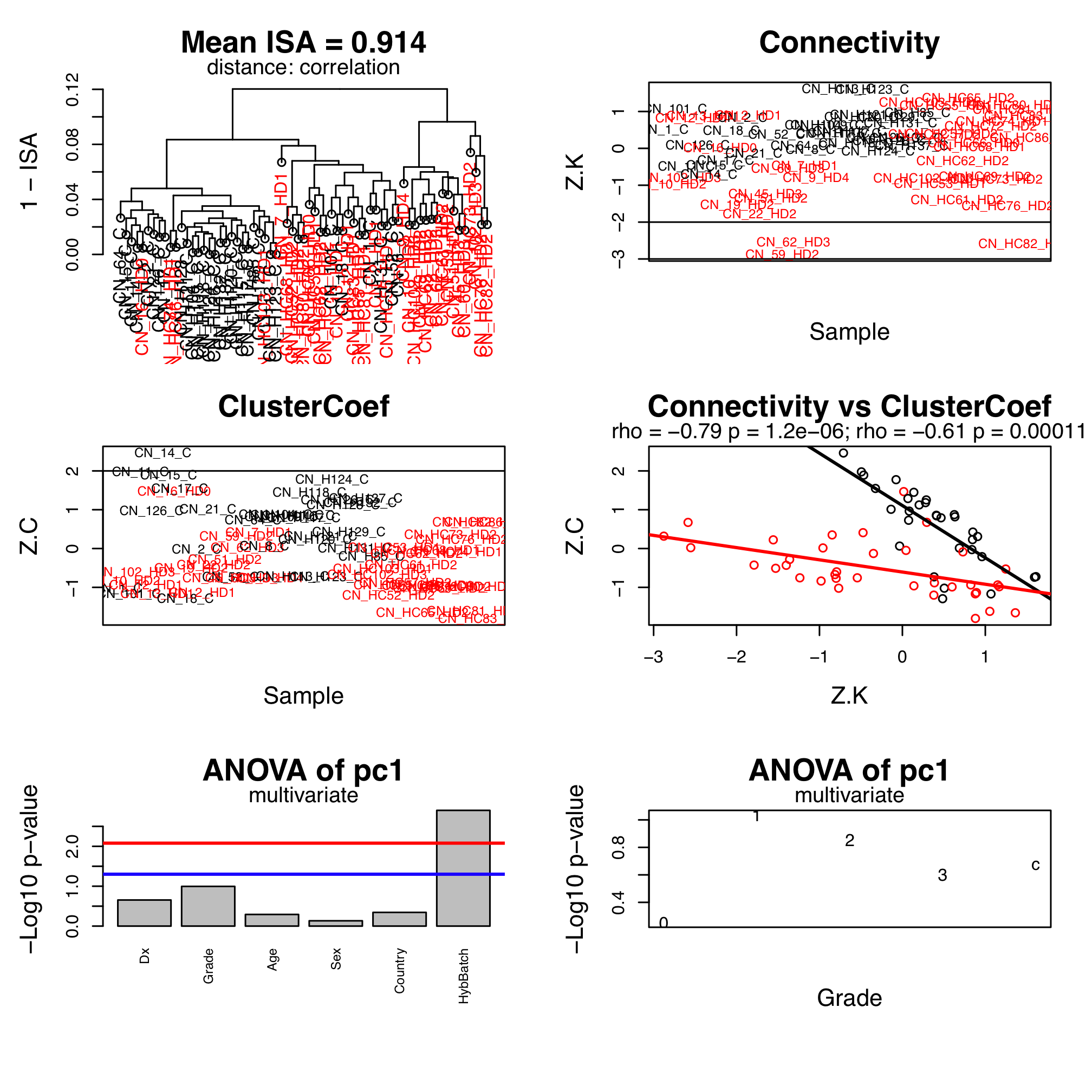


## There is one other HD sample that exhibits divergent clustering (CN_7_HD1), which we will also target by row number:

Response: 93


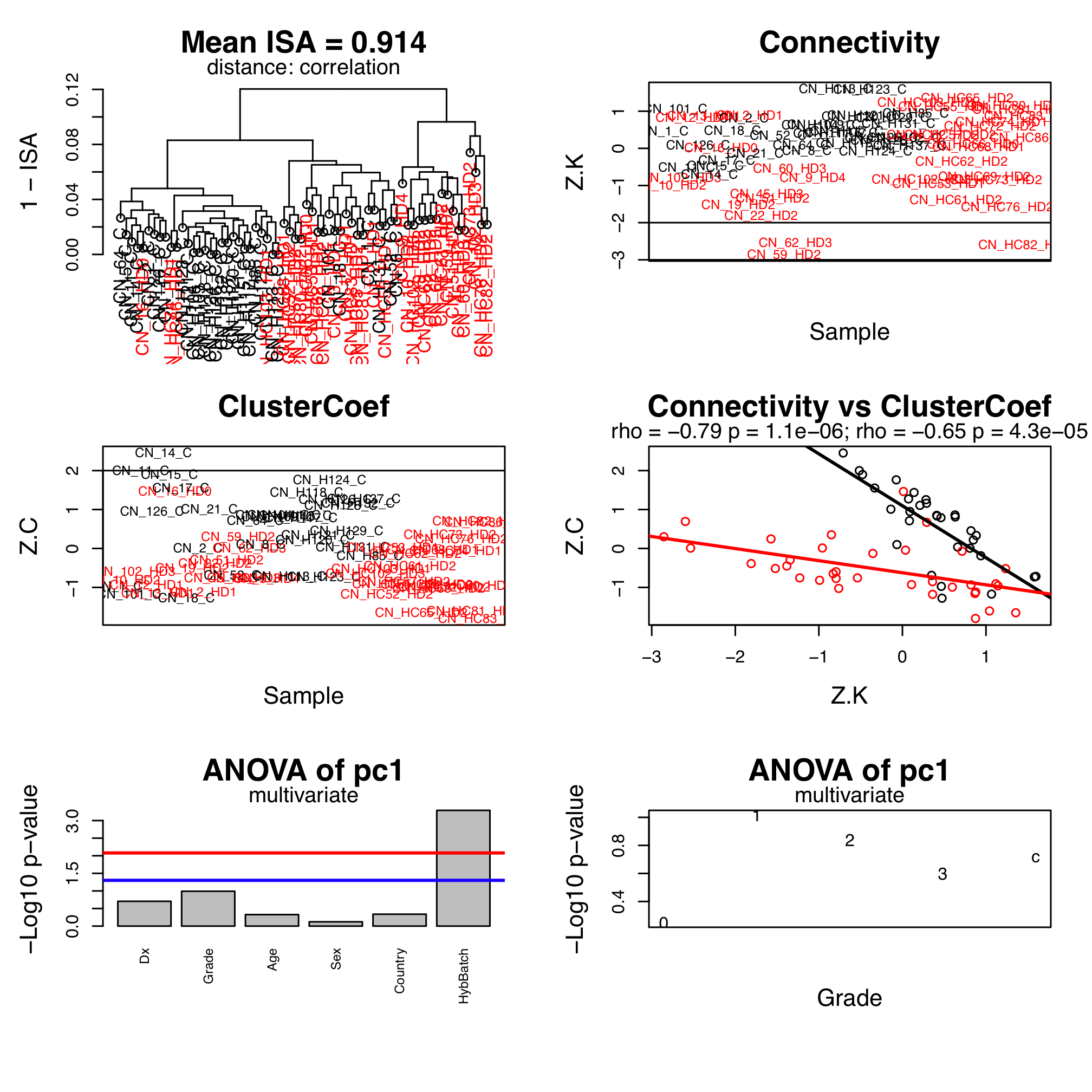


## We will now proceed to quantile normalization:

Response: None


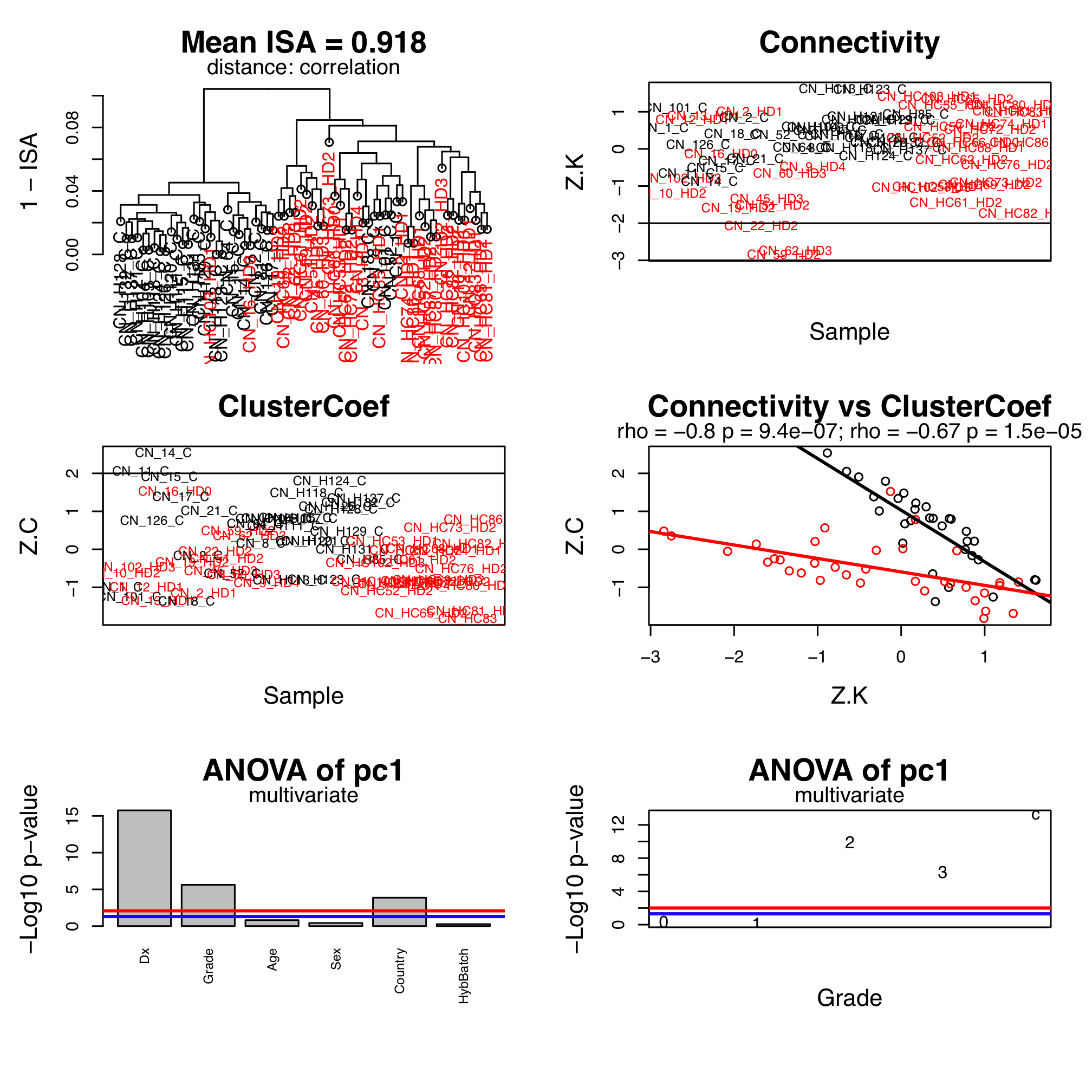


## In this case, quantile normalization corrects for the technical effect of HybBatch, while also revealing the highly significant effect of Dx on gene expression in CN. However, we now also see a significant effect of Country on pc1 (though not as significant as Dx or Grade, which is revealed for discrete levels in the bottom right panel). Therefore, we will run ComBat [2] to remove the effect of Country on pc1 of CN samples. SampleNetwork prompts us to:

[1] Please enter the row number from the output below that you would like to use for batch normalization or type 'None'

Sample info

1 Region

2 Label

3 Dx

4 Grade

5 Age

6 Sex

7 Country

8 HybBatch

9 K

10 Z.K

11 Z.C

12 Z.MAR

Response: 7

## We entered 7 indicating that we want to correct for the effect of Country. Note that one can in principle correct for any sample trait (assuming that it is a categorical variable). We are now asked to confirm our choice:

[1] "Country will be used. Is this correct? Type y/n."

Response: y

## We type 'y' and are asked which batch(es) to correct:

[1] Please indicate which batch(es) you would like to correct (comma separated), or type 'All' for all batches. If no batch correction is needed, type 'None'.

Response: All

## We type 'All' to correct all batches in the sample trait Country (in this case, just US and NZ). In the event that only one level of a batch or a subset of batch levels is deemed significant (for example, as illustrated in the bottom right panel for Grade), it is up to the user to decide how the batch normalization should be performed. For example, imagine that there is one batch among six that has a strong influence on gene expression. If the user were to employ SampleNetwork to correct only for this batch, then a total of two batches would be passed to ComBat (one for samples from the "bad" batch, and one for all of the other samples). Alternatively, the user could pass the entire batch structure to ComBat (i.e. all six), even though only one batch exerted a strong influence on activity levels. The relative merits of each approach deserve further study. However, in general, we find that it may be better to correct for all batches. We are now prompted as to which sample traits, if any, we would like ComBat to use as covariates during batch correction. In general, the authors of ComBat recommend that sample traits that are expected to exert a significant influence on gene expression values should be included as covariates during batch correction [2]. Here, we include Dx and Sex.

[1] Please enter the row number(s) from the output below that you would like to use as covariates during batch normalization or type 'None'

[1] Note: ComBat will only work with categorical covariates

Sample info

1 Array

2 Sample

3 Label

4 Platform

5 Dx

6 Grade

7 Region

8 Genotype

9 GenNum

10 GenDenom

11 Age

12 Sex

13 Individual

14 HybDate

15 HybBatch

16 Country

17 Dx_Region

18 All

Response: 5,12

## We are prompted:

[1] Dx & Sex will be used. Is this correct? Type y/n.

Response: y

## After typing 'y', SampleNetwork generates a required sample information file for ComBat and calls the ComBat function automatically. Following batch correction via ComBat, the sample network is rebuilt:


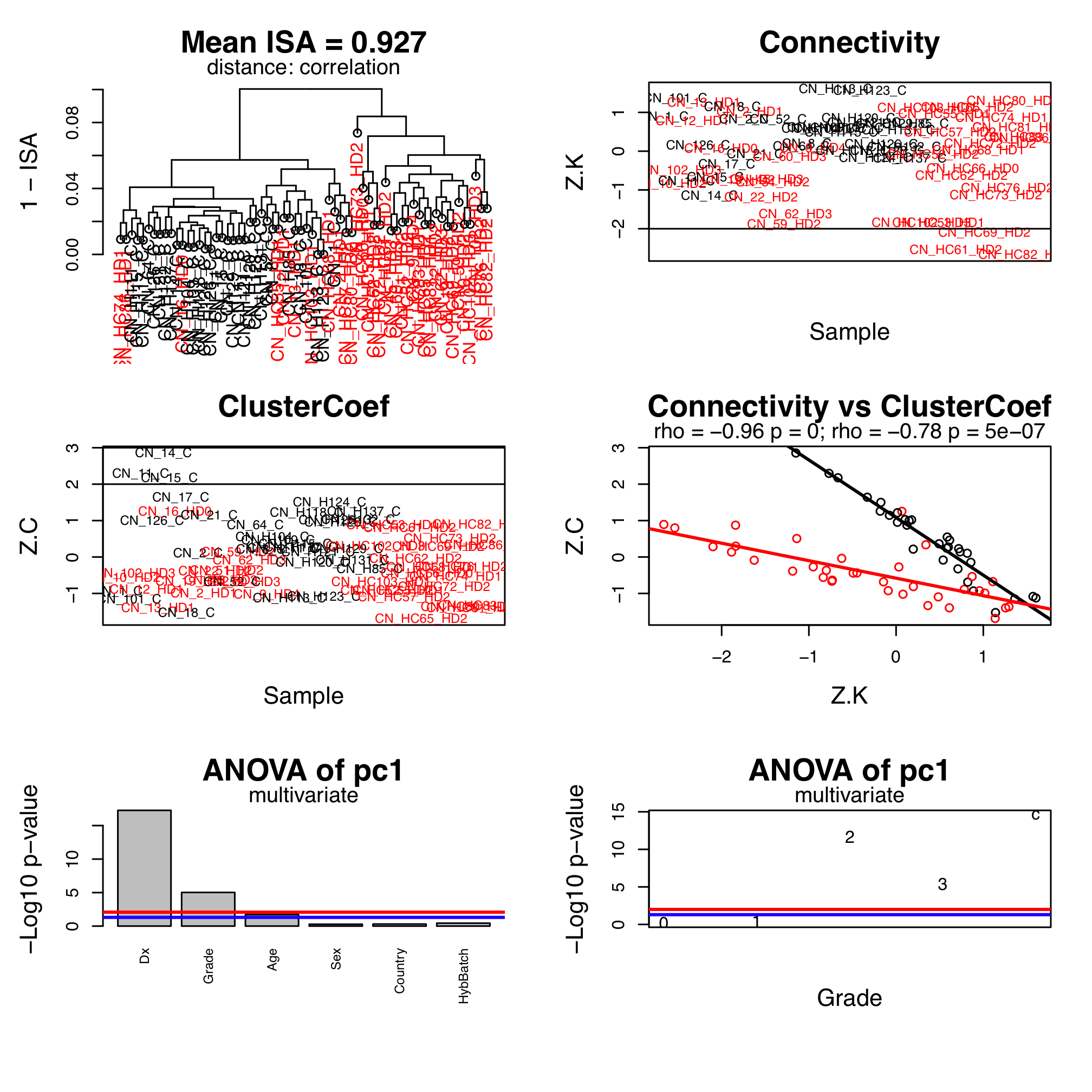


## We can see that Country no longer exerts a significant effect on pc1, whereas the strong effect of Dx is preserved. Thus, ComBat has succeeded in removing the batch effect. We are now informed:

[1] "CN processing complete. Hit enter to proceed to next group."

Press enter:

## After hitting enter, we see the initial sample network for BA4 (motor cortex):


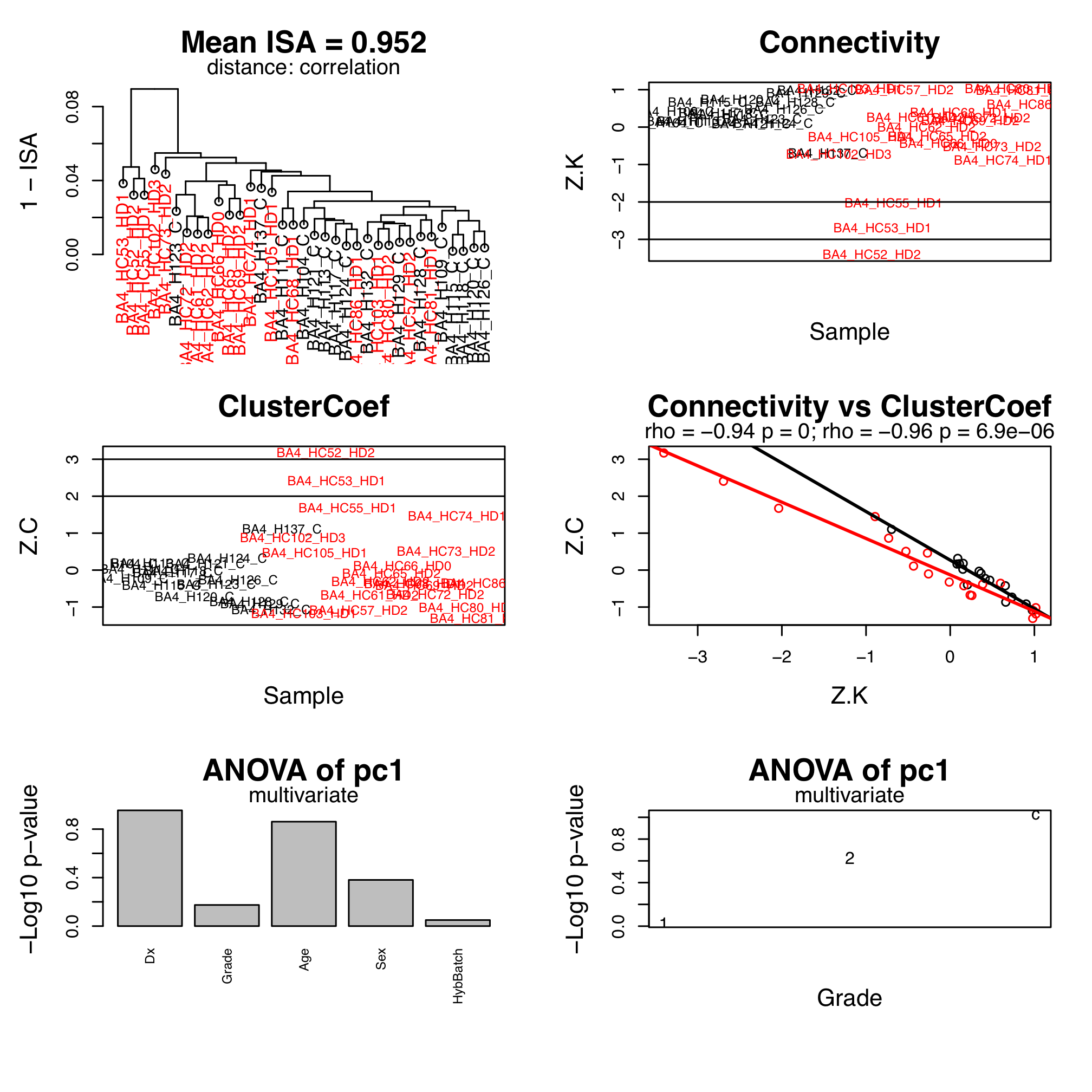


## The mean ISA is much higher than it was in CN, and initial cor(K,C) values already approach –1. However, three samples are clear outliers. Removing these:

Response: <-2


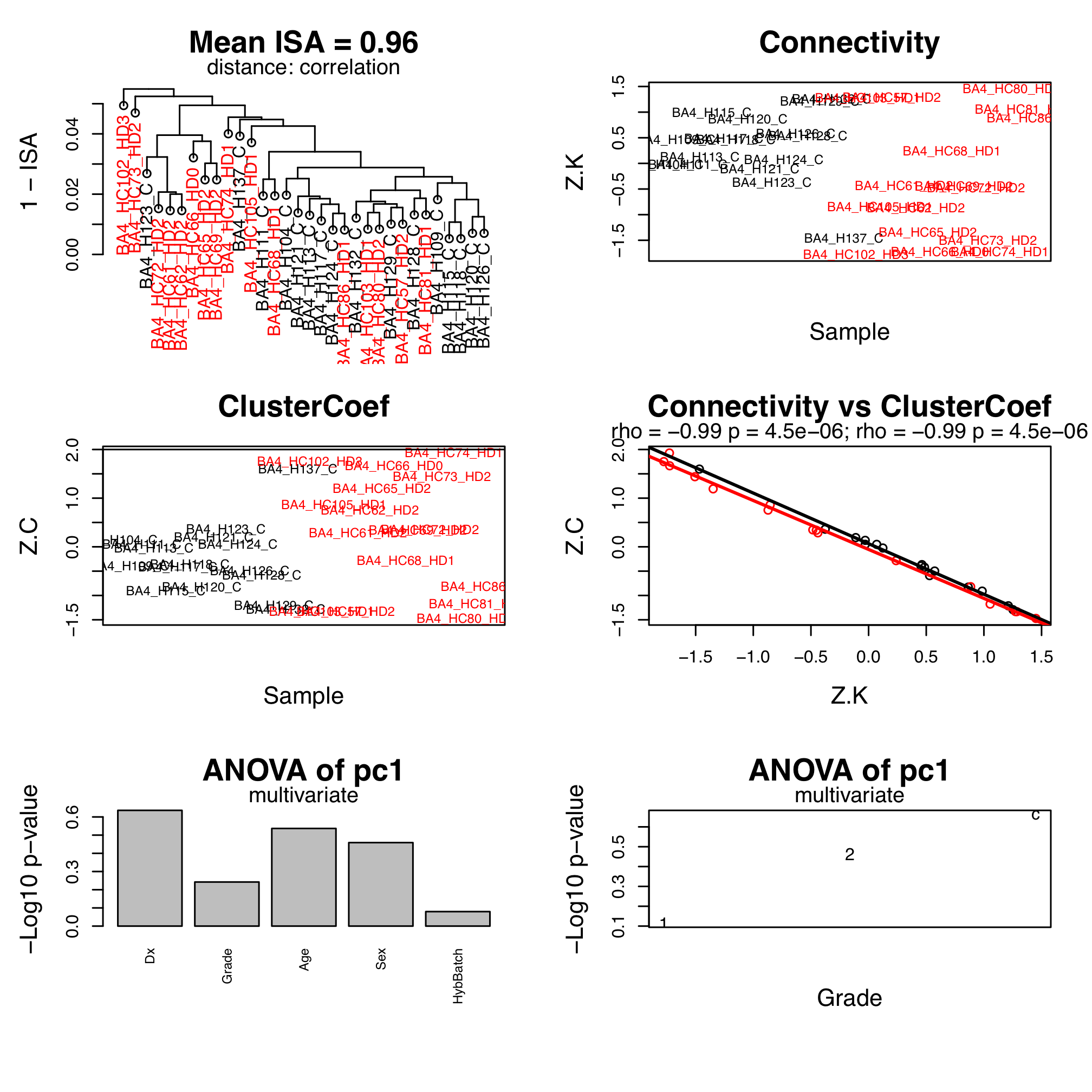


## Although all samples now have Z.K < –2, two samples exhibit divergent clustering on the far left of the dendrogram (top left panel). To be conservative, we will remove the most divergent sample (BA4_HC102_HD3) by row number and see what that does to the dendrogram:

Response: 153


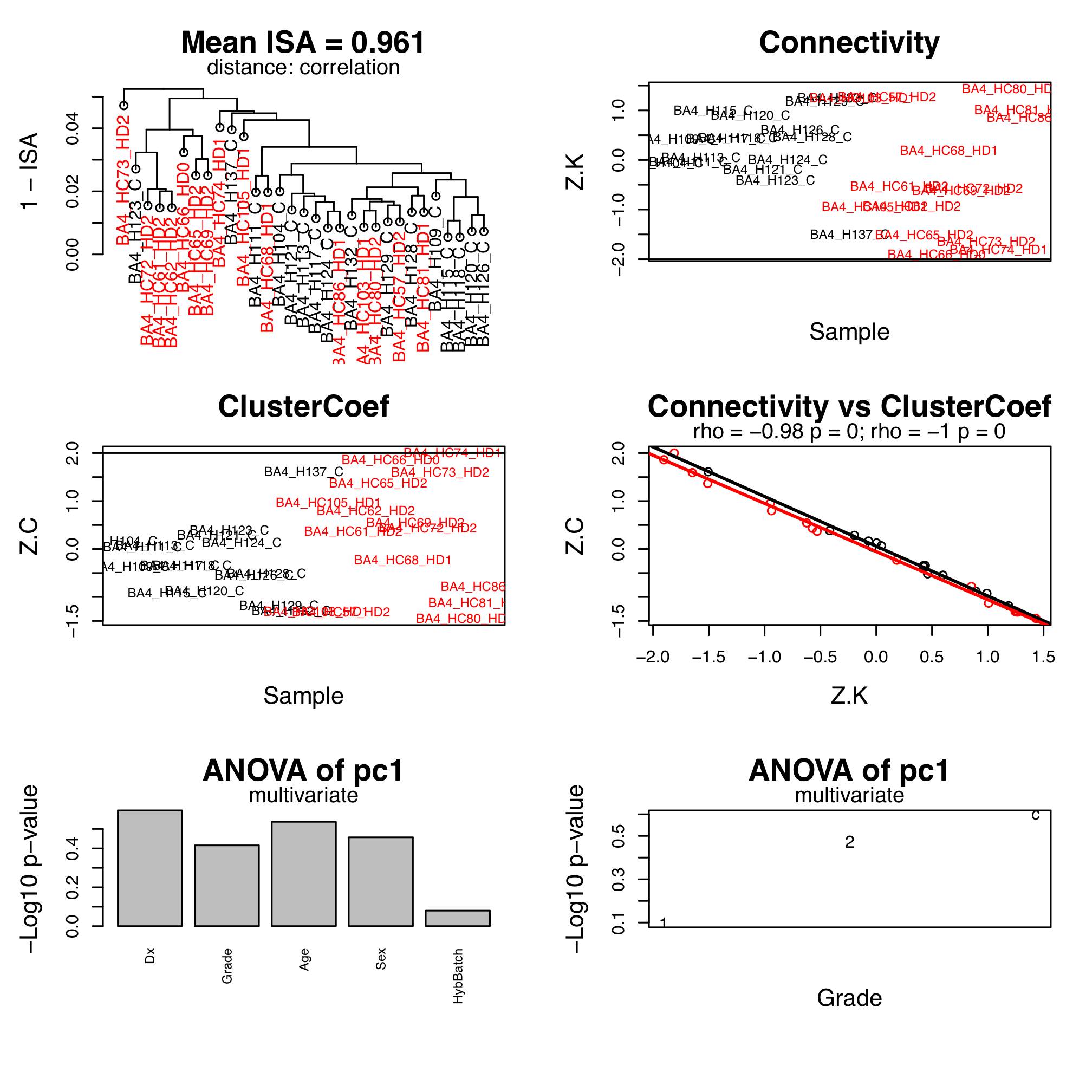


## Now we will remove the other sample (BA4_HC73_HD2) by row number:

Response: 167


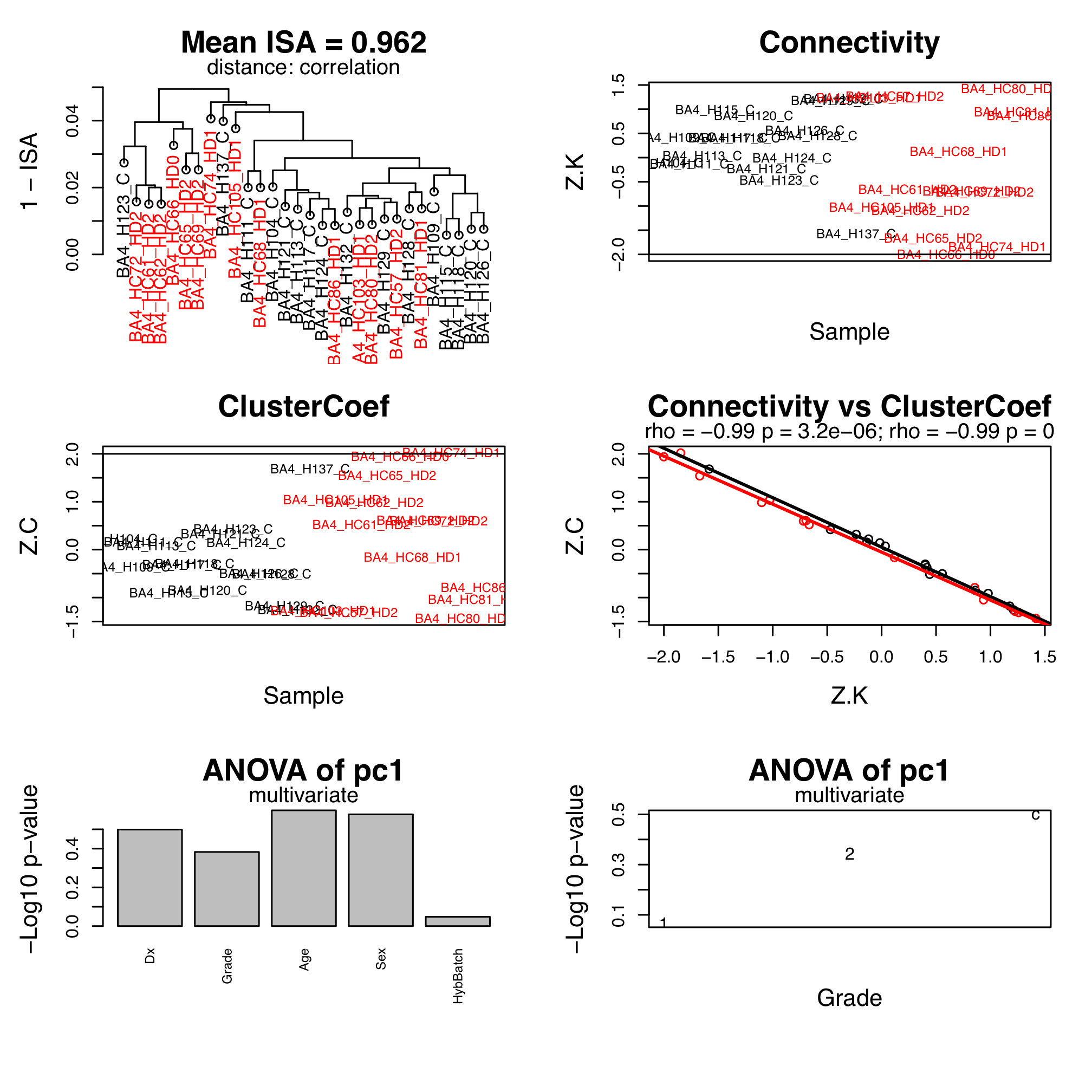


## Now we will perform quantile normalization:

Response: None


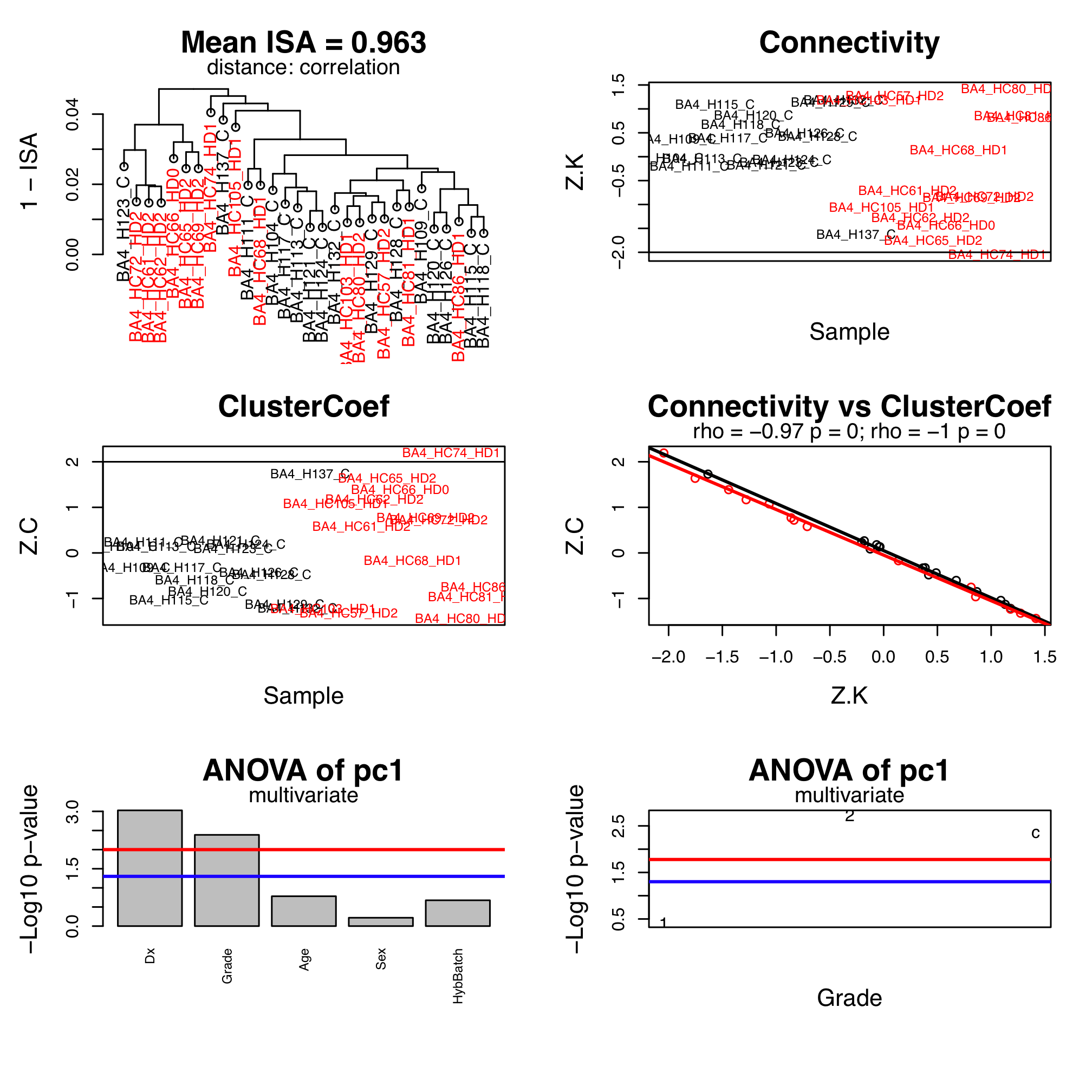


## Note that there is now a significant effect of Dx and Grade on pc1 (though not nearly as significant as in CN). There is no batch effect associated with HybBatch for these samples (and all samples from BA4 were processed in the same country), so we will decline batch normalization:

Response: None

## We are now informed:

[1] "BA4 processing complete. Hit enter to proceed to next group."

Press enter:

## After hitting enter, we see the initial sample network for BA9 (prefrontal cortex):


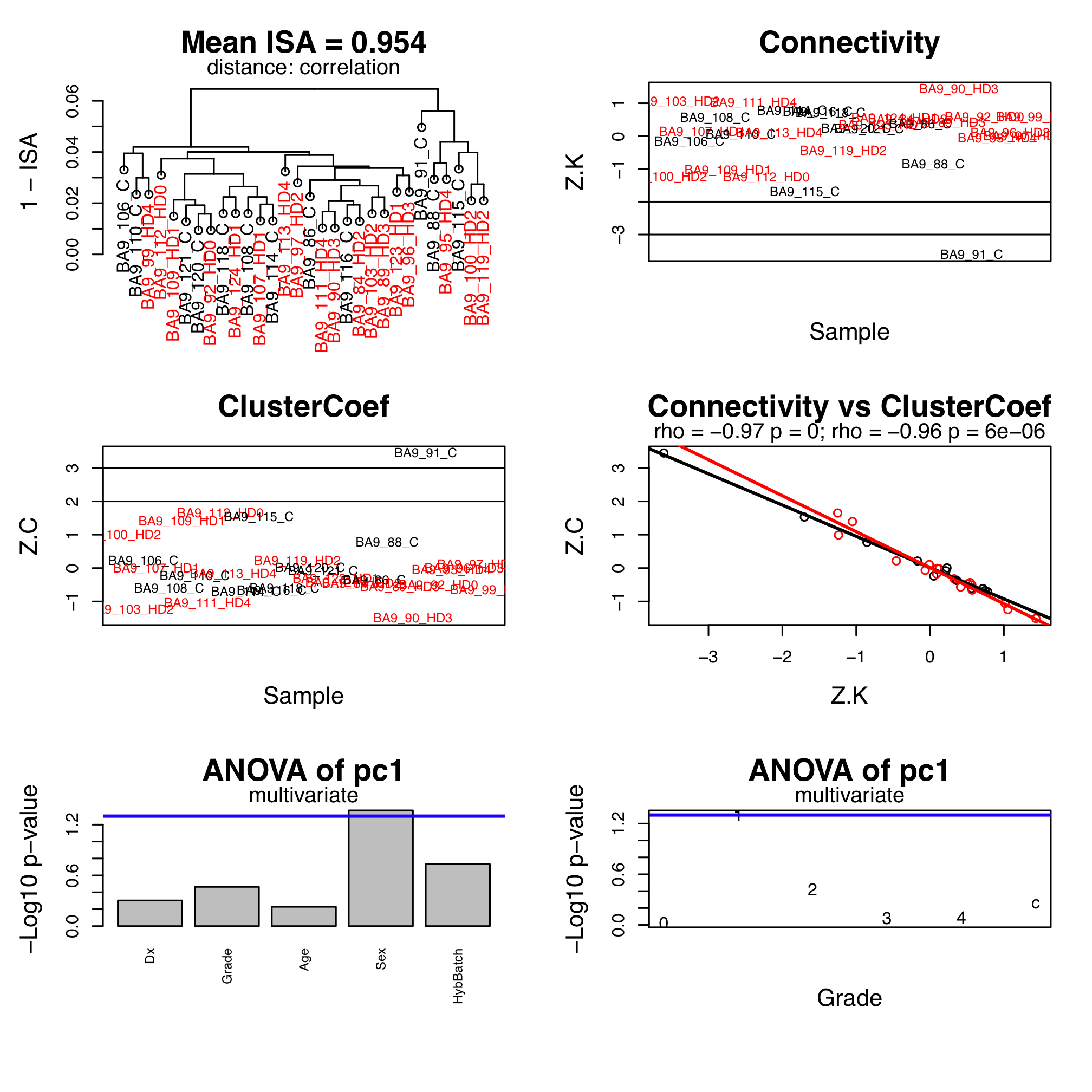


## Looking at the initial sample network for BA9, we can see that things look pretty good to start, though there is one clear outlier. Removing this sample:

Response: <-3


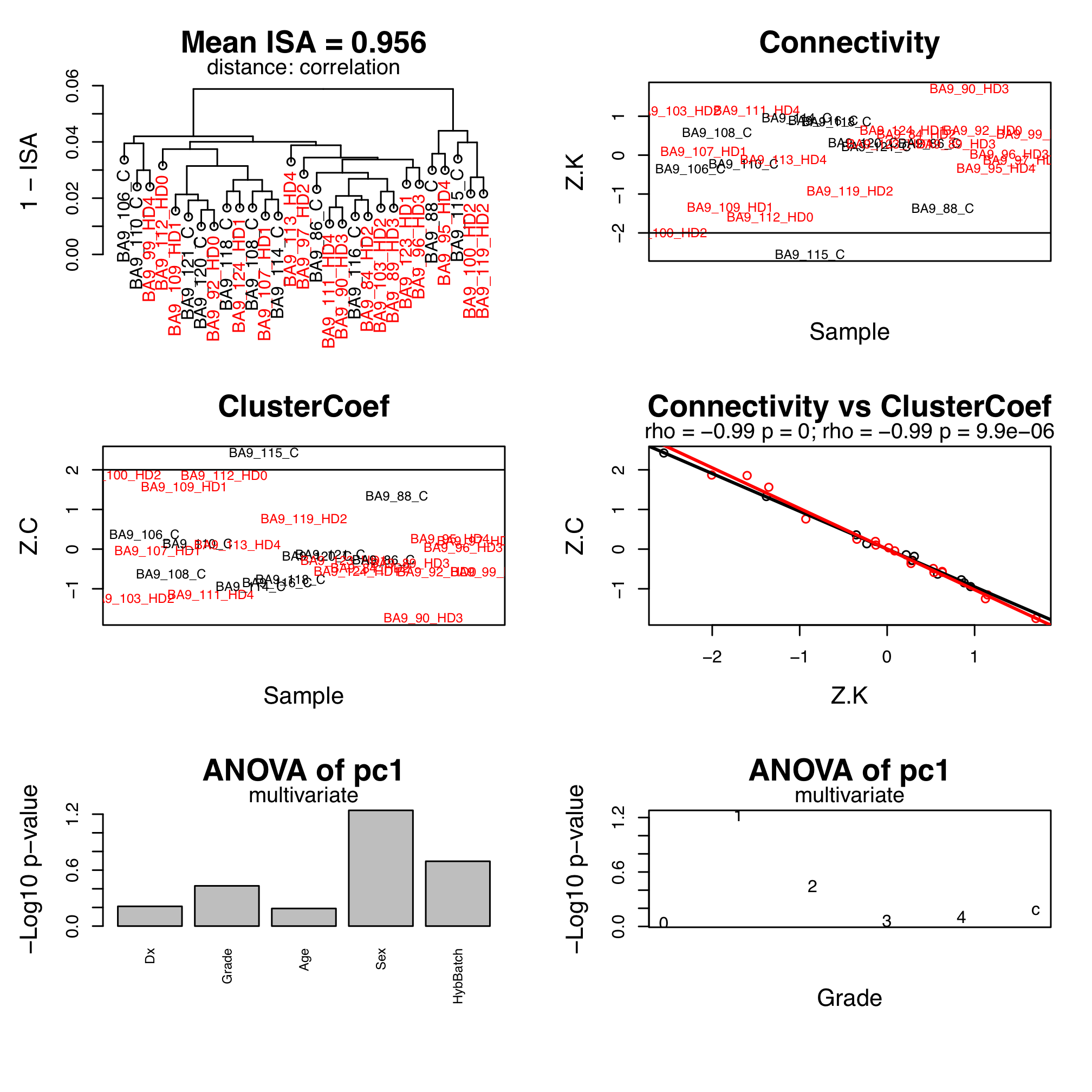


## Now removing two samples with Z.K < -2:

Response: <-2


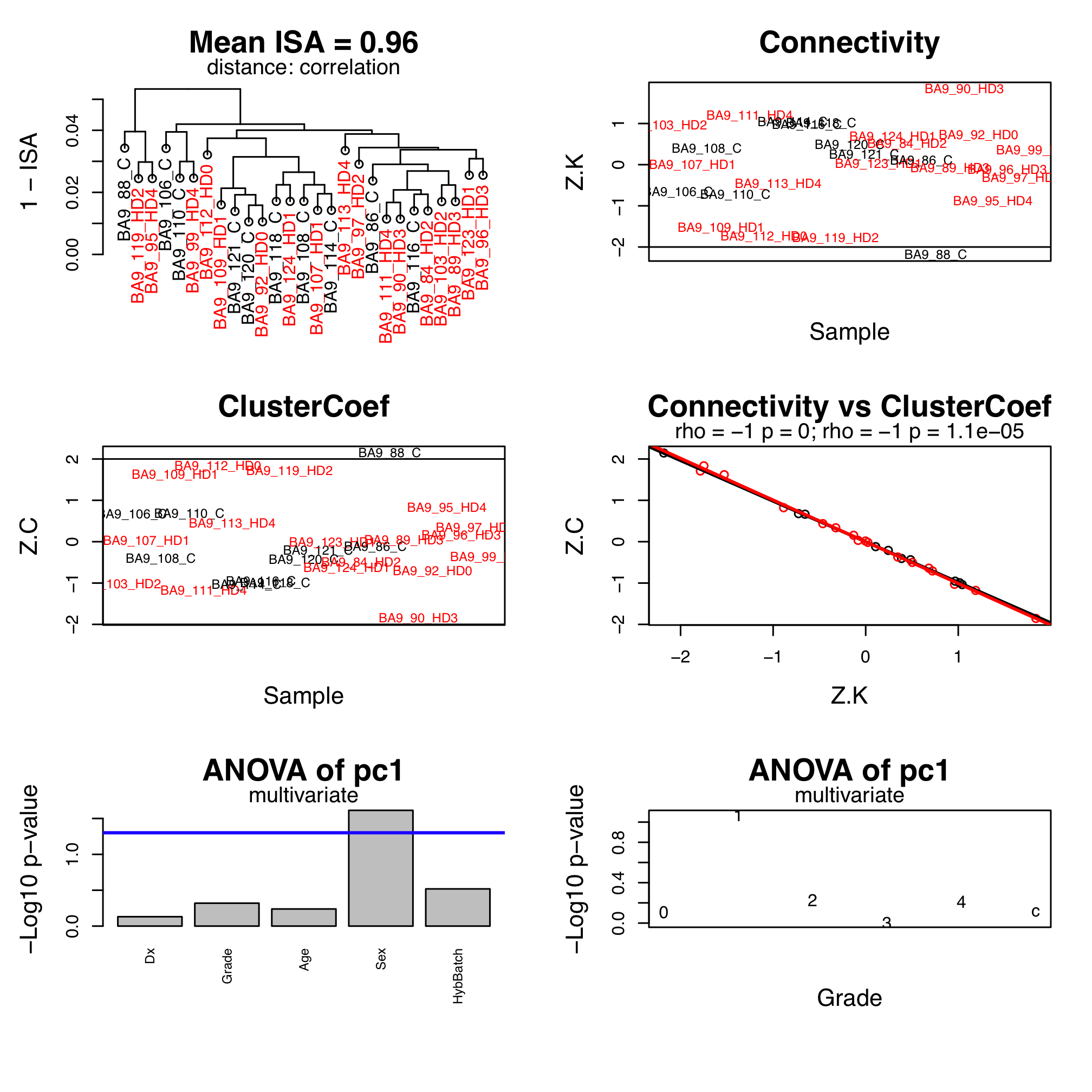


## Removing another sample with Z.K < -2:

Response: <-2


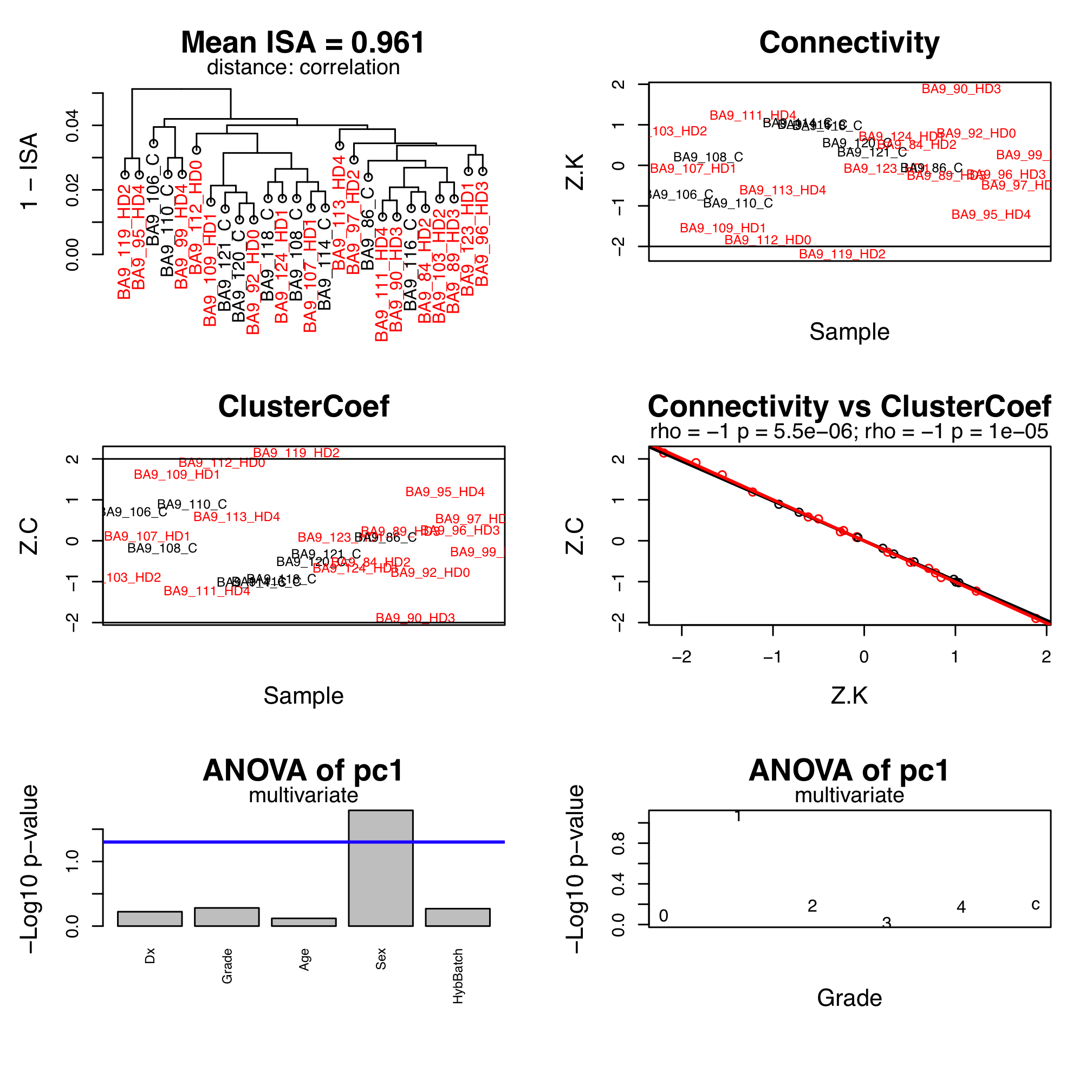


## And one more:

Response: <-2


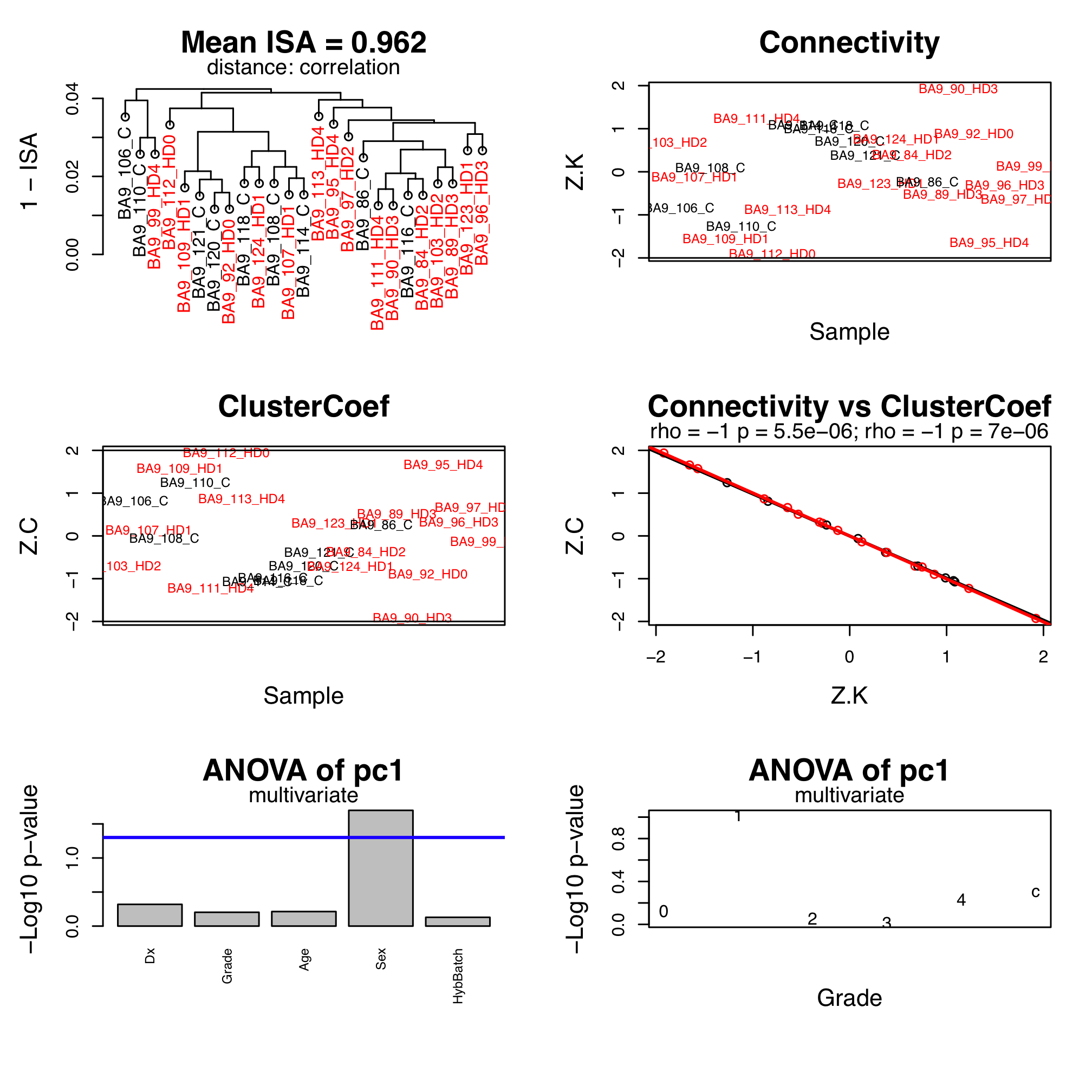


## As evidenced by the mean ISA and cor(K,C), this is now a very clean and homogeneous sample network. Performing quantile normalization:

Response: None


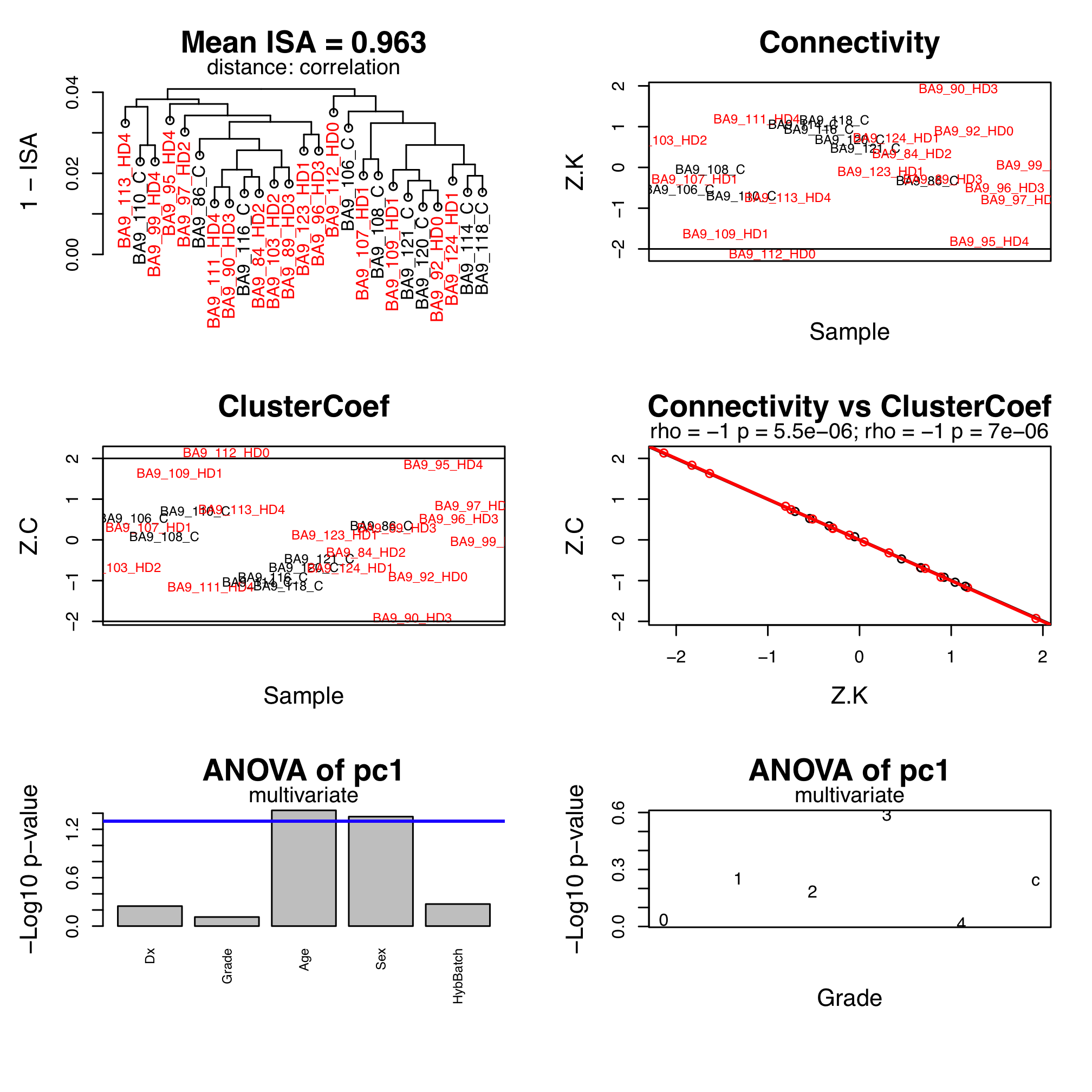


## There is no significant effect of HybBatch on pc1, so we will pass on ComBat:

Response: None

## We are informed:

[1] "BA9 processing complete. Hit enter to proceed to next group."

Press enter:

## After pressing enter, we are told:

[1] "Exporting outlier summary..."

## And we are done!

**SampleNetwork: exported files**

# SampleNetwork creates a subdirectory inside of the working directory of the R session to store all exported files. The name of the subdirectory is:

/projectname1_SampleNetworks

# where projectname1 is a character string specified by the user as an argument of the SampleNetwork function. Within this subdirectory, two .csv files are exported. Using examples from the preceding analysis, these are (where file names are in the form of projectname1_grouplabels1-column-header_*outliers* OR *dataset* metrics_timestamp.csv):

1) GSE3790_Region_outliers_18-28-48.csv

# File 1 contains information on all of the outlying samples that were removed from CN, CB, BA4, and BA9, including sample traits, the processing round in which the sample was removed, sample metrics (Z.K, Z.C, etc.), and the sample row number. This file enables the user to recreate the exact outlier removal process at a later date, if necessary.

2) GSE3790_Region_dataset_metrics_18-28-48.csv

# File 2 contains sample network summary metrics for CB, CN, BA4, and BA9 for each round of processing. These metrics include the number of samples, the mean sample correlation (IAC), mean connectivity, mean scaled connectivity, mean clustering coefficient, mean maximum adjacency ratio (MAR), density (i.e. mean intersample adjacency, or ISA), decentralization, homogeneity, and the percentage variance explained by the first principal component (PC1_VE; see Materials and Methods and Text S1 from the journal article for additional details on these statistics).

# In addition to these files, a subdirectory is created for each group of samples. If you have followed the tutorial from the beginning to this point, you will find five subdirectories in the root projectname1_SampleNetworks/ directory. They are named in the form:

1) All_timestamp

2) BA4_timestamp

3) BA9_timestamp

4) CB_timestamp

5) CN_timestamp

# Since we initially processed all samples simultaneously, this created the "All_timestamp" subdirectory. However, we quickly abandoned this approach in favor of constructing sample networks by brain region, which created the other four subdirectories. Within each subdirectory you will find all of the figures that were generated during each iteration of sample network construction (in the case of the All_timestamp subdirectory, only one figure was generated before we hit ESC, so that is the only file present). You will also find the processed expression (feature) data, as well as a number of other files. Let us walk through the contents of the CN_timestamp subdirectory to orient ourselves. There are 14 files in the CN_timestamp subdirectory, which we have ordered chronologically and annotated below:

1) CN_rd_1_pc1.pdf

2) CN_rd_2_pc1.pdf

3) CN_rd_3_pc1.pdf

4) CN_rd_4_pc1.pdf

5) CN_rd_5_pc1.pdf

## Files 1–5 are .pdf images of the plots generated during each successive round of sample network processing, i.e. CN_rd_1_pc1.pdf is the initial CN sample network, CN_rd_2_pc1 is the second CN sample network (after removing the first round of outlying samples), etc.

6) GSE3790_CN_Qnorm.csv

## File 6 is the quantile normalized CN expression data; quantile normalization is performed after the outlying samples have been removed.

7) GSE3790_CN_66_outliers_removed.csv

## File 7 is a subset of the original expression data (datE), with outlying samples removed but no quantile normalization performed; if the user wishes to perform another type of normalization besides quantile, this file would serve as the input data.

8) CN_rd_Qnorm_pc1.pdf

## File 8 is the .pdf image of the CN sample network plots generated after performing quantile normalization.

9) GSE3790_CN_66_ComBat_sampleinfo.txt

## File 9 is the sample information file that is automatically created by SampleNetwork for use by ComBat. This file contains the sample batch and covariate structure used for batch normalization (if performed).

10) CN_rd_ComBat_pc1.pdf

## File 10 is the .pdf image of the CN sample network plots generated after performing ComBat batch normalization (if performed). In this case, these plots depict the final sample network.

11) CN_change_by_round.pdf

## File 11 is a .pdf image of the changes in sample network metrics with each successive round of data processing. This file is described more below.

12) CN_final_adjmat.csv

## File 12 is the final sample adjacency matrix for the fully processed data (in this case, outliers removed, quantile-normalized, and batch-corrected). This file can be used as input for other clustering methods / network algorithms.

13) GSE3790_CN_66_ComBat.csv

## File 13 contains the fully processed expression data (in this case, outliers removed, quantile-normalized, and batch-corrected). This is the expression data we would want to use for downstream applications (e.g. differential expression analysis, coexpression analysis, etc.).

14) GSE3790_CN_66_final_sample_metrics.csv

## File 14 contains sample traits and node-based sample network indices (e.g. Z.K, Z.C) for all of the samples that have been retained after processing, using the fully processed (normalized and batch-corrected) data.

**SampleNetwork: additional illustration of benefits and conclusions**

The beneficial effects of using the SampleNetwork approach are clearly delineated by significance testing of sample covariates with respect to sample metrics, analysis of network concepts with each successive round of data processing, and analysis of differential expression.

As shown above, technical sources of variation are often present in genomic experiments, and proper data normalization strategies are essential for revealing the full extent of biological effects of interest (e.g. the effect of Dx on pc1 in CN). SampleNetwork does not provide any new normalization strategies; rather, it provides a convenient wrapper for performing quantile normalization [5] and correction for batch effects via ComBat [2]. We find that it is best to execute these strategies after identifying and removing outlying samples. In practice, others and we have found these strategies to be extremely effective at removing systematic technical sources of variation from genomic data. Following outlier detection and removal, SampleNetwork facilitates the execution of these strategies and allows the user to observe how they affect the significance of measured sample traits in relation to summarized feature data.

With each successive round of data processing, SampleNetwork recalculates network summary statistics. These statistics are exported as a table (e.g. GSE3790_Region_dataset_metrics_18-28-48.csv above), but also as a figure that illustrates the changes in these statistics by round. Here is an example (CN_change_by_round.pdfs, i.e. File #11 above):


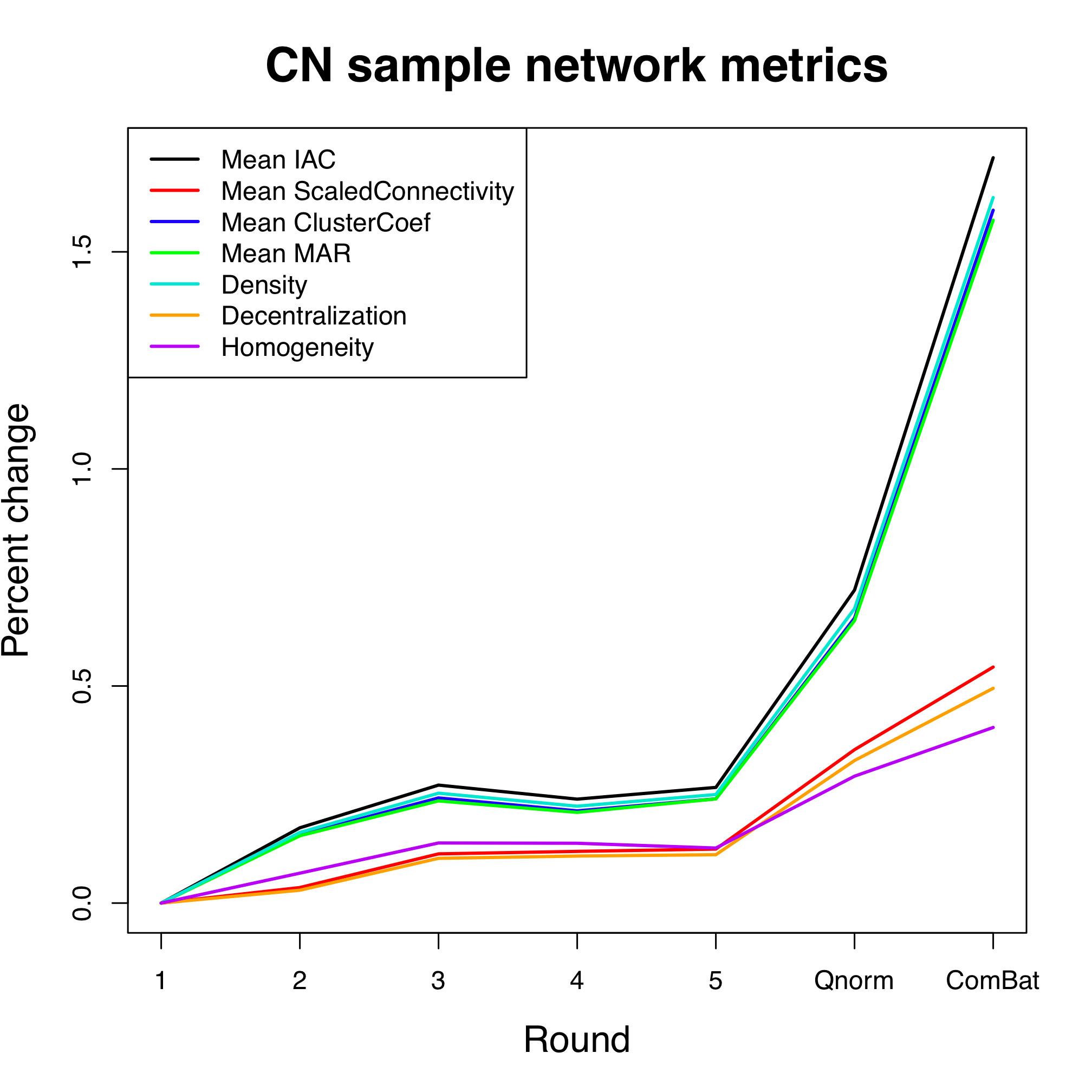


As shown above, there is a modest improvement in sample network summary statistics following outlier removal, and then a much steeper improvement following quantile and ComBat normalization.

Another way to gauge the impact of data processing as implemented via SampleNetwork is simply to calculate the number of differentially expressed genes before and after processing. To this end, we make use of the standardScreeningBinaryTrait R function from the WGCNA package [6], which calculates widely used statistics for relating the columns of the input data frame (i.e. expression data) to a binary sample trait (in this case, Dx). These statistics include Student's *t*-test P-value and the corresponding local false discovery rate (*Q*-value). Below we examine the number of genes (probe sets) that are differentially expressed (*P* (*Q*) < .001) between CN CTRL and CN HD samples before (PRE) and after (POST) processing via SampleNetwork:


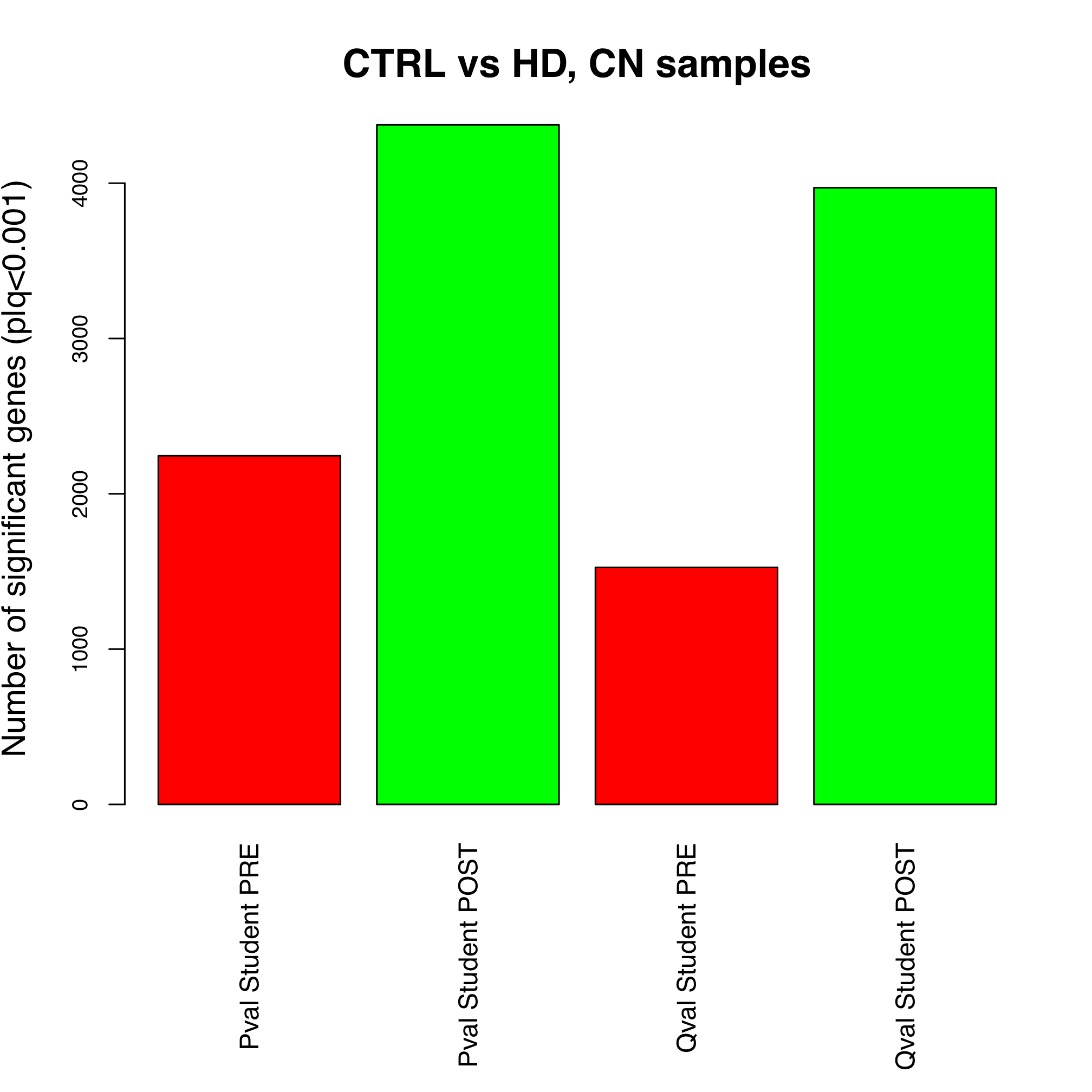


As seen above, the number of differentially expressed genes effectively doubles after data processing via SampleNetwork.

In summary, sample networks can enhance the results of cluster analysis and facilitate a variety of common genomic data processing tasks. SampleNetwork provides a flexible and efficient approach that enables users to move quickly through large datasets in an iterative fashion, specify groups of samples for processing, identify and remove outliers, test the significance of sample covariates, perform data normalization, and remove batch effects. Questions or comments are welcome and should be addressed to [oldhamm@stemcell.ucsf.edu](mailto:oldhamm@stemcell.ucsf.edu).

As described in the journal article, sample networks also provide a novel perspective on group comparisons by exploring connectivity patterns among biologically relevant subgroups of samples. We have explored this theme in greater detail through the creation of a companion R function called ModuleSampleNetwork, which is described below.

**ModuleSampleNetwork**

ModuleSampleNetwork was designed to facilitate comparisons of sample network properties among subgroups of samples when formed over biologically meaningful subsets of features. We were motivated to create ModuleSampleNetwork after observing the divergence in cor(K,C) between CTRL samples and HD samples from CN (Figures 2 and S1 from the journal article). This divergence was initially measured over all genes; we therefore hypothesized that this divergence might vary among subsets of genes that are naturally coexpressed in human CN [7], and created the ModuleSampleNetwork R function to formally test this hypothesis.

Some of the plots generated by ModuleSampleNetwork are similar to those generated by SampleNetwork. However, ModuleSampleNetwork does not enable outlier detection and removal, normalization, or batch correction; instead, it seeks to compare module sample network properties between subgroups of samples and across subsets of features. In our application, we define subsets of features as modules of coexpressed genes, but note that many other designations (e.g. gene ontology, signaling pathways, protein complexes, etc.) could also be used.

Compared to SampleNetwork, ModuleSampleNetwork requires different input files and user interactions, and produces a number of different outputs. These outputs attempt to summarize a variety of analyses, many of which are still experimental in nature. We include the source code and sample files for running ModuleSampleNetwork on our web site for the sake of completeness, but caution the user that ModuleSampleNetwork is still in development and is therefore less robust than SampleNetwork; consequently, the usage information provided below is comparatively sparse.

**Getting the software and the data**

1. Download the R software at <http://www.r-project.org/> and install it on your computer. After installing R, you will need to install the following R library packages: qvalue, gtools, geneplotter, and WGCNA. To install the packages gtools, qvalue and WGCNA, open R, go to the "Packages and Data" menu and select "Package Installer...", then select these three packages; R will automatically install them. The geneplotter package is from Bioconductor (<http://www.bioconductor.org/>). To install this package, open an R session and type:

source("http://bioconductor.org/biocLite.R")

biocLite()

This will install a core selection of Bioconductor packages. To install the geneplotter Bioconductor package, type:

biocLite("geneplotter")

1. Download the .zip file "ModuleSampleNetwork files" at: http://www.genetics.ucla.edu/labs/horvath/CoexpressionNetwork/SampleNetwork. There are three .csv files in this .zip file:

- GSE3790_CN_66_ComBat.csv: This file contains the processed (outlying samples removed, quantile-normalized, and batch-corrected) gene expression data for CN samples from "the HD study" [3], which have been analyzed using the SampleNetwork function as described above (this is file # 13).
- GSE3790_CN_66_final_sample_metrics.csv: This file contains CN sample traits and node-based sample network statistics (e.g. Z.K, Z.C) for all of the CN samples that have been retained after processing via SampleNetwork, as described above (this is file #14).
- CN_modules.csv: This file contains gene coexpression module assignments from an analysis of human CN [7]. Any file consisting of feature IDs (column 1) and feature groupings (column 2) will work.

1. Download the ModuleSampleNetwork R functions file at: http://www.genetics.ucla.edu/labs/horvath/CoexpressionNetwork/SampleNetwork. This file contains the ModuleSampleNetwork_0.5 R function and several other required functions. In this file you will also find a brief description of the arguments for the ModuleSampleNetwork function. DISCLAIMER: Absolutely no warranty on the code. Please contact MCO ([oldhamm@stemcell.ucsf.edu](mailto:oldhamm@stemcell.ucsf.edu)) with bug reports or suggestions.
2. Unzip ModuleSampleNetworkFiles.zip and place the files in the same directory as ModuleSampleNetwork_0.5.r, e.g.: "/Users/mcoldham/Documents/ModuleSampleNetworkTutorial"

**ModuleSampleNetwork: usage and defaults**

ModuleSampleNetwork requires three input files: (1) a file containing the feature activity (e.g. gene expression data; see GSE3790_CN_66_ComBat.csv above for an example), (2) a file containing sample information (i.e. sample traits; see GSE3790_CN_66_final_sample_metrics.csv above for an example), and (3) a file containing feature grouping information (e.g. gene coexpression module assignments; see CN_modules.csv above for an example). In this example, files #1 and #2 were generated by the SampleNetwork function (see above), while file # 3 was generated from a previous analysis of gene coexpression relationships in adult human CN [7]. The usage and default settings of the ModuleSampleNetwork_0.5 R function are described below, followed by a brief description of each argument and its corresponding options.

**Usage**

ModuleSampleNetwork(

datExprT,

method1="correlation",

impute1=FALSE,

skip1,

indices1,

modules1,

featurelabels1,

sampleinfo1,

subgroup1,

samplelabels1,

grouplabels1,

fitmodels1=FALSE,

whichmodel1="univariate",

whichfit1="pc1",

btrait1=NULL,

trait1=NULL,

asfactors1=NULL,

projectname1,

cexlabels1=0.7,

verbose=TRUE,

minGpSize1=10,

corType1="s",

logT,

removeGrey=TRUE

)

**Arguments**

datExprT Expression data (or other types of data) in the form of a data frame where rows are features (e.g. probe sets) and columns are samples.

method1 Controls which type of similarity measure will be used to define a measure of distance among samples. Choices are "correlation" (Pearson; default) or "euclidean" for Euclidean distance. Any other value will trigger an error.

impute1 If TRUE, expression data will be checked for the presence of NA entries; if present, numerical data will be imputed for these entries, using the function impute.knn. The function impute.knn uses a fixed random seed giving repeatable results. If FALSE (default), no imputation will be performed.

skip1 An integer describing the number of feature information columns in datExprT (e.g. probe set ID, gene symbol). Note: there must be at least one column of feature information (unique identifiers) in datExprT.

indices1 A list of vectors that indicates if there are groups of samples to be processed separately by ModuleSampleNetwork. Each vector is used to subset the samples (columns) in datExprT.

modules1 Feature category information in the form of a data frame where rows are features and two columns denote (1) unique feature identifiers (e.g. probe set IDs, or whatever unique feature identifier appears in datExprT), and (2) categories of features (e.g. coexpression module assignments in the form of colors, numbers, etc.).

featurelabels1 A vector equal in length to the number of features, containing convenient feature labels, which will appear in some plots.

sampleinfo1 Sample traits in the form of a data frame where rows are samples and columns are traits.

subgroup1 An integer that points to the column number in sampleinfo1 that specifies the sample subgroups to be compared; these subgroups will be colored separately in plots.

samplelabels1 An integer that points to the column number in sampleinfo1 containing the sample labels that will appear in plots. Note: these sample labels must be identical to the sample column headers in datExprT or an error will be triggered.

grouplabels1 An integer that points to the column number in sampleinfo1 that contains labels for the sample groups specified by indices1. Note: the number of unique group labels in this column must equal the number of vectors (indices) specified by indices1.

fitmodels1 Logical: should linear regression models be constructed and the significance of sample traits assessed with respect to a summary measure of datExprT? If FALSE (default), the number of panels in each plot will be reduced. If TRUE, analysis of variance (ANOVA) will be used to assess the significance of specified sample traits.

whichmodel1 If fitmodels1 = TRUE, should a univariate (default) or multivariate linear regression model be constructed?

whichfit1 If fitmodels1 = TRUE, which summary measure of datExprT should be used as the outcome for model-fitting? Choices are pc1 (default; i.e. the first principal component obtained by singular value decomposition), mean (sample means), and K (sample connectivities).

btrait1 If fitmodels1 = TRUE, a vector of integers that specifies the column numbers (traits) in sampleinfo1 that should be included in model-fitting; otherwise, NULL.

trait1 If fitmodels1 = TRUE, a vector of integers that specifies the column numbers (traits) in sampleinfo1 that should be tested for the significance of individual factor levels in model-fitting; otherwise, NULL. Note: trait1 must be a subset of btrait1 and can specify only categorical variables.

asfactors1 If fitmodels1 = TRUE, a vector of integers that specifies the column numbers (traits) in sampleinfo1 that should be treated as factors (i.e. categorical variables) in model-fitting.

projectname1 Character string that provides a convenient label for the project; this string will appear as part of the filename for some output files.

cexlabels1 Numeric scaling factor for sample labels in plots (default = 0.7).

verbose Logical: should additional network metrics (Z.C) and cor(K,C) be reported and appear in plots? If FALSE, only hierarchical clustering of samples and Z.K will appear. Default = TRUE.

minGpSize1 Integer that specifies the minimum number of samples that must comprise a subgroup. Default = 10.

corType1 Specifies the type of correlation to be used when calculating cor(K,C); choices are "s" (Spearman; default), "p" (Pearson), and "k" (Kendall).

logT Logical: should feature activity levels be log_2_-transformed prior to differential expression (activity) analysis?

removeGrey Logical: should unassigned features that are denoted by the label grey in modules1 be excluded from analysis? By WGCNA convention, unassigned features (e.g. genes) are denoted by the module color grey. If TRUE (default), grey features (as specified in modules1) will be excluded from analysis.

**Details**

ModuleSampleNetwork does not enable outlier testing and removal or data normalization, but instead seeks to facilitate comparisons of sample network properties among subgroups of samples when formed over biologically meaningful subsets of features (see Figures 4 and 6 from the corresponding journal article for examples of ModuleSampleNetwork output). Subsets of features may correspond to modules of coexpressed genes [7] or other gene (feature) categories, such as ontological classifications, signaling pathways, chromosomes, etc. To derive maximum utility from the function, we prefer to apply ModuleSampleNetwork to gene expression data that have been fully processed (i.e. outlying samples removed, normalized, and batch-corrected, as necessary). If necessary, missing data can be inferred by setting the argument impute1 = TRUE.

If feature subsets are coexpression modules, the same expression data that were used to identify the coexpression modules should serve as input (datExprT) for ModuleSampleNetwork. An example workflow would involve using the SampleNetwork function to pre-process a microarray dataset, then using WGCNA [6] to identify modules of coexpressed genes, and finally using the ModuleSampleNetwork function to explore sample network properties at the modular level.

The user can process all samples simultaneously or in groups, as indicated by the argument grouplabels1, which specifies a column number in sampleinfo1. For example, if the user wishes to process all samples simultaneously, the column specified by grouplabels1 would simply contain the same value for all samples (e.g. column 1 of GSE3790_CN_66_final_sample_metrics.csv). Using the HD study as an example, if the user wished to process male and female CN samples separately, this would be done by setting grouplabels1 = 6 (i.e. column 6 of GSE3790_CN_66_final_sample_metrics.csv).

Subgroups of samples to be compared with one another are specified via the argument subgroup1, which is an integer that identifies the column number of sampleinfo1 containing the sample subgroup assignments. Sample networks will be constructed for all sample subgroups that contain at least minGpSize1 samples, and properties of the resulting sample networks will be compared. Using the HD study as an example, if the user wished to compare CN module sample network properties among various grades of HD (where the grades are 0, 1, 2, 3, 4, and c) with minGpSize1 = 10, only three of the grades would meet this requirement: 1 (n = 11), 2 (n = 16), and c (n = 31). Therefore, three comparisons of module sample networks would be produced: c vs 1, c vs 2, and 1 vs 2 (as illustrated below).

The argument method1 currently implements two types of similarity measures for constructing sample networks: correlation and Euclidean distance. In practice, we find it advantageous to use a correlation-based distance measure (the default) for constructing sample networks, for reasons described in the journal article and above. Although we have not thoroughly investigated the properties of sample networks constructed using Euclidean distance, there may be situations in which it is preferable to use Euclidean distance as a similarity measure.

Model-fitting requires that sample traits of interest are present in the sample information file.

When fitting linear models, ModuleSampleNetwork will exclude wholly redundant factor levels and factor levels with only one constituent (see above). In these cases, warning messages will be produced. It is important to specify categorical variables as such via the argument asfactors1 to ensure the accuracy of linear models. We recommend using pc1 (the default) as the summary measure (outcome) for linear models. In practice, we have found that P-values obtained using mean (mean expression) are very similar to those obtained via pc1, but slightly less significant. Linear models constructed using K (connectivity) as the outcome often produce different results.

ModuleSampleNetwork performs a series of analyses. First, sample networks are constructed for all specified subsets of features and sample network properties are compared between all specified subgroups of samples. Second, linear regression models are constructed for each feature category with Z.C regressed upon the additive and interactive effects of Z.K and the sample trait being used to define sample subgroups. Third, eigensamples (see Text S1 from the journal article) are calculated for each feature category and sample subgroup, and the differences among the eigensamples are calculated. Fourth, differential expression and differential connectivity (i.e. differences in kME values [7,8,9]) are assessed for all features that have been assigned to categories. Differential expression is assessed via the R function standardScreeningBinaryTrait from the WGCNA package [6]. These analyses are discussed further below.

**Starting the R session**

Open the R software by double-clicking the desktop icon. To interact with the R software, copy and paste commands into the R console. There are a number of good introductory R tutorials that can be found simply by Googling "R tutorial".

Note: Text written after the "#" character is interpreted as a comment and is automatically ignored by R.

# Set the working directory of the R session to the directory containing the downloaded files, e.g.:

setwd("/Users/mcoldham/Documents/ModuleSampleNetworkTutorial")

# Load the ModuleSampleNetwork_0.5 R functions file:

source("ModuleSampleNetwork_0.5.r")

# Note: This file can also be opened with any text editor, and its contents copied and pasted directly into the R session. Note that the required libraries will be loaded automatically by virtue of reading in this file (assuming that the packages are installed).

**Reading in the expression data and sample information file**

datE=read.csv("GSE3790_CN_66_ComBat.csv")

datS=read.csv("GSE3790_CN_66_final_sample_metrics.csv")

datM=read.csv("CN_modules.csv")

# First, check to make sure that all probe sets (features) are in the same order in datE and datM:

all(datE[,1]==datM[,1])

# [1] TRUE

## Check the dimensions of datE:

dim(datE)
# [1] 18631 68

# So datE is a data frame containing 18,631 rows (corresponding to probe sets) and 68 columns (corresponding to 66 CN samples + 2 feature information columns: "Probe_set" and "Gene").

# Check the dimensions of datS:

dim(datS)

# [1] 66 12

# So datS is a data frame containing 66 rows (corresponding to samples) and 12 columns (corresponding to sample labels, traits, and sample group information). We can see the column headers for datS by typing:

colnames(datS)

# [1] "Region" "Label" "Dx" "Grade" "Age" "Sex" "Country" "HybBatch" "K" "Z.K" "Z.C" "Z.MAR"

# Note that one of the columns in datS must provide the sample labels, which in turn must match the sample column headers in datE identically. In this case, the second column ( "Label") contains the sample labels.

# Let's reorder the samples in datE and datS by diagnosis, then grade, then age:

ordersamples=order(datS$Dx,datS$Grade,datS$Age)

datEnew=datE[,3:68]

datEnew=datEnew[,ordersamples]

datE=data.frame(datE[,1:2],datEnew)

datS=datS[ordersamples,]

# Checking to make sure that samples are in the same order between datE and datS:

all(colnames(datE)[3:68]==datS$SampleLabel)

# [1] TRUE

# Note: It is a good idea to provide brief but informative sample labels. For example, in this case, our sample labels take the form CN_14_C, CN_16_HD0, etc., where the first field (e.g. CN) denotes the brain region, the next field denotes the individual (14 or 16), and the next field denotes the diagnosis (control or Huntington's disease). Note as well the number after HD (e.g. HD0), which denotes the grade of HD severity (e.g. grade 0).

**Running the ModuleSampleNetwork function: user interactions**

We will illustrate the ModuleSampleNetwork function by analyzing CN samples from the HD study that have been processed using the SampleNetwork function as described above. Sample networks will be constructed for 23 subsets of genes, each of which corresponds to a gene coexpression module previously identified in CTRL subjects from human CN [7]. We will first compare module sample network properties between CTRL and HD CN samples. Subsequently, we will also illustrate how the user can instruct ModuleSampleNetwork to compare module sample network properties among discrete grades of HD. ModuleSampleNetwork may be executed by copying and pasting the following code into the R session:

ModuleSampleNetwork(

datExprT = datE,

method1="correlation",

impute1 = FALSE,

skip1 = 2,

indices1 = list(c(3:68)),

modules1 = datM,

featurelabels1 = as.character(datE[,2]),

sampleinfo1 = datS,

subgroup1 = 3,

samplelabels1 = 2,

grouplabels1 = 1,

fitmodels1 = TRUE,

whichmodel1 = "multivariate",

whichfit1 = "pc1",

btrait1 = c(3,4,5,6,7,8),

trait1 = NULL,

asfactors1 = c(3,4,6,7,8),

projectname1 = "GSE3790",

cexlabels1 = 0.7,

verbose = TRUE,

minGpSize1=10,

corType1="s",

logT = TRUE,

removeGrey=TRUE

)

## Let us briefly describe the parameters specified above in English:

## We will construct module sample networks using expression data contained in datE (datExprT=datE) and correlation as a distance measure among samples (method1="correlation"). There are no missing data (impute1=FALSE). The first two columns of datE contain feature information (skip1=2), and we will process all samples simultaneously (indices1=c(3:68)). Feature category information (in this case, modules of coexpressed genes) is contained in datM (modules1=datM), while convenient feature labels that will appear in some plots (in this case, gene symbols) are present in the second column of datE and denoted by featurelabels1=as.character(datE[,2]). datS specifies the sample information file (sampleinfo1=datS). We will compare module sample network properties for the subgroups that correspond to the categories of Dx (diagnosis, i.e. CTRL vs HD), as specified by subgroup1, which points to the third column of datS (the Dx column); in addition, samples will be colored in plots according to diagnosis. The sample labels in datS are in column 2 (samplelabels1=2), and the group labels (in this case, "CN", since we are processing all CN samples simultaneously), are in column 1 (grouplabels1=1). We will perform multivariate linear regression analysis (fitmodels1=TRUE) and (whichmodel1="multivariate") using the first principal component of datE as the outcome measure (whichfit1="pc1"). We will include the sample traits that are present in columns 3, 4, 5, 6, 7 and 8 of datS, in that order, for building the model (btrait1=c(3,4,5,6,7,8)). We will not test the significance of the individual levels of any categorical variable in datS (trait1=NULL). Among the sample traits we are including in the model, all but one (column 5) should be modeled as categorical variables (factors; asfactors1=c(3,4,6,7,8)). Note: it is very important that categorical variables be indicated as such via asfactors1 for accuracy in the modeling process. The name we will give the project is "GSE3790" (projectname1="GSE3790"), and the default setting for the size of sample labels in the plots will be retained (cexlabels1="GSE3790"). Additional plots of Z.C and cor(K,C) will be produced (verbose=TRUE). We will require that sample subgroups have at least 10 members (minGpSize1=10) in order to construct module sample networks. cor(K,C) will be assessed using the Spearman correlation (corType1="s"), and expression data will be log_2_-transformed prior to differential expression analysis. Features denoted by the color "grey" in the second column of modules1 will be excluded from the analysis (removeGrey=TRUE).

## These parameters direct ModuleSampleNetwork to begin. First, sample networks are constructed for all subsets of features described in modules1. Then, the user is prompted for information:

[1] "Building linear models..."

keptgps

1 C

2 HD

[1] The groups listed above will be retained. Please place them in the desired order for figures using the row numbers listed above (e.g. 2,1,3).

## The user can choose the order in which sample subgroups will appear in some of the exported files in the /Linear_models subdirectory; try experimenting with both options to see the results. Here we will choose CTRL first, then HD:

Response: 1,2

## The user is then prompted for an additional piece of information:

[1] "Comparing correlations of Z.C and Z.K..."

[1] C HD

Levels: C HD

[1] Please choose reference group for eigensample contrast plots.

## The user can choose the order in which sample subgroups will appear in some of the exported files in the /Module_eigensamples subdirectory; try experimenting with both options to see the results. Here we will choose CTRL as the reference group:

Response: C

## The function continues to update its progress as it proceeds through comparisons of sample network concepts, comparisons of differential expression and differential connectivity, and performance of standard screening. Let us now examine the directory structure and files that are exported by ModuleSampleNetwork.

**ModuleSampleNetwork: exported files**

# ModuleSampleNetwork creates a subdirectory inside of the working directory of the R session to store all exported files. The name of the subdirectory is:

/projectname1_ModuleSampleNetworks

# where projectname1 is a character string specified by the user as an argument of the ModuleSampleNetwork function (in this case, "GSE3790_ModuleSampleNetworks"). Within this subdirectory, there is another directory that is named in the form:

/grouplabels1_x_subgroup1_timestamp

# or in this case, "CN_x_Dx_13-32-09". Within this directory there are four files and four additional directories, which we will discuss below in chronological order:

1) /Sample_network_plots

# This directory contains sample networks summaries for each feature category (in this case, 23 gene coexpression modules). These figures are similar to those produced by the SampleNetwork function, with the obvious exception that they are formed over subsets of features. In addition, the bottom right panel depicts a heat map of activity values (e.g. gene expression) for the feature subset. Note that the file GSE3790_CN_x_Dx_SampleNetwork_salmon.pdf is very similar to Figure 6 from the journal article.

2) GSE3790_CN_x_Dx_network_metrics_all_samples.csv

# This file contains sample network summary statistics for all feature subsets. These statistics include the mean sample correlation (IAC), mean connectivity, mean scaled connectivity, mean clustering coefficient, mean maximum adjacency ratio (MAR), density (i.e. mean intersample adjacency, or ISA), decentralization, homogeneity, and the percentage variance explained by the first principal component (PC1_VE; see Materials and Methods and Text S1 from the journal article for additional details on these statistics).

3) GSE3790_CN_x_Dx_network_metrics_by_sample.csv

# This file contains trait information and node-based network statistics for all samples and all feature subsets. In this case, Z.K and Z.C are reported for all samples and all 23 modules of coexpressed genes.

4) /Linear_models

# This directory contains two files that summarize the results of linear regression analysis. Linear regression models are constructed in the form Z.C~Z.K*subgroup1 (in this case, Dx) to assess the significance (via ANOVA) of Z.K, the sample trait (Dx), and the interaction between Z.K and the sample trait with respect to Z.C. The .pdf file reports P-values for each module and each term of the model. The first figure in this file also reports the P-values of the correlations between the trait specified by subgroup1 (Dx) and the module eigengenes (MEs), which are the first principal components obtained via singular value decomposition of the gene expression in each module (see Text S1 from the journal article). Note that the significance of cor(Dx,ME) is equivalent to the P-value obtained by Students's *t*-test of ME values between CTRL and HD samples. The .csv file within this directory contains the raw values used to generate the figures.

5) GSE3790_CN_x_Dx_C_vs_HD_DSNC_summary.pdf

# This file summarizes the divergence in cor(K,C) and the extent of differential expression seen between sample subgroups over all subsets of features. Significant differences in cor(K,C) between sample subgroups (p.Diff.cor(K,C)) may indicate substantial differences in sample homogeneity, as described in the journal article (note that this figure corresponds to Figure 4 from the journal article).

6) GSE3790_CN_x_Dx_DSNC_summary.csv

# This file reports the correlation between Z.K and Z.C and corresponding significance for all samples and each subgroup of samples over all feature subsets. This file also reports mean Z.C for all samples, each subgroup of samples, and the P-value of differences in Z.C among sample subgroups based upon the Wilcoxon rank sum test (again over all feature subsets).

7) /Module_eigensamples

# This directory contains two types of files, both .pdfs. The first type of file is named in the form *featurecategory*_eigensamples_by_*subgroup1*.pdf (e.g. salmon_eigensamples_by_Dx.pdf). This file depicts barplots of module eigensamples for each sample subgroup and each feature subset. Whereas the module eigengene is a summary of the characteristic gene expression pattern of a module (with each vertical bar corresponding to a sample), the module eigensample is a summary of the characteristic sample expression pattern of a module (with each vertical bar corresponding to a gene in the module; see Text S1 from the journal article for additional details). In our analysis, the color of each bar (gene) corresponds to its strength of module membership, or kME value, which is defined as the Pearson correlation between the expression level of the gene and the module eigengene [7,8,9] (as indicated by the kME scale bar at the top). The second type of file is named in the form *featurecategory*_eigensample_contrasts_by_*subgroup1*.pdf (e.g.

salmon_eigensample_contrasts_by_Dx.pdf)). This file simply depicts the difference between the module eigensamples reported for each sample subgroup.

8) /DE_and_DC

# This directory contains a subdirectory for each sample subgroup comparison (in this example, there is only one: C vs HD). Within this subdirectory are two files and three additional directories. The first file, CN_x_Dx_C_vs_HD_DE_vs_DC_summary.pdf, contains a summary of the extent of differential expression (DE) and differential connectivity (DC) between sample subgroups and across feature subsets. DE is determined using Student's *t*-test (via the standardScreeningBinaryTrait R function of the WGCNA package [6]), and DC, which is defined as the significance of the difference between kME values, is defined as described in ref. [7]. On the left are two summary measures of DE. The top barplot is the significance of the correlation between subgroup designations and the module eigengene, while the bottom boxplot shows the distribution of DE Q-values for genes in each module. On the right are two summary measures of DC. The top barplot reports the Pearson correlations between kME values for genes in each module between sample subgroups. The bottom boxplot shows the distribution of DC Q-values for genes in each module. The second file, GSE3790_CN_x_Dx_SSBT_C_vs_HD.csv, contains the gene-based output of standardScreeningBinaryTrait, along with kME values and DC P-values and Q-values. The DC directory contains scatterplots of kME values for each sample subgroup and each feature subset; features that are colored in red are significantly different between the two sample subgroups, and the linear least squares regression line is depicted in red. The DE directory contains scatterplots of mean activity (i.e. gene expression) levels for each sample subgroup and each feature subset; features that are colored in red are significantly different between the two sample subgroups, and the linear least squares regression line is depicted in red. Finally, the DE_vs_DC directory compares DE vs DC for the genes (features) that comprise each subset (module).

**Running ModuleSampleNetwork for more than two sample subgroups**

ModuleSampleNetwork can be easily extended to perform multiple comparison among sample subgroups in cases where the number of subgroups > 2. For example, if we wish to compare module sample network properties among discrete grades of HD, all we have to do is change the subgroup1 argument to point to the "Grade" column in datS instead of the "Dx" column. Note that by keeping minGpSize1=10 (the default), we exclude grades 0, 3, and 4, which have 2, 5, and 1 samples, respectively. This analysis can be performed by copying and pasting the following R code into the session:

ModuleSampleNetwork(

datExprT = datE,

method1="correlation",

impute1 = FALSE,

skip1 = 2,

indices1 = list(c(3:68)),

modules1 = datM,

featurelabels1 = as.character(datE[,2]),

sampleinfo1 = datS,

subgroup1 = 4,

samplelabels1 = 2,

grouplabels1 = 1,

fitmodels1 = TRUE,

whichmodel1 = "multivariate",

whichfit1 = "pc1",

btrait1 = c(3,4,5,6,7,8),

trait1 = NULL,

asfactors1 = c(3,4,6,7,8),

projectname1 = "GSE3790",

cexlabels1 = 0.7,

verbose = TRUE,

minGpSize1=10,

corType1="s",

logT = TRUE,

removeGrey=TRUE

)

# The user is encouraged to compare the results generated by this analysis to those generated by the preceding analysis.

**Conclusions**

ModuleSampleNetwork provides an array of functionality with the goal of enabling supervised comparisons of sample network properties between subgroups of samples and biologically meaningful subsets of features. Since much of its functionality is still experimental in nature, it should be used in an exploratory fashion. Please direct questions or suggestions to [oldhamm@stemcell.ucsf.edu](mailto:oldhamm@stemcell.ucsf.edu).

**REFERENCES**

1. Zhang B, Horvath S (2005) A general framework for weighted gene co-expression network analysis. Statistical Applications in Genetics and Molecular Biology 4: Article 17.

2. Johnson WE, Li C, Rabinovic A (2007) Adjusting batch effects in microarray expression data using empirical Bayes methods. Biostatistics 8: 118-127.

3. Hodges A, Strand AD, Aragaki AK, Kuhn A, Sengstag T, et al. (2006) Regional and cellular gene expression changes in human Huntington's disease brain. Hum Mol Genet 15: 965-977.

4. Vonsattel JP, Myers RH, Stevens TJ, Ferrante RJ, Bird ED, et al. (1985) Neuropathological classification of Huntington's disease. J Neuropathol Exp Neurol 44: 559-577.

5. Bolstad BM, Irizarry RA, Astrand M, Speed TP (2003) A comparison of normalization methods for high density oligonucleotide array data based on variance and bias. Bioinformatics 19: 185-193.

6. Langfelder P, Horvath S (2008) WGCNA: an R package for weighted correlation network analysis. BMC Bioinformatics 9: 559.

7. Oldham MC, Konopka G, Iwamoto K, Langfelder P, Kato T, et al. (2008) Functional organization of the transcriptome in human brain. Nat Neurosci 11: 1271-1282.

8. Dong J, Horvath S (2007) Understanding network concepts in modules. BMC Syst Biol 1: 24.

9. Horvath S, Dong J (2008) Geometric interpretation of gene coexpression network analysis. PLoS Comput Biol 4: e1000117.
